# African soil properties and nutrients mapped at 30 m spatial resolution using two-scale ensemble machine learning

**DOI:** 10.1038/s41598-021-85639-y

**Published:** 2021-03-17

**Authors:** Tomislav Hengl, Matthew A. E. Miller, Josip Križan, Keith D. Shepherd, Andrew Sila, Milan Kilibarda, Ognjen Antonijević, Luka Glušica, Achim Dobermann, Stephan M. Haefele, Steve P. McGrath, Gifty E. Acquah, Jamie Collinson, Leandro Parente, Mohammadreza Sheykhmousa, Kazuki Saito, Jean-Martial Johnson, Jordan Chamberlin, Francis B. T. Silatsa, Martin Yemefack, John Wendt, Robert A. MacMillan, Ichsani Wheeler, Jonathan Crouch

**Affiliations:** 1 EnvirometriX Ltd, Wageningen, The Netherlands; 2 OpenGeoHub Foundation, Wageningen, The Netherlands; 3 Innovative Solutions for Decision Agriculture Ltd (iSDA), Harpenden, United Kingdom; 4 MultiOne Ltd, Zagreb, Croatia; 5 World Agroforestry (ICRAF), Nairobi, Kenya; 6 Department of Geodesy and Geoinformatics, Faculty of Civil Engineering, University of Belgrade, Belgrade, Serbia; 7 GILAB Ltd, Belgrade, Serbia; 8 International Fertilizer Association (IFA), Paris, France; 9 Rothamsted Research, Harpenden, United Kingdom; 10 Africa Rice Center (AfricaRice), Bouaké, Côte d’Ivoire; 11 International Maize and Wheat Improvement Centre (CIMMYT), Nairobi, Kenya; 12 Sustainable Tropical Solutions (STS) Sarl, Yaoundéc, Cameroon; 13 International Fertilizer Development Center (IFDC), Muscle Shoals, AL USA

**Keywords:** Geochemistry, Machine learning, Biogeochemistry, Mineralogy, Agroecology

## Abstract

Soil property and class maps for the continent of Africa were so far only available at very generalised scales, with many countries not mapped at all. Thanks to an increasing quantity and availability of soil samples collected at field point locations by various government and/or NGO funded projects, it is now possible to produce detailed pan-African maps of soil nutrients, including micro-nutrients at fine spatial resolutions. In this paper we describe production of a 30 m resolution Soil Information System of the African continent using, to date, the most comprehensive compilation of soil samples ($N \approx 150,000$) and Earth Observation data. We produced predictions for soil pH, organic carbon (C) and total nitrogen (N), total carbon, effective Cation Exchange Capacity (eCEC), extractable—phosphorus (P), potassium (K), calcium (Ca), magnesium (Mg), sulfur (S), sodium (Na), iron (Fe), zinc (Zn)—silt, clay and sand, stone content, bulk density and depth to bedrock, at three depths (0, 20 and 50 cm) and using 2-scale 3D Ensemble Machine Learning framework implemented in the mlr (Machine Learning in R) package. As covariate layers we used 250 m resolution (MODIS, PROBA-V and SM2RAIN products), and 30 m resolution (Sentinel-2, Landsat and DTM derivatives) images. Our fivefold spatial Cross-Validation results showed varying accuracy levels ranging from the best performing soil pH (CCC = 0.900) to more poorly predictable extractable phosphorus (CCC = 0.654) and sulphur (CCC = 0.708) and depth to bedrock. Sentinel-2 bands SWIR (B11, B12), NIR (B09, B8A), Landsat SWIR bands, and vertical depth derived from 30 m resolution DTM, were the overall most important 30 m resolution covariates. Climatic data images—SM2RAIN, bioclimatic variables and MODIS Land Surface Temperature—however, remained as the overall most important variables for predicting soil chemical variables at continental scale. This publicly available 30-m Soil Information System of Africa aims at supporting numerous applications, including soil and fertilizer policies and investments, agronomic advice to close yield gaps, environmental programs, or targeting of nutrition interventions.

## Introduction

Predictive Soil Mapping (PSM) aims to produce the most accurate and most objective predictions of soil variables either for bulk estimates or for specific soil depths. PSM, a sub-field of Applied Predictive Modeling^1^, can be considered to be an interdisciplinary field incorporating statistics, soil science and Machine Learning^2–5^. Training points used to build predictive models are usually provided by data from soil samples (fixed depth intervals) or soil profiles (pedogenetic soil horizons) that were geolocated in the field and then entered into a soil profile database. Covariate layers commonly used to train models include terrain attributes^4^—especially hydrological terrain parameters—parent material maps, climatic and vegetation maps and surface reflectances, including bare soil surface reflectances^6^. Predictions of soil properties and classes are generated by (1) training the learners i.e. fitting the spatial prediction models, then (2) applying these fitted models to all pixels so that a complete and consistent map can be produced^1,5^.

Until recently, soil property and class maps for the continent of Africa were only available at very generalised scales^7–9^, with many countries not mapped at all. Considerable soil resources of Africa, especially organic matter^10^ and nutrient stocks^11^ remained largely unmapped and unknown. Fertilizer prices in Africa remain discouragingly high and consequently the efficiency of using fertilizers needs to be clear and considerable before it can be adopted by cash-constrained and risk averse farmers^7,9,12^. It is now possible to produce detailed maps of soil nutrients, including micro-nutrients, due to increasing quantity and availability of soil samples collected at field point locations by various government and/or NGO funded projects: e.g. by projects supported by the National Governments of Ethiopia, Tanzania, Kenya, Uganda, Nigeria, Ghana, Rwanda, Burundi and others; by international donors^13–16^, as well as by the private sector.

The AfSIS project released, in 2017, a gridded Soil Information System of Africa at 250 m resolution showing the spatial distribution of primary soil properties of relatively stable nature, such as depth to bedrock, soil particle size fractions (texture), pH, contents of coarse fragments, organic carbon and extractable elements such as Fe, Ca, Mg, Na, K, Zn, Cu, Mn and Al^17^. The 250 m resolution predictions were later used to estimate large-scale nutrient gaps i.e. fertility zones for major agricultural crops. Berkhout et al.^18^, for example, reported significant relations between these soil nutrient maps and human health as indicated by child mortality, stunting, wasting and underweight.

The initial maps produced in 2017^17^ exhibited several limitations:

- Harmonization of training points (merge from multiple datasets) revealed problems with incomplete metadata which made the data less reliable. Predictions of extractable phosphorous (see Fig. 5 in Hengl et al.^17^), for example, were shown to over-estimate values at multiple locations. Such systematic oscillations usually arise due to incorrect use of measurements units or errors in importing the soil sample data.
- During this earlier predictive soil mapping exercise, spatial clustering of points (i.e. over-representation of specific soil types and landscape positions) were not yet accounted for in the methodology^19^. This possibly introduced a bias in the earlier 250 m scale predictions.
- Predictions were based on the use of relatively coarse resolution covariates only, with limited up-to-date Earth Observation imagery available at that time to help map nutrient content.

We recently re-examined these problems and concluded that a complete redesign and re-implementation of the entire PSM process was required, beginning from point data import and harmonization, into modeling and spatial cross-validation methodologies. Our main hypothesis was that the accuracy of the previous predictions could be much improved if we:

1. Utilize an improved predictive mapping framework: spatially-adjusted Ensemble Machine Learning, that better accounts for spatial clustering of points;
2. Invest more effort into fine-tuning the Machine Learning algorithms: especially to account for spatial clustering of points, and more efficiently subset features of interest;
3. Include in the prediction process new, state-of-the-art, Earth Observation data: especially Sentinel-2 imagery which is available for the entire continent at fine spatial resolutions (10–30 m);
4. Include per pixel error predictions i.e. to quantify prediction uncertainty per pixel.

In addition to redoing the spatial analysis of soil nutrients, we also decided to extend the original list of target soil nutrients^17^ to include soil chemical (pH, eCEC) and physical (bulk density, clay, sand and silt fractions) properties, so that we can produce a more holistic representation of soils.

We present here results of modeling and predicting soil variables for the entire African continent. These are now made available at relatively detailed spatial resolution (30 m), with prediction uncertainty estimates included per pixel. We focus here on the main results and discoveries that could potentially impact any similar continental or global scale soil mapping projects, and then provide detailed explanation of steps followed.

## Results

### Goodness of fit and variable importance

**Figure 1:**
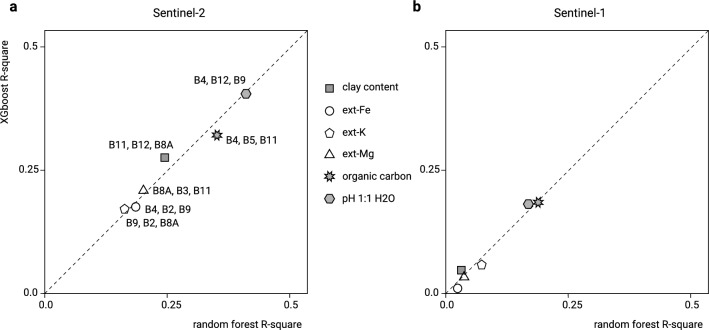
Preliminary predictive modeling R-square based on tenfold cross-validation and modeling selection of soil target variables purely as a function of Sentinel-2 (**a**) and Sentinel-1 (**b**) products. Derived by uploading soil data as points to Google Earth Engine, then overlaying with Sentinel-1/2 and Landsat products and fitting individual models using the caret package^1^ (see also Fig. 7).

The preliminary import of all soil data in Google Earth Engine and subsequent correlation analysis with Sentinel-2 percentiles (for the period 2016–2019), Landsat-8 percentiles (for the period 2013–2019) revealed that there was indeed potential, especially for Sentinel-2 products, to use Earth Observation (EO) data to increase the accuracy of mapping of soil properties and nutrients in Africa. These results clearly indicate predictive potential with the most correlated soil/environmental parameters being soil pH (Sentinel-2 B04, B12, B9), soil organic carbon (Sentinel-2 B04, B05, B11, B12) and clay content determined by laser diffraction method (Sentinel-2 B11, B12, B8A) with respective best R-square based on spatial tenfold cross-validation at 0.38, 0.32 and 0.26 (Fig. 1). For Mehlich3 extractable nutrients and micro-nutrients, Sentinel-2 and Landsat-8 products commonly explained $<25$% of observed variation, but were still significant. In the case of Sentinel-1 products (HH, HV, HH/HV), detectable correlation with soil nutrients, apart from pH and soil organic carbon, was considerably lower to non-existent (Fig. 1). For practical reasons, we ultimately decided to focus on using existing Landsat products^20^ and the Sentinel-2 bands B02 (Blue), B04 (Red), B8A (Narrow NIR), B09 (Water vapour), B11 (SWIR1) and B12 (SWIR2) as the major new environmental covariates, while the Sentinel-1 products were not utilized to produce final predictions.

**Figure 2:**
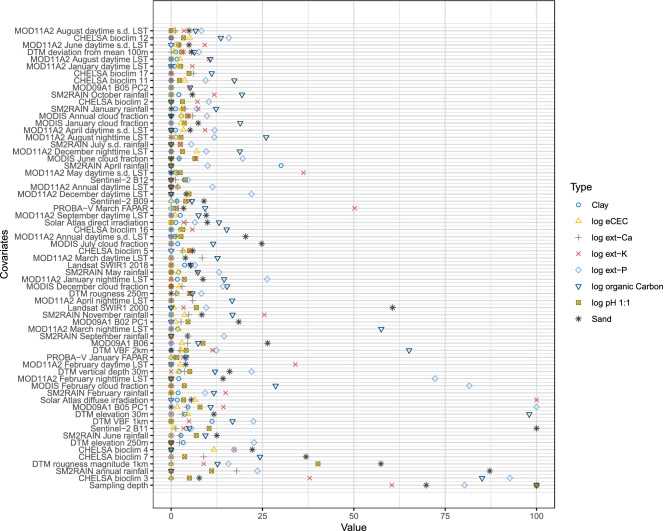
Relative covariate importance for selected target soil variables ordered based on mean importance of all 250 m and 30 m resolution covariates. In this instance, the covariate *“sampling depth”* is the overall most important covariate, while Sentinel-2 B11 and Landsat SWIR images are revealed as the highest ranked covariates at 30 m resolution (see further Table 1 and Supplementary materials).

The combined variable importance plots derived using Random Forest with all 250 m and 30 m covariates used together (Fig. 2) reveal that, on average, climatic images such as SM2RAIN monthly rainfall estimates and CHELSA bioclimatic images (3, 7, 4), are the most important covariates to inform mapping of soil properties and nutrients in Africa. This result is consistent with our previous global results^21^, where soil chemical properties were primarily correlated with climate images, and soil physical properties with a combination of landform parameters, parent material and climatic images. At 30 m resolution, however, Sentinel-2 B11, B09 and B12, DTM vertical depth and Landsat SWIR1 are overall the most important for mapping soil properties and nutrients. Although these covariates appear lower on the full list of the most important variables than climatic images, this is an important discovery and clearly indicates that Sentinel and Landsat seasonal and/or long-term composites merit utilization as covariates for this current, and future, predictive soil mapping campaigns.

**Figure 3:**
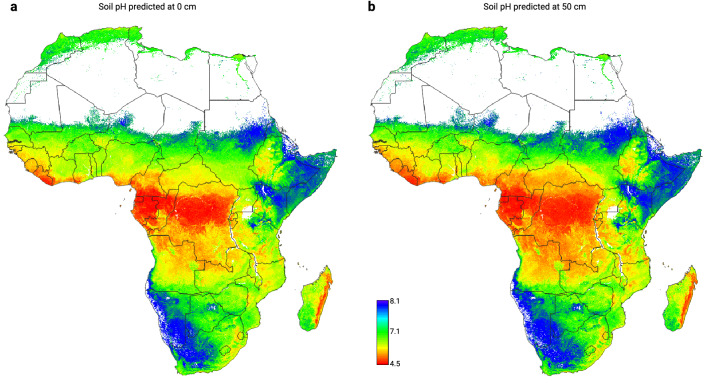
Example of predictions of soil pH 1:1 water suspension at 0 cm (**a**) and 50 cm (**b**) depths whole of Africa. See also Fig. 2. Visualizations produced using QGIS v3.10 (https://www.qgis.org/).

When the importance measures for all variables are ordered based on the mean relative importance (absolute variable importance divided by the highest variable importance), the results show that overall the most important variables for mapping soil properties in Africa are (1) sampling depth (Figs. 2 and 3), (2) Isothermality (quantifies how large the day-to-night temperatures oscillate relative to the summer-to-winter annual oscillations) and (3) mean annual rainfall. Here Isothermality seems to be especially important for modeling log ext.-K, log ext.-Mg and log ext.-S, and mean annual rainfall for modeling organic carbon, organic N, soil pH, log ext.-Mg and log ext.-P (see also Supplementary material).

The newly added USGS surficial lithology map of Africa^22^ did not help improve predictions, however ln-eCEC values were significantly correlated with the class *“Volcanic—Ash, Tuff, Mudflow”*. Classes *“Extrusive Volcanic”* and *“Aeolian Sediments”* seem to marginally help improve predictions of sand and clay. The surprisingly low correlation between the surficial geology map classes and soil nutrients is most likely due to the overly coarse scale of the geological map.

### Accuracy assessment based on fivefold spatial cross-validation

Table 1 shows summary results of fivefold spatial cross-validation for all variables of interest. The average R-square ranges from the best performing soil pH (CCC = 0.90) and ext.-Al (CCC = 0.937), to the worst performing ext.-P (CCC = 0.654), ext.-S (CCC = 0.708) and depth to bedrock (CCC = 0.725). Also note from Table 1 that some variables are predicted using considerably smaller training pools: especially bulk density, depth to bedrock, ext.-S and ext.-Zn have about 2–3$\times$ fewer observations for training than e.g. soil pH or similar. The models for bulk density, depth to bedrock, ext.-S and ext.-Zn are hence, in general, less representative of all landscape combinations in Africa and should be used with caution.

**Table 1:** List of variables provided via iSDAsoil and average accuracy performance based on the fivefold spatial cross-validation (R-square, RMSE and Concordance Correlation Coefficient). Extractable elements are based on Mehlich-3 method. *Statistics based on the ln-transformed values. See also Supplementary material for detailed summary statistics per variable.

Variable	Unit	Training samples	R-square	RMSE	CCC
Sand content	%	122,261	0.736	13.7	0.848
Silt content	%	122,223	0.640	8.92	0.780
Clay content	%	122,269	0.746	9.6	0.854
Bulk density, <2 mm fraction	g/cc	13,565	0.819	126	0.901
Carbon, organic	g/kg	122,457	*0.791	*0.369	0.883
Carbon, total	g/kg	50,140	*0.794	*0.291	0.820
pH in $\hbox {H}_2$O	–	133,378	0.818	0.459	0.900
Stone content	%	92,785	*0.709	*0.803	0.701
Effective Cation Exchange Capacity	cmol(+)/kg	66,380	*0.754	*0.417	0.860
Calcium, extractable	mg/kg	144,593	*0.840	*0.543	0.913
Iron, extractable	mg/kg	57,526	*0.817	*0.235	0.899
Potassium, extractable	mg/kg	139,122	*0.773	*0.509	0.872
Magnesium, extractable	mg/kg	136,681	*0.815	*0.497	0.898
Nitrogen, total	g/kg	99,249	*0.732	*0.197	0.845
Phosphorus, extractable	mg/kg	53,493	*0.486	*0.707	0.654
Sulphur, extractable	mg/kg	37,530	*0.548	*0.384	0.708
Zinc, extractable	mg/kg	39,344	*0.711	*0.375	0.831
Aluminium, extractable	mg/kg	63,551	*0.881	*0.321	0.937
Depth to Bedrock	cm	28,054	0.429	41.3	0.725

**Figure 4:**
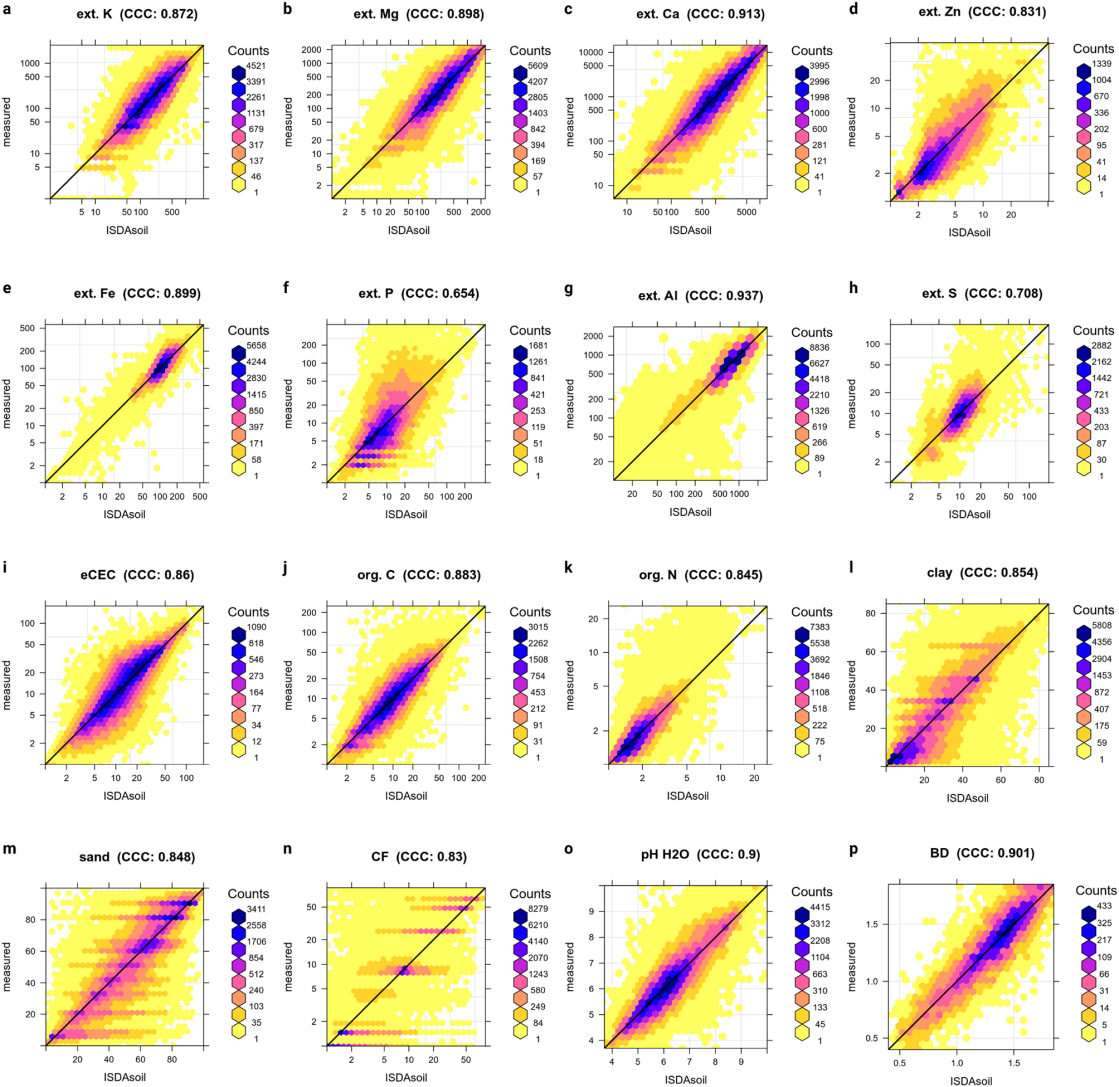
Accuracy assessment plots for all soil nutrients (**a**–**h**) and physical and chemical soil properties (**i**–**p**) based on the final models used for prediction. Accuracy plots derived using fivefold spatial cross-validation. Extractable nutrient concentrations expressed in mg/kg and displayed on a log-scale. *CF* Coarse fragments or stone content, *BD* bulk density.

The spatial Cross-Validation accuracy assessment results (Fig. 4) show that a combination of feature selection and 2-scale modeling results in substantial improvements in prediction performance when compared to the previous work^17^. Improvements in accuracy are especially substantial for ext.-K, ext.-Fe, ext.-P and ext.-Ca, i.e. all variables where 30 m covariates can explain up to 30% of additional variation in the target variables.

The results of stacking various learners indicate that overall Random Forest^23^ seems to perform best in the fivefold Cross Validation, followed by the Lasso and Elastic-Net Regularized Generalized Linear Models (regr.cvglmnet)^24^, while Xgboost^25^ and deepnet^26^ packages only marginally increase accuracy of predictions. The model performance and individual variable importance lists can be also tracked via the https://zenodo.org repositories for iSDAsoil.

In summary, in comparison to our previous work^17^, these accuracy results suggest an average improvement in the R-square value from 0.6 (250 m predictions) to 0.8 (30 m predictions), probably primarily attributable to the addition of higher resolution remote sensing images and Digital Terrain parameters, but also by the adoption of methodological improvements in hyper-parameter tuning, feature selection and ensembling of models using the Super Learner algorithm. Note also that, thanks to the AfSIS project, most of the points used for training have been geo-located with high accuracy (<50 m location error) and this probably also plays an important role in making the fine-resolution imagery useful for predictive mapping.

### Importance of Sentinel-2 data for preparing field-scale nutrient maps

**Figure 5:**
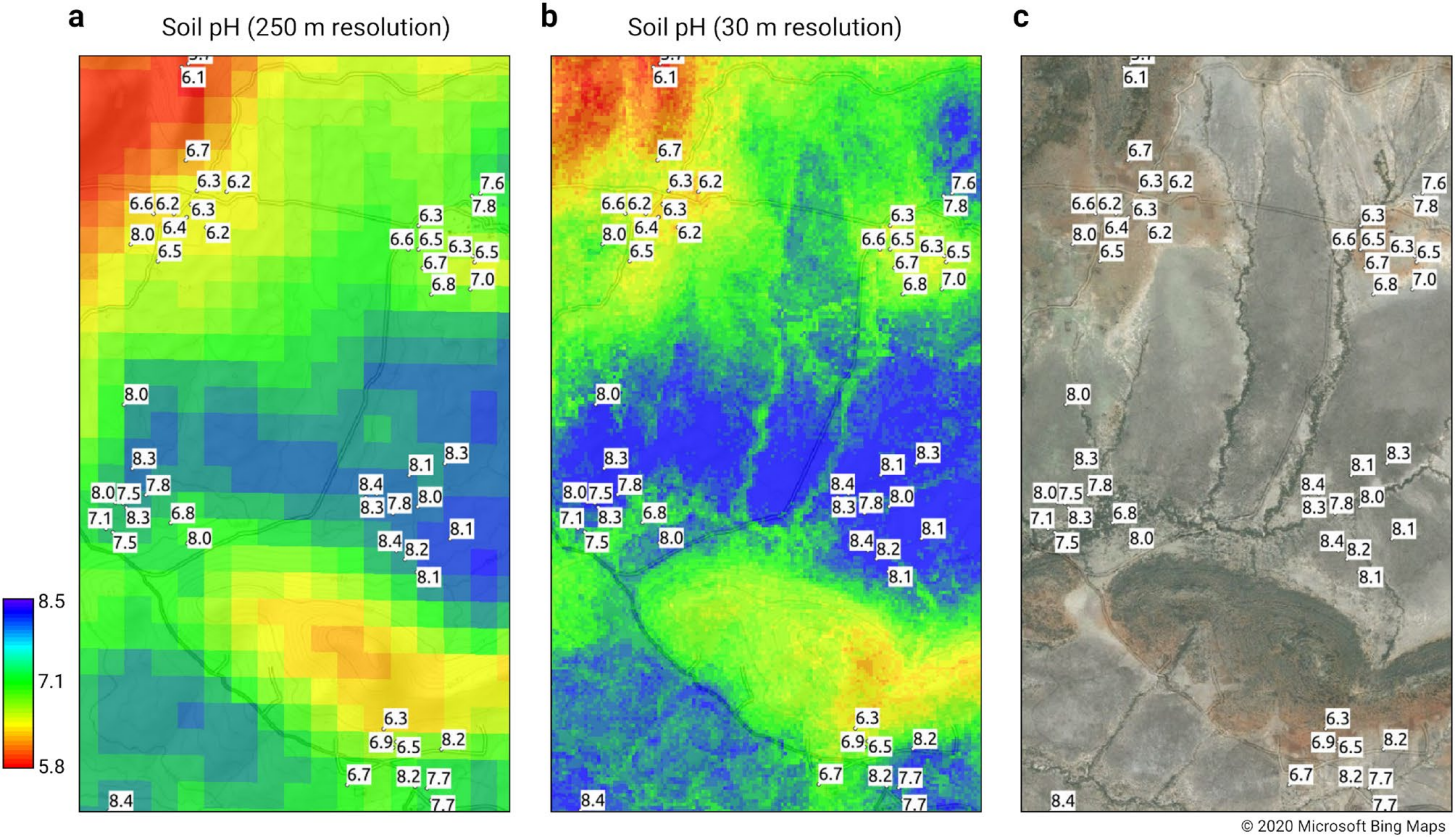
Illustration of differences in spatial detail of predictions for soil pH for top-soil: (**a**) previous predictions at 250 m published in Hengl et al.^17^, (**b**) current predictions at 30 m, which seem to match very well physical patterns seen on the satellite imagery (**c**). Sentinel site area in southern Kenya. Visualizations produced using QGIS v3.10 (https://www.qgis.org/). Satellite image in **c** copyright 2020 Microsoft Bing Maps.

A visual comparison of the new predictions with the previous maps we produced in 2017^17^ indicates that the new predictions better match spatial patterns in the field (Fig. 5). This is especially evident for variables such as soil pH, ext.-Ca, ext.-Mg and sand content, where Sentinel-2 mosaics and AW3D DTM derivatives are identified as being among the most important covariates.

Using a two-scale model was necessary to help us optimize computing when using about 350 covariate layers available at 250 m resolution—mainly climatic/atmospheric images—and some 60 layers—mainly EO data and DTM derivatives—at 30 m. This partitioning helped to speed up processing so that production time remained comparable to e.g. producing the global predictions at 250 m^21^.

### Implications of the main results

The results overall indicate that the additional investment in the preparation of the EO data has proven to be worth the effort. High resolution satellite data has helped us achieve an increase in predictive ability, such that soil properties can now be predicted at 30m resolution, resulting in a highly detailed dataset of roughly 24 billion pixels per layer. Note we expended about 25% of the budget only to process the Sentinel-2 images (about 100 TB of data to derive 25% percentile and interquertile range) to produce the cloud-free Sentinel-2 soil-mapping-ready products for Africa.

Because multiple soil properties were shown to correlate well with continuous EO products such as Sentinel bands (especially B4, B8A, B10, B11 and B12), rainfall images (SM2RAIN), and Land Surface Temperature images (MODIS LST), this opens up possibilities for monitoring changes in soil properties such as soil carbon or soil pH in the future, as Landsat, Sentinel, MODIS and SM2RAIN missions are all expected to continue into the foreseeable future. This could be especially important for monitoring, for example, soil organic carbon changes^27^ and/or soil degradation related to soil erosion, salinization, soil compaction or sealing. It remains to be verified if similar relations between soil organic carbon and 250 m resolution and 30 m resolution EO data is also applicable on other continents.

## Discussion

Over the last decade, the AfSIS project invested considerably in producing a new generation of agronomy data for Africa via AfSIS and related projects. To further extend and derive additional benefit from this primary soil data, we created an agronomy database at a previously unprecedented spatial resolution of 30 m, covering the entire African continent. The newly produced data volumes are substantial: for illustration, one image of Africa at 30 m resolution contains over 24 billion pixels of data (if shifting sand areas such as Sahara are excluded); the average size of a Cloud-Optimized GeoTIFF with internal compression containing predicted values of properties was of the order of 10–20 GiB. By harnessing available Open Access remote sensing data (Sentinel 2, Landsat 7/8), 3D predictive machine learning techniques (ensemble between Random Forest, XGBoost, deepnet, Cubist and GLM-net), and point samples generated by the AfSIS network, as well as a number of other open access soil datasets, we have modeled and produced predictions of 18+ soil variables including: soil texture fractions, soil pH, macronutrients (soil organic carbon, nitrogen, phosphorous, and potassium, magnesium), micronutrients, eCEC and others. The results indicate that the accuracy and spatial detail of previous maps can be considerably improved with average R-square (based on spatial Cross Validation) improving from about 0.6 to values around 0.8.

Our experience is that the Ensemble Multi-scale Predictive Soil Mapping system is a robust, scalable system which basically can be fully automated: from feature selection, model calibration and prediction, to determining quantiles or standard deviation of the prediction error. This is mainly thanks to the flexibility of programming in the mlr package^28^. The results of comparing different learners through fitting of meta-learners indicate that Random Forest^23^ is the overall best-performing learner, but also Lasso and Elastic-Net Regularized Generalized Linear Models and Cubist often perform equally well. Ensembling of multiple learners can be justified for most of the target soil variables.

Mapping soil properties at 30 m and three depths with uncertainties is heavily computational and requires substantial resources. Specifically, derivation of prediction errors can increase production costs considerably, consequently these might need to be estimated using simplified procedures in the future. Also our main rationale for using multiscale models vs one individual model was to try to decrease production costs without experiencing a significant loss of accuracy. The results indicate that the 2-scale EML is especially attractive for reducing computing costs which otherwise would have been about 5–10 times greater if we had tried to downscale ALL of the covariates from 250 to the finest 30 m resolution.

We did not estimate the area of applicability for Machine Learning for Africa per soil variable following the method of Meyer and Pebesma^29^, but our uncertainty maps do clearly reveal areas where the models extrapolate or perform poorly: usually these are densely vegetated tropical areas (Congo basin) or semi-arid parts of Somalia and Sudan. Next-generation soil sampling projects in Africa such as https://www.soils4africa-h2020.eu/ might benefit from using our prediction uncertainty maps to identify new sampling locations e.g. by focusing on the areas that are most difficult to model i.e. that have widest prediction error intervals.

In principle, 2-scale ensembling can be considered to provide a generic framework for predictive soil mapping. It can be extended to consider multiple scales although, for practical purposes, we currently recommend using a minimum of two and a maximum of three scales to avoid increasing the computational complexity unnecessarily. In practice, one could also begin by evaluating multiple scales, then select statistically significant scales, then do ensembling of predictions for only scales identified as significant.

**Figure 6:**
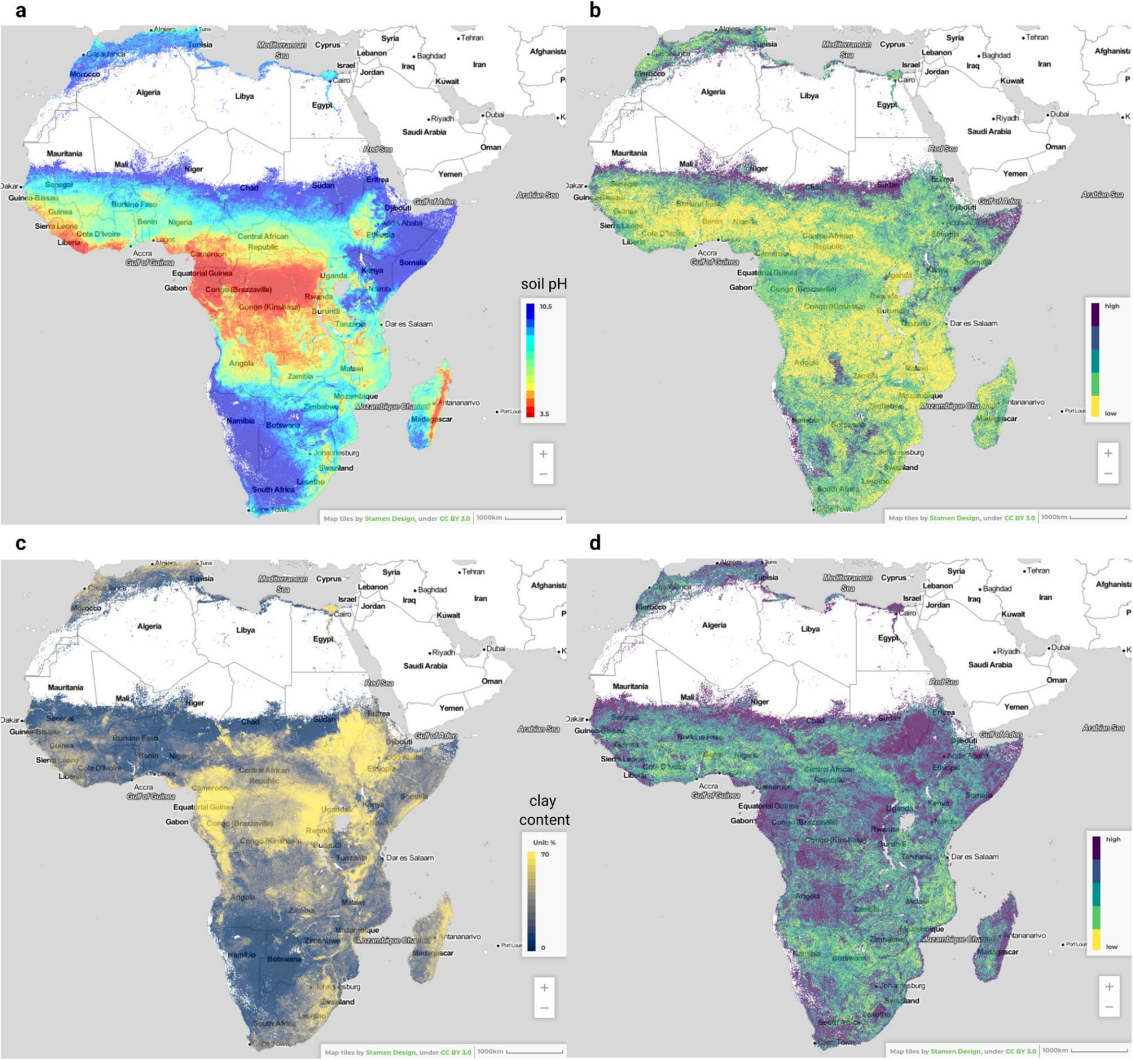
Predictions (left) and prediction uncertainty expressed as 1 s.d. prediction error (right), for soil pH (**a**,**b**) and clay content (**c**,**d**) for the 0–20 cm depth interval. Visualized in the iSDAsoil app: https://isda-africa.com/isdasoil. The prediction error maps for clay content indicate that many areas probably require much more samples than soil pH.

Value of the maps produced for local and/or field based agronomy needs to be evaluated *“on the ground”* and by landowners/farmers. In the first few weeks of testing iSDAsoil app (see Fig. 6), we have already received considerable feedback from experts in Europe and Africa. The main criticisms so far have focused on the low accuracy of the SOC predictions, particularly for peatland areas, on sampling locations over-representing croplands, and on problems with downloading and using these large datasets. The diversity of African soils and the under-representation of specific areas remains a challenge.

We note especially that the following aspects can be considered as requiring more and better training/point data:

- Peatlands in Rwanda, Congo basin and similar remain heavily under-represented, as are all inaccessible tropical jungles or similar remote areas.
- Nutrients P and S and micronutrients Cu, B remain difficult to map using current EO data and or any other type of data available for use in this study. There seems to be no simple solution for this problem and possibly not even $2\times$ more point data for model training than we had here could guarantee success.
- Application (fertilizers), crop history, and similar data from field trials is generally lacking and available only for limited locations e.g. via the Optimizing Fertilizer Recommendations for Africa (OFRA) database^30^.

While there have been criticisms of the absolute accuracy of the iSDAsoil maps, it is important to consider this in the context of real-world applications of the resource, for example in the generation of site-specific fertiliser recommendations. In this case, additional data collection would be required such as land use history, previous fertiliser applications and historic yields. However, we see this resource as a low cost alternative to lab-based soil test that has value in reducing uncertainty around soil properties compared to having no information, which is especially relevant in a smallholder agriculture context^31^.

Our initial predictions are not likely to be correct enough to support informed management at the farm scale immediately. We can, however, propose our initial predictions as being relevant as a starting point, or base, that drives and informs additional new sampling, for each specific parcel of interest. In that sense, our maps provide a uniform and relevant base from which to start building individually relevant predictions for specific parcels of agricultural land. Promotion of first steps for basic improved crop management does not perhaps demand an exceptionally high accuracy of soil data. For example, a good estimate of soil pH can already help to inform which crops may be most suitable to grow/ to not grow or if liming may be needed before any other agrochemicals are used.

Collecting and adding point data from countries such as Democratic Republic of the Congo, Sudan and/or Somalia remains a challenge as there are many serious security challenges for any soil sampling effort. Some recent reports from the Congo have shown that tropical peatlands are probably heavily under-estimated in previous soil maps of Africa^10,32^. Even in relatively safe Tanzania, multiple human casualties occurred during the AfSIS field data collection program, due to unclear land access permission and local militia problems. We anticipate, nevertheless, that a large amount of publicly funded point samples and observations remain unavailable and therefore unused^33^. These could be easily added to modeling and help improve predictions, and the iSDAsoil system has been designed to easily created new versions of the maps based upon additional data.

Another data source that could help improve predictions in the future is the upcoming EU Copernicus Sentinel satellites including the CHIME (Copernicus Hyperspectral Imaging Mission for the Environment), LSTM (Land Surface Temperature Monitoring) and CIMR (Copernicus Imaging Microwave Radiometer)^34^. Here we anticipate that, considering that the MODIS LST images have often proven to be among the most important explanatory variables, the LSTM mission especially could potentially improve the accuracy of soil predictions.

Next-generation soil and/or nutrient modeling in space and time could also probably profit from incorporating EO data that directly measures soil moisture status and Net Primary Productivity (kg $\hbox {ha}^{-1}$ $\hbox {year}^{-1}$). Adding extra training points, adding dynamic EO data products (time-series of images), improving the prediction accuracy for specific soil properties/nutrients will likely result in substantial improvements. For many soil properties (soil texture fractions, depth to bedrock, organic carbon etc) it is difficult to detect meaningful changes in them over time intervals of less than several years (unless some extreme event occurs), nevertheless, soils are a dynamic medium, and mapping and monitoring gradual and abrupt changes, especially in the chemical and biological soil properties will likely become the next frontier of research in Africa.

## Methods

### A 2-scale ensemble machine learning

**Figure 7:**
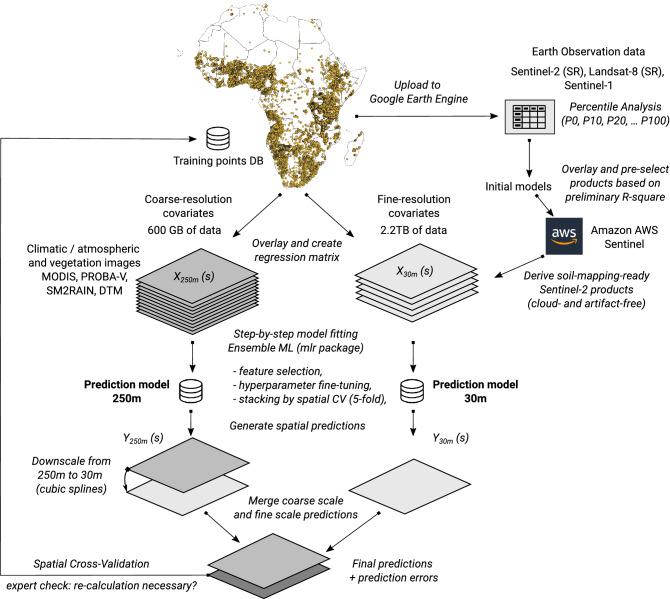
Scheme: a two-scale framework for Predictive Soil Mapping based on Ensemble Machine Learning (as implemented in the mlr and mlr3 frameworks for Machine Learning^28^ and based on the SuperLearner algorithm). This process is applied for a bulk of soil samples, the individual models per soil variable are then fitted using automated fine-tuning, feature selection and stacking. The map is showing distribution of training points used in this work. Part of the training points that are publicly available are available for use from https://gitlab.com/openlandmap/compiled-ess-point-data-sets/.

Predictions of soil nutrients are based on a fully automated and fully optimized 2-scale Ensemble Machine Learning (EML) framework as implemented in the mlr package for Machine Learning (https://mlr.mlr-org.com/). The entire process can be summarized in the following eight steps (Fig. 7):

1. Prepare point data, quality control all values and remove any artifacts or types.
2. Upload to Google Earth Engine, overlay the point data with the key covariates of interest and test fitting random forest or similar to get an initial estimate of relative variable importance and pre-select features of interest.
3. Decide on a final list of all covariates to use in predictions, prepare covariates for predictive modeling—either using Amazon AWS or similar. Quality control all 250 m and 30 m resolution covariates and prepare Analysis-Ready data in a tiling system to speed up overlay and prediction.
4. Run spatial overlay using 250 m and 30 m resolution covariates and generate regression matrices.
5. Fit 250 m and 30 m resolution Ensemble Machine Learning models independently per soil property using spatial blocks of 30–100 km. Run sequentially: model fine-tuning, feature selection and stacking. Generate summary accuracy assessment, variable importance, and revise if necessary.
6. Predict 250 m and 30 m resolution tiles independently using the optimized models. Downscale the 250 m predictions to 30 m resolution using Cubicsplines (GDAL).
7. Combine predictions using Eq. (3) and generate pooled variance/s.d. using Eq. (4).
8. Generate all final predictions as Cloud-Optimized GeoTIFFs. Upload to the server and share through API/Geoserver.

For the majority of soil properties, excluding depth to bedrock, we also use soil depth as one of the covariates so that the final models for the two scales are in the form^5^:

1 $$\begin{aligned} y(\phi ,\theta ,d) = d + x_1 (\phi ,\theta ) + x_2 (\phi ,\theta ) + \cdots + X_p (\phi ,\theta ) \end{aligned}$$

where *y* is the target variable, *d* is the soil sampling depth, $\phi \theta$ are geographical coordinates (northing and easting), and $X_p$ are the covariates. Adding soil depth as a covariate allows for directly producing 3D predictions^35^, which is our preferred approach as prediction can be then produced at any depth within the standard depth interval (e.g. 0–50 cm).

### Ensemble machine learning

Ensembles are predictive models that combine predictions from two or more learners^36^. We implement ensembling within the mlr package by fitting a *‘meta-learner’* i.e. a learner that combines all individual learners. mlr has extensive functionality, especially for model *‘stacking’* i.e. to generate ensemble predictions, and also incorporates spatial Cross-Validation^37^. It also provides wrapper functions to automate hyper-parameter fine-tuning and feature selection, which can all be combined into fully-automated functions to fit and optimize models and produce predictions. Parallelisation can be initiated by using the parallelMap package, which automatically determines available resources and cleans-up all temporary sessions^38^.

For stacking multiple base learners we use the SuperLearner method^39^, which is the most computational method but allows for an independent assessment of all individual learners through *k*-fold cross validation with refitting. To speed up computing we typically use a linear model (predict.lm) as the meta-learner, so that in fact the final formula to derive the final ensemble prediction can be directly interpreted by printing the model summary.

The predictions in the Ensemble models described in Fig. 7 are in principle based on using the following five Machine Learning libraries common for many soil mapping projects^5^.

1. Ranger: fully scalable implementation of Random Forest^23^.
2. XGboost: extreme gradient boosting^40^.
3. Deepnet: the Open Source implementation of deep learning^26^.
4. Cubist: the Open Source implementation of Cubist regression trees^41^.
5. Glmnet: GLM with Lasso or Elasticnet Regularization^24^.

These Open source libraries, with the exception of the Cubist, are available through a variety of programming environments including R, Python and also as standalone C++ libraries.

### Merging coarse and fine-scale predictions

**Figure 8:**
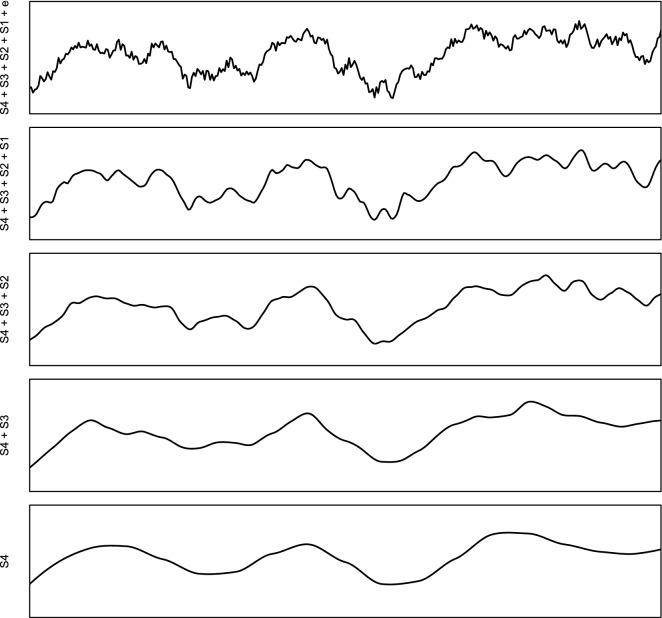
Decomposition of a signal of spatial variation into four components plus noise. Based on McBratney^42^. See also Fig. 13 in Hengl et al.^21^.

The idea of modeling soil spatial variation at different scales can be traced back to the work of McBratney^42^. In a multiscale model, soil variation can be considered a composite signal (Fig. 8):

2 $$\begin{aligned} y({\mathbf{s}}_{\mathtt {B}}) = S_4({\mathbf{s}}_{\mathtt {B}}) + S_3({\mathbf{s}}_{\mathtt {B}}) + S_2({\mathbf{s}}_{\mathtt {B}}) + S_1({\mathbf{s}}_{\mathtt {B}}) + \varepsilon \end{aligned}$$

where $S_4$ is the value of the target variable estimated at the coarsest scale, $S_3$, $S_2$ and $S_1$ are the higher order components, ${\mathbf{s}}_{\mathtt {B}}$ is the location or block of land, and $\varepsilon$ is the residual soil variation i.e. pure noise.

In this work we used a somewhat simplified version of Eq. (2) with only two scale-components: coarse ($S_2$; 250 m) and fine ($S_1$; 30 m). We produce the coarse-scale and fine-scale predictions independently, then merge using a weighted average^43^:

3 $$\begin{aligned} {\hat{y}}({\mathbf{s}}_{\mathtt {B}}) = \frac{\sum _{i=1}^{2}{ w_i \cdot S_i({\mathbf{s}}_{\mathtt {B}})}}{\sum _{i=1}^{2}{ w_i }}, \; \; w_i = \frac{1}{\sigma _{i,\mathrm{CV}}^2} \end{aligned}$$

where ${\hat{y}}({\mathbf{s}}_{\mathtt {B}})$ is the ensemble prediction, $w_i$ is the model weight and $\sigma _{i,\mathrm{CV}}^2$ is the model squared prediction error obtained using cross-validation. This is an example of Ensemble Models fitted for coarse-scale model for soil pH:
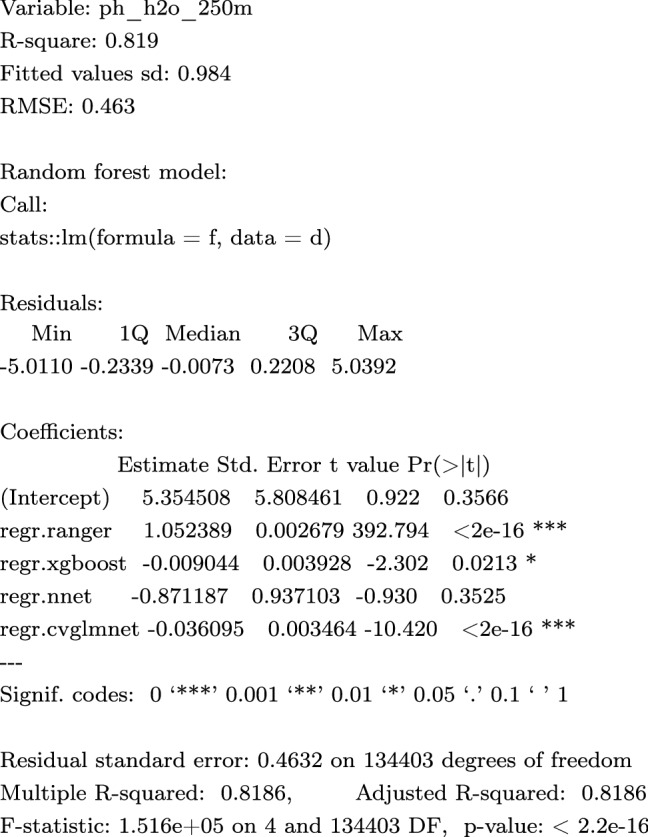


and the fine-scale model for soil pH:
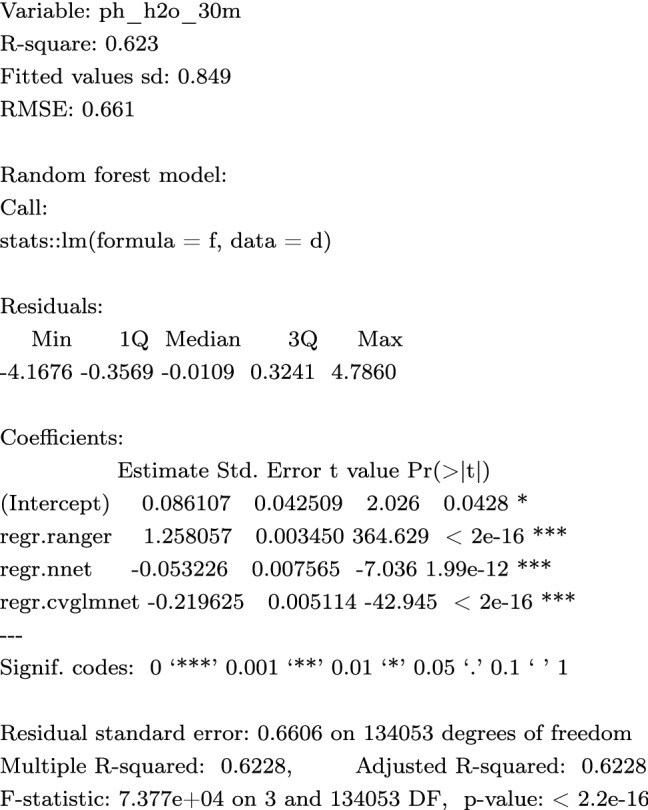


Note that in this case the coarse-scale model is somewhat more accurate with $\mathrm {RMSE}=0.463$, while the 30 m covariates achieve at best $\mathrm {RMSE}=0.661$, hence the weights for 250 m model are about 2$\times$ higher than for the 30 m resolution models. A step-by-step procedure explaining in detail how the 2-scale predictions are derived and merged is available at https://gitlab.com/openlandmap/spatial-predictions-using-eml. An R package landmap^44^ that implements the procedure in a few lines of code is also available.

### Transformation of log-normally distributed nutrients and properties

For the majority of log-normal distributed (right-skewed) variables we model and predict the ln-transformed values ($\log _e(x+1)$), then provide back-transformed predictions ($e^{x}-1$) to users via iSDAsoil. Note that also pH is a log-transformed variable of the hydrogen ion concentrations.

Although ln-transformation is not required for non-linear models such as Random Forest or Gradient Boosting, we decided to apply it to give proportionally higher weights to lower values. This is, in principle, a biased decision by us the modelers as our interest is in improving predictions of critical values for agriculture i.e. producing maps of nutrient deficiencies and similar (hence focus on smaller values). If the objective of mapping was to produce soil organic carbon of peatlands or similar, then the ln-transformation could have decreased the overall accuracy, although with Machine Learning models sometimes it is impossible to predict effects as they are highly non-linear.

### Derivation of prediction errors

We also provide per-pixel uncertainty in terms of prediction errors or prediction intervals (e.g. 50%, 68% and/or 90% probability intervals)^45^. Because stacking of learners is based on repeated resampling, the prediction errors (per pixel) can be determined using either:

1. Quantile Regression Random Forest^46^, in our case by using the 4–5 base learners,
2. Simplified procedure using Bootstraping, then deriving prediction errors as standard deviation from multiple independently fitted learners^1^.

Both are non-parametric techniques and the prediction errors do not require any assumptions or initial parameters, but come at a cost of extra computing. By default, we provide prediction errors with a probability of 67%, which is the 1 standard deviation upper and lower prediction interval. Prediction errors indicate extrapolation areas and should help users minimize risks of taking decisions.

For derivation of prediction interval via either Quantile Regression RF or bootstrapping, it is important to note that the individual learners must be derived using randomized subsets of data (e.g. fivefold) which are spatially separated using block Cross-Validation or similar, otherwise the results might be over-optimistic and prediction errors too narrow.

**Figure 9:**
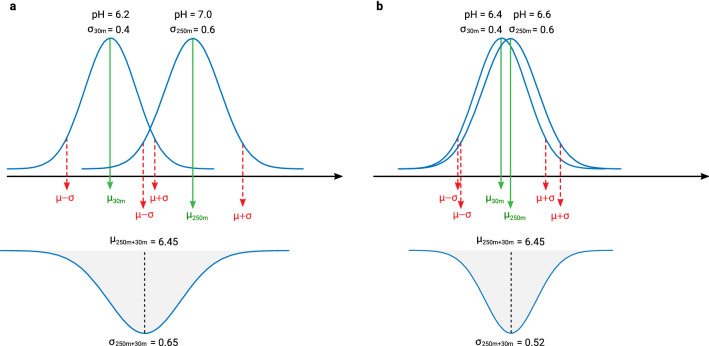
Schematic example of the derivation of a pooled variance ($\sigma _{\mathtt {250m+30m}}$) using the 250 m and 30 m predictions and predictions errors with (**a**) larger and (**b**) smaller differences in independent predictions.

Further, the pooled variance (${\hat{\sigma }}_E$) from the two independent models (250 m and 100 m scales in Fig. 7) can be derived using^47^:

4 $$\begin{aligned} {\hat{\sigma }}_E = \sqrt{\sum _{j=1}^{s}{w_j \cdot (\hat{\sigma }_j^2+{\hat{\mu }}_j^2 )} - \left( \sum _{j=1}^{s}{w_j \cdot {\hat{\mu }}_j} \right) ^2 }, \; \; \sum _{j=1}^{s}{w_j} = 1 \end{aligned}$$

where $\sigma _j^2$ is the prediction error for the independent components, ${\hat{\mu }}_j$ is the predicted value, and *w* are the weights per predicted component (need to sum up to 1). If the two independent models (250 m and 30 m) produce very similar predictions so that ${\hat{\mu }}_{\mathtt {250}} \approx {\hat{\mu }}_{\mathtt {30}}$, then the pooled variance approaches the geometric mean of the two variances; if the independent predictions are different (${\hat{\mu }}_{\mathtt {250}} - {\hat{\mu }}_{\mathtt {30}} > 0$) than the pooled variances increase proportionally to this additional difference (Fig. 9).

### Accuracy assessment of final maps

We report overall average accuracy in Table 1 and Fig. 4 using spatial fivefold Cross-Validation with model refitting^1,48^. For each variable we then compute the following three metrics: (1) Root Mean Square Error, (2) R-square from the meta-learner, and (3) Concordance Correlation Coefficient (Fig. 4), which is derived using^49^:

5 $$\begin{aligned} \rho _c = \frac{2 \cdot \rho \cdot \sigma _{{{\hat{y}}}} \cdot \sigma _y }{ \sigma _{{{\hat{y}}}}^2 + \sigma _y^2 + (\mu _{{{\hat{y}}}} - \mu _y)^2} \end{aligned}$$

where ${{\hat{y}}}$ are the predicted values and *y* are actual values at cross-validation points, $\mu _{{{\hat{y}}}}$ and $\mu _y$ are predicted and observed means and $\rho$ is the correlation coefficient between predicted and observed values. CCC is the most appropriate performance criteria when it comes to measuring agreement between predictions and observations.

For Cross-validation we use the spatial tile ID produced in the equal-area projection system for Africa (Lambert Azimuthal EPSG:42106) as the blocking parameter in the training function in mlr. This ensures that points falling in close proximity (<30 km) are either used for training or for validation, which ultimately provides a more objective measure of accuracy for the whole of the continent^48^.

### Training points

For model training we used a compilation of existing data previously produced by the AfSIS project and/or other publicly available soils data (Fig. 7). The important training point datasets include:

- AfSIS I and II soil samples for Tanzania, Uganda, Nigeria, Ghana: ca. 40,000 sampling locations, based upon spectral and wet chemistry data (available from: https://registry.opendata.aws/afsis/). AfSIS I dataset was prepared by ICRAF using a systematic sampling procedure^50,51^,
- ISRIC Africa Soil Profile Database: ca. 13,000 legacy profiles collected across Africa and collated by ISRIC as part of the AfSIS project^13^,
- LandPKS: ca. 12,000 soil profile observations, crowd sourced and collected via the LandPKS mobile app^52^,
- IFDC: ca. 9,000 soil sampling locations across Ghana, Uganda, Rwanda and Burundi collected from various projects,
- AfricaRice and TAMASA: ca. 3,000 soil sampling locations across Africa generated from field trials/surveys by AfricaRice^53^ and Taking Maize Agronomy to Scale in Africa (TAMASA).

In total this consists of more than 100,000 soil sites (unique locations) from over 20 datasets, measured using wet chemistry and dry spectroscopy^54^. The final training dataset includes between ca. 30,000–150,000 cleaned and standardized training samples depending on the variable (see Table 1).

iSDA was supported by ICRAF to leverage their extensive spectral calibration libraries in order to generate accurate and inexpensive soil property predictions from spectral data^55^. Analytical methods used for soil variables included the laser diffraction method for clay and sand fractions, the Mehlich3 extraction for extractable nutrients, pH was determined in 1:2 deionised water, eCEC was determined with the Cobalthexamine method and thermal oxidation and subtraction of inorganic carbon was used for soil organic carbon. We paid special attention to filtering out artifacts in the input points, filling in gaps in the point data, and leveraging expert agronomy rules. A full harmonization of different laboratory methods used in different data sets was not conducted but we ensured that only data from comparable methods with a similar range of results were used. Different extraction or analysis methods that can easily depart from each other by factors of 2–10. For example, different ex-P methods. For this reasons we have rather opted to splitting some variables into groups and/or omitting measurements that are incompatible with the majority of measurements.

The training points from the LandPKS project are, in fact, non-laboratory variables i.e. quick estimates of texture by hand. To convert the values from e.g. clay-loam texture class to clay, silt and sand fractions we use the texture triangle centroids^5^ e.g. the class *“clay”* is converted to 20% sand, 18% silt and 63% clay and similar. The results of converting the values are thus visible as groupings in the observed data in the accuracy plots (Fig. 4) for sand, silt, clay and coarse fragments (CF)/stone content.

Part of the training datasets used for model building, and import and standardization rules are listed via a public repository at https://gitlab.com/openlandmap/compiled-ess-point-data-sets/. For an up-to-date overview of training point datasets used, please refer to https://isda-africa.com/isdasoil.

### Covariate layers

We use an extensive stack of covariates that includes up-to-date MODIS, PROBA-V, cloud free Sentinel 2 mosaics, Landsat data, digital terrain parameters and climactic variables. The 250 m resolution covariates include (see Supplementary material for a complete list with file names):

- Digital Terrain Model DTM-derived surfaces—slope, profile curvature, Multiresolution Index of Valley Bottom Flatness (VBF), deviation from Mean Value, valley depth, negative and positive Topographic Openness and SAGA Wetness Index—all based on the MERIT-DEM^56^ and computed using the SAGA GIS^57^ using varying spatial resolutions (250 m, 1 km, 2 km);
- CHELSA Bioclimatic images^58^ downloaded from https://chelsa-climate.org/bioclim/,
- SM2RAIN monthly mean and standard deviation images^59^ available for download from https://doi.org/10.5281/zenodo.1435912;
- Long-term averaged mean monthly surface reflectances for MODIS bands 4 (NIR) and 7 (MIR) at 500 m resolution. Derived using a stack of MOD09A1 images;
- Long-term averaged monthly mean and standard deviation of the MODIS land surface temperature (daytime and nighttime). Derived using a stack of MOD11A2 LST images^60^ which can be downloaded from https://doi.org/10.5281/zenodo.1420114;
- MODIS Cloud fraction monthly images^61^ obtained from http://www.earthenv.org/cloud;
- Solar direct and diffuse irradiation images obtained from https://globalsolaratlas.info/download;
- Fraction of Absorbed Photosynthetically Active Radiation (FAPAR) at 250 m monthly for period 2014–2017^62^ based on COPERNICUS land products that can be downloaded https://doi.org/10.5281/zenodo.1450336;
- Long-term Flood hazard map for a 500-year return period^63^;
- USGS Africa Surface Lithology map at 250 m resolution^22^.

CHELSA bioclimatic images include: (Bio1) annual mean temperature, (Bio2) mean diurnal temperature range, (Bio3) isothermality (day-to-night temperature oscillations relative to the summer-to-winter oscillations), (Bio4) temperature seasonality (standard deviation of monthly temperature averages), (Bio5) maximum temperature of warmest month, (Bio6) minimum temperature of coldest month, (Bio7) temperature annual range, (Bio10) mean temperature of warmest quarter, (Bio11) mean temperature of coldest quarter, (Bio12) annual precipitation amount, (Bio13) precipitation of wettest month, (Bio14) precipitation of driest month, (Bio16) precipitation of wettest quarter, (Bio17) precipitation of driest quarter. All layers were processed in the native resolution then, if necessary, downscaled to the same grid using bicubic splines resampling in GDAL^64^. The USGS Africa Surface Lithology map units were converted to indicators with some units being excluded for having too few ($<5$) training points.

The 30 m resolution covariates include:

- Digital Terrain Model DTM-derived surfaces derived using the AW3D digital elevation model^65^ downloaded from https://www.eorc.jaxa.jp/ALOS/en/aw3d30/data/, and combined with the NASA DEM 30 m resolution product downloaded from https://lpdaac.usgs.gov/products/nasadem_hgtv001/;
- Sentinel-2 L2A cloud-free mosaics of bands B02, B04, B8A, B09, B10, B11 and B12 derived as 25%, 75% percentiles and inter-quantile ranges (IQR) processed via the AWS Open Registry (https://registry.opendata.aws/sentinel-2/). Mosaics are computed for two seasons for years 2018 and 2019 (Fig. 7);
- Existing Landsat cloud-free products with NIR and SWIR images based on the Global Forest Change project^20^ and downloaded from https://earthenginepartners.appspot.com/science-2013-global-forest;
- Global Surface Water long-term probability images based on Pekel et al.^66^ and downloaded from https://global-surface-water.appspot.com.

We have pre-selected the 30 m resolution EO data for mapping soil nutrients over Africa, to still stay within the project budget by using the following procedure (Fig. 7):

1. Upload points to the Google Earth Engine^67^, overlay and fit initial Random Forest models to identify and prioritize the most important bands;
2. Processed prioritized bands using Amazon AWS; this is still tens of Terrabytes of Sentinel data, but considerably less than if all bands would have been selected and processed;
3. Produce cloud free mosaics for the period 2018–2019 using Amazon AWS; download the final product as Cloud-Optimised GeoTIFFs;
4. Run spatial overlay, model fitting and prediction in a local system using Solid State Disk drive and servers with a lot of RAM.

We refer to this as *“the hybrid Cloud-based 2–step variable selection procedure”* (Fig. 7). With it we combine the power of Google Earth Engine with our own computing infrastructure to achieve customized processing.

The Sentinel-2 cloud-free images were produced using the Scene Classification Mask (SCL band) for two seasons (S1 = months 1, 2, 3, 7, 8, 9, and S2 = 4, 5, 6, 10, 11, 12) combined through 2018 and 2019 year, to minimize number of pixels with clouds. We processed a total of 852,738 Sentinel-2 L2A scenes, or about 200TB of raw data. Scenes were processed by splitting the African continent into 8721 tiles (2000$\times$2000 pixels or 60$\times$60 km). For processing these large volumes of data we used the AWS EC2 Spot Instances (Auto Scaling Groups) with 3GB of RAM per vCPU and few TB of ephemeral (temporary) storage for satellite images. The total processing time to produce all Sentinel-2 products took ca. 100,000 h of computing. Average time required to produce one cloud-free tile per tile/band/season ranged between 90 min for B02, B04 and 50 min for B8A, B09, B11, B12.

For predictive mapping we use a fully-optimized High Performance Computing system (3$\times$ Scan 3XS servers) using the Intel Xeon Gold chip-set with 40 CPU cores/80 treads.

## Supplementary Information

Supplementary Information.

## Data Availability

The iSDAsoil dataset is available under the Creative Commons Attribution 4.0 (CC-BY) International license and can be accessed via https://isda-africa.com/isdasoil. Cloud-optimized GeoTIFFs can be downloaded via https://zenodo.org/search?q=iSDAsoil.
