# Supplementary material for: African soil properties and nutrients mapped at 30 m spatial resolution using two-scale ensemble machine learning
Source: Sci Rep. 2021 Mar 17;11:6130. doi: 10.1038/s41598-021-85639-y (PMC7969779; doi:10.1038/s41598-021-85639-y)
Supplement: Supplementary file 1 — Supplementary Information. [file 41598_2021_85639_MOESM1_ESM.pdf]

African Soil Properties and Nutrients Mapped at 30–m Spatial Resolution using Two-scale Ensemble Machine Learning

Tomislav Hengl[1,2,*], Matthew A. E. Miller[3], Josip Krizan[4], Keith D. Shepherd[5], Andrew Sila[5], Milan Kilibarda[6], Ognjen Antonijevic[6], Luka Glusica[7], Achim Dobermann[8], Stephan M. Haefele[9], Steve P. McGrath[9], Gifty E. Acquah[9], Jamie Collinson[3], Leandro Parente[2], Mohammadreza Sheykhmousa[2], Kazuki Saito[10], Jean-Martial Johnson[10], Jordan Chamberlin[11], Francis B. T. Silatsa[12], Martin Yemefack[12], John Wendt[13], Robert A. MacMillan[2], Ichsani Wheeler[1,2], and Jonathan Crouch[3]

[1]EnvirometriX Ltd, Wageningen, the Netherlands
[2]OpenGeoHub Foundation, Wageningen, the Netherlands
[3]Innovative Solutions for Decision Agriculture Ltd (iSDA), Harpenden, United Kingdom
[4]MultiOne Ltd, Zagreb, Croatia
[5]World Agroforestry (ICRAF), Nairobi, Kenya
[6]Department of Geodesy and Geoinformatics, Faculty of Civil Engineering, University of Belgrade, Belgrade, Serbia
[7]GILAB Ltd, Belgrade, Serbia
[8]International Fertilizer Association (IFA), Paris, France
[9]Rothamsted Research, Harpenden, United Kingdom
[10]Africa Rice Center (AfricaRice), Bouaké, Côte d'Ivoire
[11]International Maize and Wheat Improvement Centre (CIMMYT), Nairobi, Kenya
[12]Sustainable Tropical Solutions (STS) Sarl, Yaoundéc, Cameroon
[13]International Fertilizer Development Center (IFDC), Muscle Shoals, AL, USA

* Corresponding author e-mail: tom.hengl@envirometrix.net

Supplement Table 1: List of soil and auxiliary variables available from iSDAsoil. The DOI i.e. the data download link is indicated in the first column.

**Spatial layer**	**Extraction / measurement information**	**Unit**	**Available depths (cm)**	**Spatial resolution (m)**	**Theme**	**Uncertainty available?**	**N training samples**	**Model Accuracy (R-square)**	**RMSE**
Land Cover (2015 - 2019) https://doi.org/10.5281/zenodo.3606295	N/A	%	0	100	Agronomy information	No	N/A	N/A	N/A
Cropland (2015 - 2019) https://doi.org/10.5281/zenodo.3606295	N/A	%	0	100	Agronomy information	No	N/A	N/A	N/A
Slope Angle http://isda-africa.com/isdasoil	N/A	degree	0	30	Physical soil properties and landscape	No	N/A	N/A	N/A
Fertility Capability Classification http://isda-africa.com/isdasoil	N/A	None	0–50	30	Agronomy information	No	N/A	N/A	N/A
USDA Texture Class https://doi.org/10.5281/zenodo.4094615	USDA 12 class system	None	0–20, 0–50	30	Physical soil properties and landscape	No	N/A	N/A	N/A
Clay content https://doi.org/10.5281/zenodo.4085159	Various	%	0–20, 0–50	30	Physical soil properties and landscape	Yes	122,269	0.746	9.6
Bulk density, <2mm fraction https://doi.org/10.5281/zenodo.4087904	Various	g/cc	0–20, 0–50	30	Physical soil properties and landscape	Yes	13,565	0.819	126
Aluminium, extractable https://doi.org/10.5281/zenodo.4087935	Mehlich-3	ppm	0–20, 0–50	30	Soil nutrients	Yes	63,551	0.881*	0.321*
Carbon, total https://doi.org/10.5281/zenodo.4088063	Various	g/kg	0–20, 0–50	30	Soil properties	Yes	50,140	0.794*	0.291*
Calcium, extractable https://doi.org/10.5281/zenodo.4087971	Mehlich-3	ppm	0–20, 0–50	30	Soil nutrients	Yes	144,593	0.840*	0.543*
Effective Cation Exchange Capacity https://doi.org/10.5281/zenodo.4088112	Mehlich-3	cmol(+)/kg	0–20, 0–50	30	Chemical soil properties	Yes	66,380	0.754*	0.417*
Iron, extractable https://doi.org/10.5281/zenodo.4088215	Mehlich-3	ppm	0–20, 0–50	30	Soil nutrients	Yes	57,526	0.817*	0.235*
Potassium, extractable https://doi.org/10.5281/zenodo.4090297	Mehlich-3	ppm	0–20, 0–50	30	Soil nutrients	Yes	139,122	0.773*	0.509*

Magnesium, extractable https://doi.org/10.5281/zenodo.4090373	Mehlich-3	ppm	0–20, 0–50	30	Soil nutrients	Yes	136,681	0.815*	0.497*
Nitrogen, total https://doi.org/10.5281/zenodo.4090385	Measured by Total combustion	g/kg	0–20, 0–50	30	Chemical soil properties	Yes	99,249	0.732*	0.197*
Carbon, organic https://doi.org/10.5281/zenodo.4090926	Various	g/kg	0–20, 0–50	30	Chemical soil properties	Yes	122,457	0.791*	0.369*
Phosphorus, extractable https://doi.org/10.5281/zenodo.4090980	Mehlich-3	ppm	0–20, 0–50	30	Soil nutrients	Yes	53,493	0.486*	0.707*
Sulphur, extractable https://doi.org/10.5281/zenodo.4091141	Mehlich-3	ppm	0–20, 0–50	30	Soil nutrients	Yes	37,530	0.548*	0.384*
Zinc, extractable https://doi.org/10.5281/zenodo.4091170	Mehlich-3	ppm	0–20, 0–50	30	Soil nutrients	Yes	39,344	0.711*	0.375*
pH in H2O https://doi.org/10.5281/zenodo.4073236	1:1 Soil-Water Suspension	–	0–20, 0–50	30	Chemical soil properties	Yes	133,378	0.818	0.459
Sand content https://doi.org/10.5281/zenodo.4094606	Various	%	0–20, 0–50	30	Physical soil properties and landscape	Yes	122,261	0.736	13.7
Silt content https://doi.org/10.5281/zenodo.4094609	Various	%	0–20, 0–50	30	Physical soil properties and landscape	Yes	122,223	0.64	8.92
Stone content https://doi.org/10.5281/zenodo.4091153	Various	%	0–20, 0–50	30	Physical soil properties and landscape	Yes	92,785	0.709*	0.803*
Depth to Bedrock https://doi.org/10.5281/zenodo.4094615	Estimated from soil profile data	cm	0–200	30	Physical soil properties and landscape	Yes	28,054	0.429	41.3

Supplement Table 2: Complete list and description of all covariate layers (file names) used for model fitting.

File_name	Description	Source_URL	Spatial_resolution_native	Spatial_resolution_PSM
clm_precipitation_sm2rain.*_m_1km_s0..0cm_2007..2018_v0.2.tif	Precipitation monthly in mm. Based on the SM2RAIN-ASCAT 2007-2018, GPM (IMERG) 2014–2018 and WorldClim v2, CHELSA rainfall monthly images.	https://doi.org/10.5281/zenodo.2591215	1000	250
clm_precipitation_sm2rain.*_sd_1km_s0..0cm_2007..2018_v0.2.tif	Precipitation monthly sd in mm. Based on the SM2RAIN-ASCAT 2007-2018, GPM (IMERG) 2014–2018 and WorldClim v2, CHELSA rainfall monthly images.	https://doi.org/10.5281/zenodo.2591215	10000	250
clm_*.irradiation_solar.atlas.kwhm2.100_m_1km_s0..0cm_2016_v1.tif	Global horizontal and Diffuse horizontal irradiation [kWh/m2] based on Solargis	https://globalsolaratlas.info/downloads/world	1000	250
dtm_*_usgs.ecotapestry.*_p_250m_s0..0cm_2014_v1.0.tif	USGS Global Ecophysiography landform classification and lithological map	http://rmgsc.cr.usgs.gov/outgoing/ecosystems/Global/	250	250
dtm_floodmap.500y_jrc.hazardmapping_m_250m_s0..0cm_1500..2016_v1.0.tif	Flood hazard map for Europe, 500-year return period	https://data.europa.eu/euodp/en/data/dataset/jrc-floods-floodmapeu_rp500y-tif	1000	250
dtm_water.table.depth_deltares_m_1km_b0..150m_2016_v1.0.tif	Simulated water table depth in metres		1000	250
clm_snow.prob_esacci.*_p_1km_s0..0cm_2000..2016_v1.0.tif	Snow probability monthly. Based on the CCI Land Cover dataset / MOD10A2 product at 500 m for the period 2000–2012.	https://www.esa-landcover-cci.org/?q=node/161	250	250
clm_water.vapor_nasa.eo.*_m_1km_s0..0cm_2000..2017_v1.0.tif	NASA's monthly MODIS Precipitable Water Vapor images (MYDAL2_M_SKY_WV)	http://neo.sci.gsfc.nasa.gov/	10000	250
clm_wind.speed_terraclimate.*_m_5km_s0..0cm_1998..2018_v1.tif	Wind speed based on TerraClimate	https://climate.northwestknowledge.net/TERRACLIMATE/index_directDownloads.php	5000	250
dtm_*_merit.dem_m_250m_s0..0cm_2018_v1.0.tif	Global high-resolution geomorphometry layers - slope, eastness, northness, roughness scale, TWI, MrVBF	http://www.spatial-ecology.net/dokuwiki/doku.php?id=topovar90m	100	250
dtm_*_merit.hydro_m_250m_s0..0cm_2018_v1.0.tif	MERIT Hydro Upstream Drainage Area and Height Above Nearest Drainage	http://hydro.iis.u-tokyo.ac.jp/~yamadai/MERIT_Hydro/	100	250
clm_cloud.fraction_earthenv.modis.*_m_1km_s0..0cm_2000..2015_v1.0.tif	MODIS Cloud fraction monthly images	http://www.earthenv.org/cloud	1000	250
lcv_wetlands.cw_upmc.*_p_250m_b0..200cm_2010..2015_v1.0.tif	Multi-source global wetland maps combining surface water imagery	https://doi.org/10.1594/PANGAEA.892657	1000	250

	and groundwater constraints			
clm_lst_mod11a2.*.day_m_1km_s0..0cm_2000..2017_v1.0.tif	MODIS MOD11A2 Land Surface Temperature daytime median value.	https://lpdaac.usgs.gov/dataset_discovery/modis/modis_products_table/mod11a2_v006	1000	250
clm_lst_mod11a2.*.day_sd_1km_s0..0cm_2000..2017_v1.0.tif	MODIS MOD11A2 Land Surface Temperature daytime sd value.	https://lpdaac.usgs.gov/dataset_discovery/modis/modis_products_table/mod11a2_v007	1000	250
clm_lst_mod11a2.*.daynight_m_1km_s0..0cm_2000..2017_v1.0.tif	MODIS MOD11A2 Land Surface Temperature day-night difference.	https://lpdaac.usgs.gov/dataset_discovery/modis/modis_products_table/mod11a2_v008	1000	250
lcv_surf.refl.b01_mod09a1.pc*_m_500m_s0..0cm_2001_v1.0.tif	MODIS MOD09A1 Surface Reflectance 8-Day L3 Global 500 m long-term B01 PCs	https://lpdaac.usgs.gov/products/mod09a1v006/	500	250
lcv_surf.refl.b02_mod09a1.pc*_m_500m_s0..0cm_2001_v1.0.tif	MODIS MOD09A1 Surface Reflectance 8-Day L3 Global 500 m long-term B02 PCs	https://lpdaac.usgs.gov/products/mod09a1v006/	500	250
lcv_surf.refl.b05_mod09a1.pc*_m_500m_s0..0cm_2001_v1.0.tif	MODIS MOD09A1 Surface Reflectance 8-Day L3 Global 500 m long-term B05 PCs	https://lpdaac.usgs.gov/products/mod09a1v006/	500	250
lcv_surf.refl.b06_mod09a1.pc*_m_500m_s0..0cm_2001_v1.0.tif	MODIS MOD09A1 Surface Reflectance 8-Day L3 Global 500 m long-term B06 PCs	https://lpdaac.usgs.gov/products/mod09a1v006/	500	250
lcv_surf.refl.b07_mod09a1.pc*_m_500m_s0..0cm_2001_v1.0.tif	MODIS MOD09A1 Surface Reflectance 8-Day L3 Global 500 m long-term B07 PCs	https://lpdaac.usgs.gov/products/mod09a1v006/	500	250
lcv_mangroves_unep.wcmc_p_500m_s0..0cm_2011_v1.0.tif	Status and distribution of mangrove forests of the world using earth observation satellite data (version 1.3, updated by UNEP-WCMC)	https://data.unep-wcmc.org/datasets/4	500	250
veg_fapar_proba.v.*_d_250m_s0..0cm_2014..2017_v1.0.tif	FAPAR median monthly value 2014–2017 based on the Copernicus PROB-V FAPAR product.	https://land.copernicus.eu/global/products/fapar	250	250
veg_fapar_proba.v.*_r_250m_s0..0cm_2014..2017_v1.0.tif	FAPAR IQR monthly value 2014–2017 based on the Copernicus PROB-V FAPAR product.	https://land.copernicus.eu/global/products/fapar	250	250
veg_fapar_proba.v.annual_d_250m_s0..0cm_2014..2017_v1.0.tif	FAPAR median annual value 2014–2017 based on the Copernicus PROB-V FAPAR product.	https://land.copernicus.eu/global/products/fapar	250	250
lcv_b02_sentinel.s2l2a_d_30m_s0..0cm_2019.s1_v0.1.tif	Sentinel 2 Band 2 – Blue cloud-free mosaick median 2019 s1	https://registry.opendata.aws/sentinel-2/	10	30
lcv_b02_sentinel.s2l2a_iqr_30m_s0..0cm_2019.s1_v0.1.tif	Sentinel 2 Band 2 – Blue cloud-free mosaick IQR 2019 s1	https://registry.opendata.aws/sentinel-2/	10	30
lcv_b02_sentinel.s2l2a_d_30m_s0..0cm_2019.s2_v0.1.tif	Sentinel 2 Band 2 – Blue cloud-free mosaick median 2019 s2	https://registry.opendata.aws/sentinel-2/	10	30

lcv_b02_sentinel.s2l2a_iqr_30m_s0..0cm_2019.s2_v0.1.tif	Sentinel 2 Band 2 – Blue cloud-free mosaick IQR 2019 s2	https://registry.opendata.aws/sentinel-2/	10	30
lcv_b04_sentinel.s2l2a_d_30m_s0..0cm_2019.s1_v0.1.tif	Sentinel 2 Band 4 – Red cloud-free mosaick median 2019 s1	https://registry.opendata.aws/sentinel-2/	10	30
lcv_b04_sentinel.s2l2a_iqr_30m_s0..0cm_2019.s1_v0.1.tif	Sentinel 2 Band 4 – Red cloud-free mosaick IQR 2019 s1	https://registry.opendata.aws/sentinel-2/	10	30
lcv_b04_sentinel.s2l2a_d_30m_s0..0cm_2019.s2_v0.1.tif	Sentinel 2 Band 4 – Red cloud-free mosaick median 2019 s2	https://registry.opendata.aws/sentinel-2/	10	30
lcv_b04_sentinel.s2l2a_iqr_30m_s0..0cm_2019.s2_v0.1.tif	Sentinel 2 Band 4 – Red cloud-free mosaick IQR 2019 s2	https://registry.opendata.aws/sentinel-2/	10	30
lcv_b8a_sentinel.s2l2a_d_30m_s0..0cm_2019.s1_v0.1.tif	Sentinel 2 Band 8A – Narrow NIR cloud-free mosaick median 2019 s1	https://registry.opendata.aws/sentinel-2/	20	30
lcv_b8a_sentinel.s2l2a_iqr_30m_s0..0cm_2019.s1_v0.1.tif	Sentinel 2 Band 8A – Narrow NIR cloud-free mosaick IQR 2019 s1	https://registry.opendata.aws/sentinel-2/	20	30
lcv_b8a_sentinel.s2l2a_d_30m_s0..0cm_2019.s2_v0.1.tif	Sentinel 2 Band 8A – Narrow NIR cloud-free mosaick median 2019 s2	https://registry.opendata.aws/sentinel-2/	20	30
lcv_b8a_sentinel.s2l2a_iqr_30m_s0..0cm_2019.s2_v0.1.tif	Sentinel 2 Band 8A – Narrow NIR cloud-free mosaick IQR 2019 s2	https://registry.opendata.aws/sentinel-2/	20	30
lcv_b09_sentinel.s2l2a_d_30m_s0..0cm_2019.s1_v0.1.tif	Sentinel 2 Band 9 – Water vapour cloud-free mosaick median 2019 s1	https://registry.opendata.aws/sentinel-2/	60	30
lcv_b09_sentinel.s2l2a_iqr_30m_s0..0cm_2019.s1_v0.1.tif	Sentinel 2 Band 9 – Water vapour cloud-free mosaick IQR 2019 s1	https://registry.opendata.aws/sentinel-2/	60	30
lcv_b09_sentinel.s2l2a_d_30m_s0..0cm_2019.s2_v0.1.tif	Sentinel 2 Band 9 – Water vapour cloud-free mosaick median 2019 s2	https://registry.opendata.aws/sentinel-2/	60	30
lcv_b09_sentinel.s2l2a_iqr_30m_s0..0cm_2019.s2_v0.1.tif	Sentinel 2 Band 9 – Water vapour cloud-free mosaick IQR 2019 s2	https://registry.opendata.aws/sentinel-2/	60	30
lcv_b11_sentinel.s2l2a_d_30m_s0..0cm_2019.s1_v0.1.tif	Sentinel 2 Band 11 – SWIR cloud-free mosaick median 2019 s1	https://registry.opendata.aws/sentinel-2/	20	30
lcv_b11_sentinel.s2l2a_iqr_30m_s0..0cm_2019.s1_v0.1.tif	Sentinel 2 Band 11 – SWIR cloud-free mosaick IQR 2019 s1	https://registry.opendata.aws/sentinel-2/	20	30
lcv_b11_sentinel.s2l2a_d_30m_s0..0cm_2019.s2_v0.1.tif	Sentinel 2 Band 11 – SWIR cloud-free mosaick median 2019 s2	https://registry.opendata.aws/sentinel-2/	20	30
lcv_b11_sentinel.s2l2a_iqr_30m_s0..0cm_2019.s2_v0.1.tif	Sentinel 2 Band 11 – SWIR cloud-free mosaick IQR 2019 s2	https://registry.opendata.aws/sentinel-2/	20	30
lcv_b12_sentinel.s2l2a_d_30m_s0..0cm_2019.s1_v0.1.tif	Sentinel 2 Band 12 – SWIR cloud-free mosaick median 2019 s1	https://registry.opendata.aws/sentinel-2/	20	30
lcv_b12_sentinel.s2l2a_iqr_30m_s0..0cm_2019.s1_v0.1.tif	Sentinel 2 Band 12 – SWIR cloud-free mosaick IQR 2019 s1	https://registry.opendata.aws/sentinel-2/	20	30
lcv_b12_sentinel.s2l2a_d_30m_s0..0cm_2019.s2_v0.1.tif	Sentinel 2 Band 12 – SWIR cloud-free mosaick median 2019 s2	https://registry.opendata.aws/sentinel-2/	20	30
lcv_b12_sentinel.s2l2a_iqr_30m_s0..0cm_2019.s2_v0.1.tif	Sentinel 2 Band 12 – SWIR cloud-free mosaick IQR 2019 s2	https://registry.opendata.aws/sentinel-2/	20	30
dtm_*_aw3d30.nasadem_m_30m_s0..0cm_2018_v0.1.tif	High-resolution geomorphometry layers - slope, eastness, northness,	https://www.eorc.jaxa.jp/ALOS/en/aw3d30/index.htm	30	30

	openess			
dtm_*_aw3d30.nasadem.100m_m_30m_s0..0cm_2018_v0.1.tif	Geomorphometry layers - slope, eastness, northness, openess, twi, catchemnt area, downscaled from 100 m	https://www.eorc.jaxa.jp/ALOS/en/aw3d30/index.htm	100	30
lcv_water.occurance_jrc.surfacewater_p_30m_b0..200cm_1984..2018_v1.1.tif	Surface water occurrence probability. Based on the Pekel et al. (2016).	https://global-surface-water.appspot.com/	30	30
lcv_landsat.red_wri.forestwatch_m_30m_s0..0cm_*_v1.0.tif	Global Forest Change 2000–2018 v1.6 red band cloud free	http://earthenginepartners.appspot.com/science-2013-global-forest	30	30
lcv_landsat.nir_wri.forestwatch_m_30m_s0..0cm_*_v1.0.tif	Global Forest Change 2000–2018 v1.6 NIR band cloud free	http://earthenginepartners.appspot.com/science-2013-global-forest	30	30
lcv_landsat.swir1_wri.forestwatch_m_30m_s0..0cm_*_v1.0.tif	Global Forest Change 2000–2018 v1.6 SWIR1 band cloud free	http://earthenginepartners.appspot.com/science-2013-global-forest	30	30
lcv_landsat.swir2_wri.forestwatch_m_30m_s0..0cm_*_v1.0.tif	Global Forest Change 2000–2018 v1.6 SWIR2 band cloud free	http://earthenginepartners.appspot.com/science-2013-global-forest	30	30
veg_f02dar.hh_alos.palsar_m_30m_s0..0cm_2007_v1.0.tif	ALOS PALSAR HH band from 2007	https://www.eorc.jaxa.jp/ALOS/en/palsar_fnf/fnf_index.htm	20	30
veg_f02dar.hv_alos.palsar_m_30m_s0..0cm_2007_v1.0.tif	ALOS PALSAR HV band from 2007	https://www.eorc.jaxa.jp/ALOS/en/palsar_fnf/fnf_index.htm	20	30
veg_f02dar.hh_alos.palsar_m_30m_s0..0cm_2017_v1.0.tif	ALOS PALSAR HH band from 2017	https://www.eorc.jaxa.jp/ALOS/en/palsar_fnf/fnf_index.htm	20	30
veg_f02dar.hv_alos.palsar_m_30m_s0..0cm_2017_v1.0.tif	ALOS PALSAR HV band from 2017	https://www.eorc.jaxa.jp/ALOS/en/palsar_fnf/fnf_index.htm	20	30
lcv_land.cover_esa.cci.l4_m_30m_s0..0cm_2016_v1.0.tif	ESA CCI Land Cover (LC) map at 20m over Africa based on 1 year of Sentinel-2A observations	http://2016africalandcover20m.esrin.esa.int/	20	30
lcv_land.cover_esa.glc100m.*_p_30m_s0..0cm_2016_v1.0.tif	Global 100m Land Cover maps for 2015	https://land.copernicus.eu/global/content/release-global-100m-land-cover-maps-2015	100	30

Supplement 3: Summary statistics per soil variable with variable importance (Random Forest) and summary model accuracies. Derived from mlr package using the https://mlr.mlr-org.com/reference/makeStackedLearner.html function.

```
Results of ensemble model fitting 'ranger', 'xgboost', 'glmnet', 'deepnet':

Variable: clay_tot_psa
R-square: 0.746
Fitted values sd: 16.5
RMSE: 9.63

Random forest model:
Call:
stats::lm(formula = f, data = d)

Residuals:
    Min      1Q  Median      3Q     Max
-75.803  -4.512  -0.178   3.748  82.146

Coefficients:
               Estimate Std. Error t value Pr(>|t|)
(Intercept)    4.494652   8.914671   0.504  0.61413
regr.ranger    1.076957   0.003611 298.210  < 2e-16 ***
regr.xgboost  -0.012617   0.004678  -2.697  0.00699 **
regr.cubist    0.030730   0.003930   7.820 5.32e-15 ***
regr.nnet     -0.238376   0.365390  -0.652  0.51415
regr.cvglmnet -0.044547   0.004379 -10.174  < 2e-16 ***
---
Signif. codes:  0 ‘***’ 0.001 ‘**’ 0.01 ‘*’ 0.05 ‘.’ 0.1 ‘ ’ 1

Residual standard error: 9.629 on 122269 degrees of freedom
Multiple R-squared:  0.7458,    Adjusted R-squared:  0.7458
F-statistic: 7.175e+04 on 5 and 122269 DF,  p-value: < 2.2e-16


Variable importance:
                                                              variable importance
336                                                          hzn_depth  4016546.0
332           lcv_b12_sentinel.s2l2a_d_30m_s0..0cm_2018..2019.s22_v0.1.tif  1686145.3
2          clm_precipitation_sm2rain.apr_m_1km_s0..0cm_2007..2018_v0.2.tif  1345905.1
166              clm_bioclim.var_chelsa.5_m_1km_s0..0cm_1979..2013_v1.0.tif  1190567.9
89               lcv_surf.refl.b05_mod09a1.pc1_m_500m_s0..0cm_2001_v1.0.tif   669722.1
227              dtm_rough.magnitude_merit.dem_m_250m_s0..0cm_2018_v1.0.tif   669571.9
165              clm_bioclim.var_chelsa.4_m_1km_s0..0cm_1979..2013_v1.0.tif   560918.0
37                clm_lst_mod11a2.dec.day_m_1km_s0..0cm_2000..2017_v1.0.tif   512652.5
208                          dtm_vbf_merit.dem_m_1km_s0..0cm_2017_v1.0.tif   501181.3
325     dtm_vertical.depth_aw3d30.nasadem.100m_m_30m_s0..0cm_2017_v0.1.tif   468159.7
276 clm_cloud.fraction_earthenv.modis.mar_p_1km_s0..0cm_2000..2015_v1.0.tif   426687.5
274 clm_cloud.fraction_earthenv.modis.jul_p_1km_s0..0cm_2000..2015_v1.0.tif   426586.6
221                   dtm_elevation_merit.dem_m_250m_s0..0cm_2017_v1.0.tif   418043.6
```

```
18         clm_precipitation_sm2rain.may_m_1km_s0..0cm_2007..2018_v0.2.tif   405753.7
271 clm_cloud.fraction_earthenv.modis.dec_p_1km_s0..0cm_2000..2015_v1.0.tif   393397.9
1       clm_precipitation_sm2rain.annual_m_1km_s0..0cm_2007..2018_v0.2.tif   392724.8
72             clm_lst_mod11a2.oct.night_m_1km_s0..0cm_2000..2017_v1.0.tif   383326.7
156            clm_bioclim.var_chelsa.10_m_1km_s0..0cm_1979..2013_v1.0.tif   372853.1
185   clm_direct.irradiation_solar.atlas.kwhm2.10_m_1km_s0..0cm_2016_v1.tif   369821.6
290               dtm_elevation_aw3d30.nasadem_m_30m_s0..0cm_2017_v0.1.tif   364986.1
275 clm_cloud.fraction_earthenv.modis.jun_p_1km_s0..0cm_2000..2015_v1.0.tif   363420.9
298           lcv_b09_sentinel.s2l2a_d_30m_s0..0cm_2018..2019.s22_v0.1.tif   336227.7
42              clm_lst_mod11a2.feb.day_sd_1km_s0..0cm_2000..2017_v1.0.tif   308694.5
19     clm_precipitation_sm2rain.may_sd.10_10km_s0..0cm_2007..2018_v1.0.tif   302853.6
9      clm_precipitation_sm2rain.feb_sd.10_10km_s0..0cm_2007..2018_v1.0.tif   294082.8
76             clm_lst_mod11a2.sep.night_m_1km_s0..0cm_2000..2017_v1.0.tif   293483.8
13     clm_precipitation_sm2rain.jul_sd.10_10km_s0..0cm_2007..2018_v1.0.tif   291769.8
24         clm_precipitation_sm2rain.sep_m_1km_s0..0cm_2007..2018_v0.2.tif   285421.6
278 clm_cloud.fraction_earthenv.modis.nov_p_1km_s0..0cm_2000..2015_v1.0.tif   283346.6
59          clm_lst_mod11a2.mar.daynight_m_1km_s0..0cm_2000..2017_v1.0.tif   282282.1
287          lcv_landsat.swir1_wri.forestwatch_m_30m_s0..0cm_2015_v1.0.tif   280958.3
229                  dtm_roughness_merit.dem_m_250m_s0..0cm_2018_v1.0.tif   280836.8
58              clm_lst_mod11a2.mar.day_sd_1km_s0..0cm_2000..2017_v1.0.tif   278276.8
308                 veg_f02dar.hv_alos.palsar_m_30m_s0..0cm_2007_v1.0.tif   268674.5
279 clm_cloud.fraction_earthenv.modis.oct_p_1km_s0..0cm_2000..2015_v1.0.tif   266859.4
130           veg_fapar_proba.v.jul_u.975_250m_s0..0cm_2014..2019_v1.0.tif   263512.5
267        clm_wind.speed_terraclimate.sep_m_5km_s0..0cm_1998..2018_v1.tif   261704.9
54              clm_lst_mod11a2.jun.day_sd_1km_s0..0cm_2000..2017_v1.0.tif   254202.7
157            clm_bioclim.var_chelsa.11_m_1km_s0..0cm_1979..2013_v1.0.tif   243823.4
269 clm_cloud.fraction_earthenv.modis.apr_p_1km_s0..0cm_2000..2015_v1.0.tif   242089.7
84              lcv_surf.refl.b02_mod09a1.pc2_m_500m_s0..0cm_2001_v1.0.tif   241183.5
43          clm_lst_mod11a2.feb.daynight_m_1km_s0..0cm_2000..2017_v1.0.tif   240861.2
333                 veg_f02dar.hv_alos.palsar_m_30m_s0..0cm_2017_v1.0.tif   240647.4
29               clm_lst_mod11a2.apr.day_m_1km_s0..0cm_2000..2017_v1.0.tif   239800.7
70              clm_lst_mod11a2.oct.day_sd_1km_s0..0cm_2000..2017_v1.0.tif   238443.9
34              clm_lst_mod11a2.aug.day_sd_1km_s0..0cm_2000..2017_v1.0.tif   236980.9
62              clm_lst_mod11a2.may.day_sd_1km_s0..0cm_2000..2017_v1.0.tif   234664.6
193                        dtm_dvm2_merit.dem_m_2km_s0..0cm_2017_v1.0.tif   234453.0
326         lcv_b11_sentinel.s2l2a_iqr_30m_s0..0cm_2018..2019.s22_v0.1.tif   234276.0
161            clm_bioclim.var_chelsa.16_m_1km_s0..0cm_1979..2013_v1.0.tif   234120.0

Results of ensemble model fitting 'ranger', 'xgboost', 'glmnet', 'deepnet':

Variable: db_od
R-square: 0.819
Fitted values sd: 0.269
RMSE: 0.126

Random forest model:
```

```
Call:
stats::lm(formula = f, data = d)

Residuals:
     Min       1Q   Median       3Q      Max 
-1.06778 -0.06450  0.00215  0.06585  0.90016 

Coefficients:
              Estimate Std. Error t value Pr(>|t|)    
(Intercept)   -0.05538    0.04860  -1.140  0.25451    
regr.ranger    0.86305    0.01577  54.733  < 2e-16 ***
regr.xgboost   0.15383    0.01651   9.315  < 2e-16 ***
regr.cubist    0.02039    0.01113   1.832  0.06695 .  
regr.nnet      0.03465    0.03710   0.934  0.35036    
regr.cvglmnet -0.03021    0.01032  -2.927  0.00343 ** 
---
Signif. codes:  0 '***' 0.001 '**' 0.01 '*' 0.05 '.' 0.1 ' ' 1

Residual standard error: 0.1263 on 13565 degrees of freedom
Multiple R-squared:  0.8194,    Adjusted R-squared:  0.8193 
F-statistic: 1.231e+04 on 5 and 13565 DF,  p-value: < 2.2e-16


Variable importance:
                                                                 variable importance
221                      dtm_elevation_merit.dem_m_250m_s0..0cm_2017_v1.0.tif 173.055853
311             lcv_landsat.swir1_wri.forestwatch_m_30m_s0..0cm_2018_v1.0.tif  88.203943
209                             dtm_vbf_merit.dem_m_2km_s0..0cm_2017_v1.0.tif  62.529811
301              lcv_b11_sentinel.s2l2a_d_30m_s0..0cm_2018..2019.s22_v0.1.tif  50.220640
336                                                                 hzn_depth  49.436356
290                    dtm_elevation_aw3d30.nasadem_m_30m_s0..0cm_2017_v0.1.tif  43.942565
227                  dtm_rough.magnitude_merit.dem_m_250m_s0..0cm_2018_v1.0.tif  38.466574
286             lcv_landsat.swir1_wri.forestwatch_m_30m_s0..0cm_2000_v1.0.tif  30.560528
73                  clm_lst_mod11a2.sep.day_m_1km_s0..0cm_2000..2017_v1.0.tif  28.298537
1          clm_precipitation_sm2rain.annual_m_1km_s0..0cm_2007..2018_v0.2.tif  23.243982
208                             dtm_vbf_merit.dem_m_1km_s0..0cm_2017_v1.0.tif  14.701368
158               clm_bioclim.var_chelsa.12_m_1km_s0..0cm_1979..2013_v1.0.tif  13.918615
3        clm_precipitation_sm2rain.apr_sd.10_10km_s0..0cm_2007..2018_v1.0.tif  13.554058
101                 lcv_surf.refl.b07_mod09a1.pc1_m_500m_s0..0cm_2001_v1.0.tif  11.872374
63              clm_lst_mod11a2.may.daynight_m_1km_s0..0cm_2000..2017_v1.0.tif  10.289871
269    clm_cloud.fraction_earthenv.modis.apr_p_1km_s0..0cm_2000..2015_v1.0.tif  10.064493
268 clm_cloud.fraction_earthenv.modis.annual_m_1km_s0..0cm_2000..2015_v1.0.tif   9.789589
272    clm_cloud.fraction_earthenv.modis.feb_p_1km_s0..0cm_2000..2015_v1.0.tif   9.683845
168                clm_bioclim.var_chelsa.7_m_1km_s0..0cm_1979..2013_v1.0.tif   8.983756
273    clm_cloud.fraction_earthenv.modis.jan_p_1km_s0..0cm_2000..2015_v1.0.tif   8.061213
167                clm_bioclim.var_chelsa.6_m_1km_s0..0cm_1979..2013_v1.0.tif   7.667438
```

```
14            clm_precipitation_sm2rain.jun_m_1km_s0..0cm_2007..2018_v0.2.tif   7.381931
89                 lcv_surf.refl.b05_mod09a1.pc1_m_500m_s0..0cm_2001_v1.0.tif   7.167184
28             clm_lst_mod11a2.annual.night_m_1km_s0..0cm_2000..2017_v1.0.tif   6.899234
69                  clm_lst_mod11a2.oct.day_m_1km_s0..0cm_2000..2017_v1.0.tif   6.897999
278   clm_cloud.fraction_earthenv.modis.nov_p_1km_s0..0cm_2000..2015_v1.0.tif   6.877684
17       clm_precipitation_sm2rain.mar_sd.10_10km_s0..0cm_2007..2018_v1.0.tif   6.829219
234                             dtm_vbf_merit.dem_m_250m_s0..0cm_2017_v1.0.tif   6.753470
161               clm_bioclim.var_chelsa.16_m_1km_s0..0cm_1979..2013_v1.0.tif   6.717086
36                clm_lst_mod11a2.aug.night_m_1km_s0..0cm_2000..2017_v1.0.tif   6.150320
24            clm_precipitation_sm2rain.sep_m_1km_s0..0cm_2007..2018_v0.2.tif   6.029270
280   clm_cloud.fraction_earthenv.modis.sep_p_1km_s0..0cm_2000..2015_v1.0.tif   5.882436
27              clm_lst_mod11a2.annual.day_sd_1km_s0..0cm_2000..2017_v1.0.tif   5.759884
30                 clm_lst_mod11a2.apr.day_sd_1km_s0..0cm_2000..2017_v1.0.tif   5.529642
60                clm_lst_mod11a2.mar.night_m_1km_s0..0cm_2000..2017_v1.0.tif   5.518799
277   clm_cloud.fraction_earthenv.modis.may_p_1km_s0..0cm_2000..2015_v1.0.tif   5.290183
57                  clm_lst_mod11a2.mar.day_m_1km_s0..0cm_2000..2017_v1.0.tif   5.257706
175                                                          af_lithology_X9   5.240793
192                             dtm_dvm2_merit.dem_m_1km_s0..0cm_2017_v1.0.tif   5.157378
55             clm_lst_mod11a2.jun.daynight_m_1km_s0..0cm_2000..2017_v1.0.tif   4.874207
83                 lcv_surf.refl.b02_mod09a1.pc1_m_500m_s0..0cm_2001_v1.0.tif   4.848989
65                  clm_lst_mod11a2.nov.day_m_1km_s0..0cm_2000..2017_v1.0.tif   4.823740
4             clm_precipitation_sm2rain.aug_m_1km_s0..0cm_2007..2018_v0.2.tif   4.754339
38                 clm_lst_mod11a2.dec.day_sd_1km_s0..0cm_2000..2017_v1.0.tif   4.649781
328                lcv_b04_sentinel.s2l2a_d_30m_s0..0cm_2018..2019.s12_v0.1.tif   4.546480
91                 lcv_surf.refl.b05_mod09a1.pc3_m_500m_s0..0cm_2001_v1.0.tif   4.486198
16            clm_precipitation_sm2rain.mar_m_1km_s0..0cm_2007..2018_v0.2.tif   4.430792
275   clm_cloud.fraction_earthenv.modis.jun_p_1km_s0..0cm_2000..2015_v1.0.tif   4.305822
184   clm_diffuse.irradiation_solar.atlas.kwhm2.100_m_1km_s0..0cm_2016_v1.tif   4.303449
47             clm_lst_mod11a2.jan.daynight_m_1km_s0..0cm_2000..2017_v1.0.tif   4.220544

Results of ensemble model fitting 'ranger', 'xgboost', 'glmnet', 'deepnet':

Variable: dbr
R-square: 0.429
Fitted values sd: 37.5
RMSE: 43.3

Random forest model:
Call:
stats::lm(formula = f, data = d)

Residuals:
     Min       1Q   Median       3Q      Max
-249.840   -0.724    2.667   12.198  242.184

Coefficients:
```

```
               Estimate Std. Error t value Pr(>|t|)
(Intercept)   650.69680  638.91097   1.018   0.3085
regr.ranger     0.85465    0.01640  52.128  < 2e-16 ***
regr.xgboost    0.13640    0.01874   7.280 3.43e-13 ***
regr.cubist     0.11993    0.01211   9.903  < 2e-16 ***
regr.nnet      -2.89697    2.78062  -1.042   0.2975
regr.cvglmnet  -0.04644    0.01536  -3.024   0.0025 **
---
Signif. codes:  0 '***' 0.001 '**' 0.01 '*' 0.05 '.' 0.1 ' ' 1

Residual standard error: 43.26 on 28054 degrees of freedom
Multiple R-squared:  0.429, Adjusted R-squared:  0.4289
F-statistic:  4216 on 5 and 28054 DF,  p-value: < 2.2e-16


Variable importance:
                                                                  variable importance
22         clm_precipitation_sm2rain.oct_m_1km_s0..0cm_2007..2018_v0.2.tif  1625434.4
158            clm_bioclim.var_chelsa.12_m_1km_s0..0cm_1979..2013_v1.0.tif  1595999.6
168             clm_bioclim.var_chelsa.7_m_1km_s0..0cm_1979..2013_v1.0.tif  1452172.3
108          veg_fapar_proba.v.annnual_d_250m_s0..0cm_2014..2019_v1.0.tif  1270840.8
159            clm_bioclim.var_chelsa.13_m_1km_s0..0cm_1979..2013_v1.0.tif  1098804.1
2          clm_precipitation_sm2rain.apr_m_1km_s0..0cm_2007..2018_v0.2.tif  1095576.0
208                          dtm_vbf_merit.dem_m_1km_s0..0cm_2017_v1.0.tif  1082679.9
272 clm_cloud.fraction_earthenv.modis.feb_p_1km_s0..0cm_2000..2015_v1.0.tif  1034668.1
20         clm_precipitation_sm2rain.nov_m_1km_s0..0cm_2007..2018_v0.2.tif  1027417.3
84              lcv_surf.refl.b02_mod09a1.pc2_m_500m_s0..0cm_2001_v1.0.tif  1021259.8
266        clm_wind.speed_terraclimate.oct_m_5km_s0..0cm_1998..2018_v1.tif  1018394.5
262        clm_wind.speed_terraclimate.jun_m_5km_s0..0cm_1998..2018_v1.tif  1013247.0
165             clm_bioclim.var_chelsa.4_m_1km_s0..0cm_1979..2013_v1.0.tif   976202.4
36             clm_lst_mod11a2.aug.night_m_1km_s0..0cm_2000..2017_v1.0.tif   975607.1
271 clm_cloud.fraction_earthenv.modis.dec_p_1km_s0..0cm_2000..2015_v1.0.tif   963765.6
290               dtm_elevation_aw3d30.nasadem_m_30m_s0..0cm_2017_v0.1.tif   945470.1
147               veg_fapar_proba.v.oct_d_250m_s0..0cm_2014..2019_v1.0.tif   918678.7
234                         dtm_vbf_merit.dem_m_250m_s0..0cm_2017_v1.0.tif   888139.6
151               veg_fapar_proba.v.sep_d_250m_s0..0cm_2014..2019_v1.0.tif   886679.3
273 clm_cloud.fraction_earthenv.modis.jan_p_1km_s0..0cm_2000..2015_v1.0.tif   856595.1
6          clm_precipitation_sm2rain.dec_m_1km_s0..0cm_2007..2018_v0.2.tif   835007.3
275 clm_cloud.fraction_earthenv.modis.jun_p_1km_s0..0cm_2000..2015_v1.0.tif   834660.3
164             clm_bioclim.var_chelsa.3_m_1km_s0..0cm_1979..2013_v1.0.tif   818492.8
184 clm_diffuse.irradiation_solar.atlas.kwhm2.100_m_1km_s0..0cm_2016_v1.tif   794544.7
124               veg_fapar_proba.v.jan_d_250m_s0..0cm_2014..2019_v1.0.tif   792837.5
39          clm_lst_mod11a2.dec.daynight_m_1km_s0..0cm_2000..2017_v1.0.tif   747307.1
131               veg_fapar_proba.v.jun_d_250m_s0..0cm_2014..2019_v1.0.tif   697004.2
192                         dtm_dvm2_merit.dem_m_1km_s0..0cm_2017_v1.0.tif   675968.6
260        clm_wind.speed_terraclimate.jan_m_5km_s0..0cm_1998..2018_v1.tif   669228.5
```

```
279 clm_cloud.fraction_earthenv.modis.oct_p_1km_s0..0cm_2000..2015_v1.0.tif   668248.3
269 clm_cloud.fraction_earthenv.modis.apr_p_1km_s0..0cm_2000..2015_v1.0.tif   662771.0
14          clm_precipitation_sm2rain.jun_m_1km_s0..0cm_2007..2018_v0.2.tif   631001.7
89               lcv_surf.refl.b05_mod09a1.pc1_m_500m_s0..0cm_2001_v1.0.tif   626569.5
270 clm_cloud.fraction_earthenv.modis.aug_p_1km_s0..0cm_2000..2015_v1.0.tif   623979.1
61                clm_lst_mod11a2.may.day_m_1km_s0..0cm_2000..2017_v1.0.tif   623073.5
256         clm_wind.speed_terraclimate.apr_m_5km_s0..0cm_1998..2018_v1.tif   613790.4
263         clm_wind.speed_terraclimate.mar_m_5km_s0..0cm_1998..2018_v1.tif   609655.3
120               veg_fapar_proba.v.feb_d_250m_s0..0cm_2014..2019_v1.0.tif   609362.3
325      dtm_vertical.depth_aw3d30.nasadem.100m_m_30m_s0..0cm_2017_v0.1.tif   609128.9
75           clm_lst_mod11a2.sep.daynight_m_1km_s0..0cm_2000..2017_v1.0.tif   607180.4
185   clm_direct.irradiation_solar.atlas.kwhm2.10_m_1km_s0..0cm_2016_v1.tif   606570.0
42               clm_lst_mod11a2.feb.day_sd_1km_s0..0cm_2000..2017_v1.0.tif   601597.1
148            veg_fapar_proba.v.oct_l.025_250m_s0..0cm_2014..2019_v1.0.tif   599634.2
157             clm_bioclim.var_chelsa.11_m_1km_s0..0cm_1979..2013_v1.0.tif   592305.8
109               veg_fapar_proba.v.apr_d_250m_s0..0cm_2014..2019_v1.0.tif   585096.2
23     clm_precipitation_sm2rain.oct_sd.10_10km_s0..0cm_2007..2018_v1.0.tif   580821.3
4           clm_precipitation_sm2rain.aug_m_1km_s0..0cm_2007..2018_v0.2.tif   560969.5
265         clm_wind.speed_terraclimate.nov_m_5km_s0..0cm_1998..2018_v1.tif   560530.1
95               lcv_surf.refl.b06_mod09a1.pc1_m_500m_s0..0cm_2001_v1.0.tif   558936.2
224                     dtm_northness_merit.dem_m_250m_s0..0cm_2018_v1.0.tif   555155.0

Results of ensemble model fitting 'ranger', 'xgboost', 'glmnet', 'deepnet':

Variable: log.al_mehlich3
R-square: 0.881
Fitted values sd: 0.872
RMSE: 0.321

Random forest model:
Call:
stats::lm(formula = f, data = d)

Residuals:
    Min      1Q  Median      3Q     Max 
-5.7042 -0.1036  0.0059  0.1189  3.3777 

Coefficients:
               Estimate Std. Error t value Pr(>|t|)    
(Intercept)   -0.675492   2.771906  -0.244    0.807    
regr.ranger    0.879567   0.005464 160.969   <2e-16 ***
regr.xgboost   0.071537   0.005813  12.306   <2e-16 ***
regr.cubist    0.150157   0.004553  32.979   <2e-16 ***
regr.nnet      0.087603   0.431261   0.203    0.839    
regr.cvglmnet -0.084440   0.003182 -26.534   <2e-16 ***
---
```

```
Signif. codes:  0 ‘***’ 0.001 ‘**’ 0.01 ‘*’ 0.05 ‘.’ 0.1 ‘ ’ 1

Residual standard error: 0.3208 on 63551 degrees of freedom
Multiple R-squared:  0.8808,    Adjusted R-squared:  0.8808
F-statistic: 9.391e+04 on 5 and 63551 DF,  p-value: < 2.2e-16


Variable importance:
                                                                     variable importance
336                                                                 hzn_depth 13418.5825
165                  clm_bioclim.var_chelsa.4_m_1km_s0..0cm_1979..2013_v1.0.tif  1571.9326
256             clm_wind.speed_terraclimate.apr_m_5km_s0..0cm_1998..2018_v1.tif  1230.9573
18              clm_precipitation_sm2rain.may_m_1km_s0..0cm_2007..2018_v0.2.tif  1000.2514
39               clm_lst_mod11a2.dec.daynight_m_1km_s0..0cm_2000..2017_v1.0.tif   937.1489
264             clm_wind.speed_terraclimate.may_m_5km_s0..0cm_1998..2018_v1.tif   881.4959
184    clm_diffuse.irradiation_solar.atlas.kwhm2.100_m_1km_s0..0cm_2016_v1.tif   867.0038
257             clm_wind.speed_terraclimate.aug_m_5km_s0..0cm_1998..2018_v1.tif   846.6338
158                 clm_bioclim.var_chelsa.12_m_1km_s0..0cm_1979..2013_v1.0.tif   683.9056
56                  clm_lst_mod11a2.jun.night_m_1km_s0..0cm_2000..2017_v1.0.tif   649.7619
16              clm_precipitation_sm2rain.mar_m_1km_s0..0cm_2007..2018_v0.2.tif   626.1463
167                  clm_bioclim.var_chelsa.6_m_1km_s0..0cm_1979..2013_v1.0.tif   623.9146
290                   dtm_elevation_aw3d30.nasadem_m_30m_s0..0cm_2017_v0.1.tif   619.9115
6               clm_precipitation_sm2rain.dec_m_1km_s0..0cm_2007..2018_v0.2.tif   570.6529
266             clm_wind.speed_terraclimate.oct_m_5km_s0..0cm_1998..2018_v1.tif   536.3592
156                 clm_bioclim.var_chelsa.10_m_1km_s0..0cm_1979..2013_v1.0.tif   509.8736
20              clm_precipitation_sm2rain.nov_m_1km_s0..0cm_2007..2018_v0.2.tif   498.8553
157                 clm_bioclim.var_chelsa.11_m_1km_s0..0cm_1979..2013_v1.0.tif   495.6036
260             clm_wind.speed_terraclimate.jan_m_5km_s0..0cm_1998..2018_v1.tif   492.4013
49                    clm_lst_mod11a2.jul.day_m_1km_s0..0cm_2000..2017_v1.0.tif   463.4539
30                   clm_lst_mod11a2.apr.day_sd_1km_s0..0cm_2000..2017_v1.0.tif   462.5531
166                  clm_bioclim.var_chelsa.5_m_1km_s0..0cm_1979..2013_v1.0.tif   441.9174
72                  clm_lst_mod11a2.oct.night_m_1km_s0..0cm_2000..2017_v1.0.tif   434.6131
4               clm_precipitation_sm2rain.aug_m_1km_s0..0cm_2007..2018_v0.2.tif   418.7509
63               clm_lst_mod11a2.may.daynight_m_1km_s0..0cm_2000..2017_v1.0.tif   415.5855
159                 clm_bioclim.var_chelsa.13_m_1km_s0..0cm_1979..2013_v1.0.tif   409.0120
277    clm_cloud.fraction_earthenv.modis.may_p_1km_s0..0cm_2000..2015_v1.0.tif   399.9598
95                   lcv_surf.refl.b06_mod09a1.pc1_m_500m_s0..0cm_2001_v1.0.tif   385.7886
10              clm_precipitation_sm2rain.jan_m_1km_s0..0cm_2007..2018_v0.2.tif   384.5702
268 clm_cloud.fraction_earthenv.modis.annual_m_1km_s0..0cm_2000..2015_v1.0.tif   381.4038
161                 clm_bioclim.var_chelsa.16_m_1km_s0..0cm_1979..2013_v1.0.tif   375.0136
269    clm_cloud.fraction_earthenv.modis.apr_p_1km_s0..0cm_2000..2015_v1.0.tif   368.3472
209                              dtm_vbf_merit.dem_m_2km_s0..0cm_2017_v1.0.tif   367.8038
273    clm_cloud.fraction_earthenv.modis.jan_p_1km_s0..0cm_2000..2015_v1.0.tif   362.4353
52                  clm_lst_mod11a2.jul.night_m_1km_s0..0cm_2000..2017_v1.0.tif   358.6965
61                    clm_lst_mod11a2.may.day_m_1km_s0..0cm_2000..2017_v1.0.tif   343.3633
41                    clm_lst_mod11a2.feb.day_m_1km_s0..0cm_2000..2017_v1.0.tif   334.2879
```

```
276    clm_cloud.fraction_earthenv.modis.mar_p_1km_s0..0cm_2000..2015_v1.0.tif   332.7767
259            clm_wind.speed_terraclimate.feb_m_5km_s0..0cm_1998..2018_v1.tif   330.3643
65                   clm_lst_mod11a2.nov.day_m_1km_s0..0cm_2000..2017_v1.0.tif   323.2967
33                   clm_lst_mod11a2.aug.day_m_1km_s0..0cm_2000..2017_v1.0.tif   319.7172
155                clm_bioclim.var_chelsa.1_m_1km_s0..0cm_1979..2013_v1.0.tif   306.0843
271    clm_cloud.fraction_earthenv.modis.dec_p_1km_s0..0cm_2000..2015_v1.0.tif   300.7234
265            clm_wind.speed_terraclimate.nov_m_5km_s0..0cm_1998..2018_v1.tif   292.0814
84                 lcv_surf.refl.b02_mod09a1.pc2_m_500m_s0..0cm_2001_v1.0.tif   290.4675
85                 lcv_surf.refl.b02_mod09a1.pc3_m_500m_s0..0cm_2001_v1.0.tif   287.7645
270    clm_cloud.fraction_earthenv.modis.aug_p_1km_s0..0cm_2000..2015_v1.0.tif   285.8978
7         clm_precipitation_sm2rain.dec_sd.10_10km_s0..0cm_2007..2018_v1.0.tif   282.9229
101                lcv_surf.refl.b07_mod09a1.pc1_m_500m_s0..0cm_2001_v1.0.tif   272.3740
325        dtm_vertical.depth_aw3d30.nasadem.100m_m_30m_s0..0cm_2017_v0.1.tif   271.9390

Results of ensemble model fitting 'ranger', 'xgboost', 'glmnet', 'deepnet':

Variable: log.ca_mehlich3
R-square: 0.84
Fitted values sd: 1.24
RMSE: 0.543

Random forest model:
Call:
stats::lm(formula = f, data = d)

Residuals:
    Min      1Q  Median      3Q     Max 
-6.0376 -0.2577  0.0076  0.2756  5.3825 

Coefficients:
               Estimate Std. Error t value Pr(>|t|)    
(Intercept)    5.737959   3.850998   1.490    0.136    
regr.ranger    1.054018   0.003175 331.978  < 2e-16 ***
regr.xgboost  -0.030930   0.003939  -7.853  4.1e-15 ***
regr.cubist    0.061829   0.003561  17.364  < 2e-16 ***
regr.nnet     -0.855297   0.561006  -1.525    0.127    
regr.cvglmnet -0.065040   0.003225 -20.166  < 2e-16 ***
---
Signif. codes:  0 ‘***’ 0.001 ‘**’ 0.01 ‘*’ 0.05 ‘.’ 0.1 ‘ ’ 1

Residual standard error: 0.5428 on 144593 degrees of freedom
Multiple R-squared:  0.8403,    Adjusted R-squared:  0.8402 
F-statistic: 1.521e+05 on 5 and 144593 DF,  p-value: < 2.2e-16


Variable importance:
```

	variable	importance
184	clm_diffuse.irradiation_solar.atlas.kwhm2.100_m_1km_s0..0cm_2016_v1.tif	25629.557
336	hzn_depth	15475.129
136	veg_fapar_proba.v.mar_l.025_250m_s0..0cm_2014..2019_v1.0.tif	12867.069
63	clm_lst_mod11a2.may.daynight_m_1km_s0..0cm_2000..2017_v1.0.tif	11012.360
164	clm_bioclim.var_chelsa.3_m_1km_s0..0cm_1979..2013_v1.0.tif	9706.161
62	clm_lst_mod11a2.may.day_sd_1km_s0..0cm_2000..2017_v1.0.tif	9286.497
41	clm_lst_mod11a2.feb.day_m_1km_s0..0cm_2000..2017_v1.0.tif	8707.913
20	clm_precipitation_sm2rain.nov_m_1km_s0..0cm_2007..2018_v0.2.tif	6540.222
165	clm_bioclim.var_chelsa.4_m_1km_s0..0cm_1979..2013_v1.0.tif	4443.386
58	clm_lst_mod11a2.mar.day_sd_1km_s0..0cm_2000..2017_v1.0.tif	3945.553
8	clm_precipitation_sm2rain.feb_m_1km_s0..0cm_2007..2018_v0.2.tif	3827.796
89	lcv_surf.refl.b05_mod09a1.pc1_m_500m_s0..0cm_2001_v1.0.tif	3652.756
121	veg_fapar_proba.v.feb_l.025_250m_s0..0cm_2014..2019_v1.0.tif	3485.488
22	clm_precipitation_sm2rain.oct_m_1km_s0..0cm_2007..2018_v0.2.tif	3031.816
209	dtm_vbf_merit.dem_m_2km_s0..0cm_2017_v1.0.tif	2890.768
55	clm_lst_mod11a2.jun.daynight_m_1km_s0..0cm_2000..2017_v1.0.tif	2800.209
12	clm_precipitation_sm2rain.jul_m_1km_s0..0cm_2007..2018_v0.2.tif	2694.930
33	clm_lst_mod11a2.aug.day_m_1km_s0..0cm_2000..2017_v1.0.tif	2638.236
84	lcv_surf.refl.b02_mod09a1.pc2_m_500m_s0..0cm_2001_v1.0.tif	2467.229
30	clm_lst_mod11a2.apr.day_sd_1km_s0..0cm_2000..2017_v1.0.tif	2393.608
54	clm_lst_mod11a2.jun.day_sd_1km_s0..0cm_2000..2017_v1.0.tif	2346.238
227	dtm_rough.magnitude_merit.dem_m_250m_s0..0cm_2018_v1.0.tif	2279.393
135	veg_fapar_proba.v.mar_d_250m_s0..0cm_2014..2019_v1.0.tif	2138.859
10	clm_precipitation_sm2rain.jan_m_1km_s0..0cm_2007..2018_v0.2.tif	1903.479
163	clm_bioclim.var_chelsa.2_m_1km_s0..0cm_1979..2013_v1.0.tif	1857.197
19	clm_precipitation_sm2rain.may_sd.10_10km_s0..0cm_2007..2018_v1.0.tif	1846.464
159	clm_bioclim.var_chelsa.13_m_1km_s0..0cm_1979..2013_v1.0.tif	1783.455
275	clm_cloud.fraction_earthenv.modis.jun_p_1km_s0..0cm_2000..2015_v1.0.tif	1778.113
278	clm_cloud.fraction_earthenv.modis.nov_p_1km_s0..0cm_2000..2015_v1.0.tif	1753.762
45	clm_lst_mod11a2.jan.day_m_1km_s0..0cm_2000..2017_v1.0.tif	1493.797
161	clm_bioclim.var_chelsa.16_m_1km_s0..0cm_1979..2013_v1.0.tif	1484.762
298	lcv_b09_sentinel.s2l2a_d_30m_s0..0cm_2018..2019.s22_v0.1.tif	1482.038
65	clm_lst_mod11a2.nov.day_m_1km_s0..0cm_2000..2017_v1.0.tif	1445.098
166	clm_bioclim.var_chelsa.5_m_1km_s0..0cm_1979..2013_v1.0.tif	1340.312
320	lcv_b09_sentinel.s2l2a_d_30m_s0..0cm_2018..2019.s12_v0.1.tif	1310.983
269	clm_cloud.fraction_earthenv.modis.apr_p_1km_s0..0cm_2000..2015_v1.0.tif	1306.876
90	lcv_surf.refl.b05_mod09a1.pc2_m_500m_s0..0cm_2001_v1.0.tif	1306.185
279	clm_cloud.fraction_earthenv.modis.oct_p_1km_s0..0cm_2000..2015_v1.0.tif	1292.117
204	dtm_twi_merit.dem_m_2km_s0..0cm_2017_v1.0.tif	1274.419
75	clm_lst_mod11a2.sep.daynight_m_1km_s0..0cm_2000..2017_v1.0.tif	1263.827
229	dtm_roughness_merit.dem_m_250m_s0..0cm_2018_v1.0.tif	1256.875
208	dtm_vbf_merit.dem_m_1km_s0..0cm_2017_v1.0.tif	1248.525
216	dtm_dev.magnitude_merit.dem_m_250m_s0..0cm_2018_v1.0.tif	1245.920
4	clm_precipitation_sm2rain.aug_m_1km_s0..0cm_2007..2018_v0.2.tif	1245.698
268	clm_cloud.fraction_earthenv.modis.annual_m_1km_s0..0cm_2000..2015_v1.0.tif	1244.454

```
203                          dtm_twi_merit.dem_m_1km_s0..0cm_2017_v1.0.tif   1234.857
259          clm_wind.speed_terraclimate.feb_m_5km_s0..0cm_1998..2018_v1.tif   1234.189
51            clm_lst_mod11a2.jul.daynight_m_1km_s0..0cm_2000..2017_v1.0.tif   1223.667
69                 clm_lst_mod11a2.oct.day_m_1km_s0..0cm_2000..2017_v1.0.tif   1206.937
277   clm_cloud.fraction_earthenv.modis.may_p_1km_s0..0cm_2000..2015_v1.0.tif   1201.371

Results of ensemble model fitting 'ranger', 'xgboost', 'glmnet', 'deepnet':

Variable: log.c_tot
R-square: 0.794
Fitted values sd: 0.571
RMSE: 0.291

Random forest model:
Call:
stats::lm(formula = f, data = d)

Residuals:
     Min       1Q   Median       3Q      Max
-2.70312 -0.16714 -0.00549  0.15691  3.01116

Coefficients:
               Estimate Std. Error t value Pr(>|t|)
(Intercept)    0.025841   0.032713   0.790 0.429570
regr.ranger    0.902240   0.008462 106.619  < 2e-16 ***
regr.xgboost   0.066535   0.008145   8.169 3.18e-16 ***
regr.cubist    0.145730   0.006927  21.039  < 2e-16 ***
regr.nnet     -0.048957   0.013466  -3.636 0.000278 ***
regr.cvglmnet -0.075212   0.005556 -13.537  < 2e-16 ***
---
Signif. codes:  0 '***' 0.001 '**' 0.01 '*' 0.05 '.' 0.1 ' ' 1

Residual standard error: 0.291 on 50140 degrees of freedom
Multiple R-squared:  0.7938,    Adjusted R-squared:  0.7938
F-statistic: 3.861e+04 on 5 and 50140 DF,  p-value: < 2.2e-16


Variable importance:
                                                                   variable importance
301          lcv_b11_sentinel.s2l2a_d_30m_s0..0cm_2018..2019.s22_v0.1.tif 1529.37519
1        clm_precipitation_sm2rain.annual_m_1km_s0..0cm_2007..2018_v0.2.tif 1332.97927
336                                                               hzn_depth 1067.10077
286          lcv_landsat.swir1_wri.forestwatch_m_30m_s0..0cm_2000_v1.0.tif  927.23617
227             dtm_rough.magnitude_merit.dem_m_250m_s0..0cm_2018_v1.0.tif  877.53084
168             clm_bioclim.var_chelsa.7_m_1km_s0..0cm_1979..2013_v1.0.tif  563.68050
261        clm_wind.speed_terraclimate.jul_m_5km_s0..0cm_1998..2018_v1.tif  419.49978
```

```
95              lcv_surf.refl.b06_mod09a1.pc1_m_500m_s0..0cm_2001_v1.0.tif  404.49441
274 clm_cloud.fraction_earthenv.modis.jul_p_1km_s0..0cm_2000..2015_v1.0.tif  378.58867
165             clm_bioclim.var_chelsa.4_m_1km_s0..0cm_1979..2013_v1.0.tif  338.85521
18         clm_precipitation_sm2rain.may_m_1km_s0..0cm_2007..2018_v0.2.tif  328.63451
27           clm_lst_mod11a2.annual.day_sd_1km_s0..0cm_2000..2017_v1.0.tif  312.28715
334            lcv_landsat.nir_wri.forestwatch_m_30m_s0..0cm_2015_v1.0.tif  293.18473
83              lcv_surf.refl.b02_mod09a1.pc1_m_500m_s0..0cm_2001_v1.0.tif  283.37199
276 clm_cloud.fraction_earthenv.modis.mar_p_1km_s0..0cm_2000..2015_v1.0.tif  259.93640
101             lcv_surf.refl.b07_mod09a1.pc1_m_500m_s0..0cm_2001_v1.0.tif  257.11119
325     dtm_vertical.depth_aw3d30.nasadem.100m_m_30m_s0..0cm_2017_v0.1.tif  244.43550
204                          dtm_twi_merit.dem_m_2km_s0..0cm_2017_v1.0.tif  240.34002
328           lcv_b04_sentinel.s2l2a_d_30m_s0..0cm_2018..2019.s12_v0.1.tif  220.24873
43          clm_lst_mod11a2.feb.daynight_m_1km_s0..0cm_2000..2017_v1.0.tif  217.36030
72             clm_lst_mod11a2.oct.night_m_1km_s0..0cm_2000..2017_v1.0.tif  210.13389
14         clm_precipitation_sm2rain.jun_m_1km_s0..0cm_2007..2018_v0.2.tif  190.70229
61              clm_lst_mod11a2.may.day_m_1km_s0..0cm_2000..2017_v1.0.tif  187.23391
290              dtm_elevation_aw3d30.nasadem_m_30m_s0..0cm_2017_v0.1.tif  179.33366
269 clm_cloud.fraction_earthenv.modis.apr_p_1km_s0..0cm_2000..2015_v1.0.tif  162.88049
185   clm_direct.irradiation_solar.atlas.kwhm2.10_m_1km_s0..0cm_2016_v1.tif  151.38205
16         clm_precipitation_sm2rain.mar_m_1km_s0..0cm_2007..2018_v0.2.tif  148.96814
73              clm_lst_mod11a2.sep.day_m_1km_s0..0cm_2000..2017_v1.0.tif  147.08369
44            clm_lst_mod11a2.feb.night_m_1km_s0..0cm_2000..2017_v1.0.tif  138.29304
320           lcv_b09_sentinel.s2l2a_d_30m_s0..0cm_2018..2019.s12_v0.1.tif  137.23427
319           lcv_b04_sentinel.s2l2a_d_30m_s0..0cm_2018..2019.s22_v0.1.tif  134.05703
47          clm_lst_mod11a2.jan.daynight_m_1km_s0..0cm_2000..2017_v1.0.tif  133.09698
53              clm_lst_mod11a2.jun.day_m_1km_s0..0cm_2000..2017_v1.0.tif  132.39539
235                        dtm_vrm_merit.dem_m_250m_s0..0cm_2018_v1.0.tif  131.96077
20         clm_precipitation_sm2rain.nov_m_1km_s0..0cm_2007..2018_v0.2.tif  129.11108
330           lcv_b8a_sentinel.s2l2a_d_30m_s0..0cm_2018..2019.s12_v0.1.tif  121.26827
164             clm_bioclim.var_chelsa.3_m_1km_s0..0cm_1979..2013_v1.0.tif  118.03946
234                        dtm_vbf_merit.dem_m_250m_s0..0cm_2017_v1.0.tif  117.43699
273 clm_cloud.fraction_earthenv.modis.jan_p_1km_s0..0cm_2000..2015_v1.0.tif  113.74181
49              clm_lst_mod11a2.jul.day_m_1km_s0..0cm_2000..2017_v1.0.tif  112.89753
260        clm_wind.speed_terraclimate.jan_m_5km_s0..0cm_1998..2018_v1.tif  107.82618
167             clm_bioclim.var_chelsa.6_m_1km_s0..0cm_1979..2013_v1.0.tif  105.85939
71          clm_lst_mod11a2.oct.daynight_m_1km_s0..0cm_2000..2017_v1.0.tif  104.07157
142           veg_fapar_proba.v.may_u.975_250m_s0..0cm_2014..2019_v1.0.tif  103.11904
91              lcv_surf.refl.b05_mod09a1.pc3_m_500m_s0..0cm_2001_v1.0.tif  101.13754
159            clm_bioclim.var_chelsa.13_m_1km_s0..0cm_1979..2013_v1.0.tif   99.54664
324           dtm_saga.twi_aw3d30.nasadem.100m_m_30m_s0..0cm_2017_v0.1.tif   99.02696
112           veg_fapar_proba.v.apr_u.975_250m_s0..0cm_2014..2019_v1.0.tif   97.38614
285            lcv_landsat.red_wri.forestwatch_m_30m_s0..0cm_2018_v1.0.tif   97.29888
66              clm_lst_mod11a2.nov.day_sd_1km_s0..0cm_2000..2017_v1.0.tif   95.00835
```

Results of ensemble model fitting 'ranger', 'xgboost', 'glmnet', 'deepnet':

```
Variable: log.ecec.f
R-square: 0.754
Fitted values sd: 0.729
RMSE: 0.417

Random forest model:
Call:
stats::lm(formula = f, data = d)

Residuals:
    Min      1Q  Median      3Q     Max 
-3.2877 -0.1888  0.0097  0.2023  3.1494 

Coefficients:
               Estimate Std. Error t value Pr(>|t|)    
(Intercept)    2.807991   1.806781   1.554   0.1202    
regr.ranger    1.046105   0.004845 215.911  < 2e-16 ***
regr.xgboost  -0.016558   0.005912  -2.801   0.0051 ** 
regr.cubist    0.031843   0.005063   6.289 3.21e-10 ***
regr.nnet     -1.142820   0.713071  -1.603   0.1090    
regr.cvglmnet -0.027630   0.005607  -4.928 8.34e-07 ***
---
Signif. codes:  0 ‘***’ 0.001 ‘**’ 0.01 ‘*’ 0.05 ‘.’ 0.1 ‘ ’ 1

Residual standard error: 0.4166 on 66380 degrees of freedom
Multiple R-squared:  0.7538,    Adjusted R-squared:  0.7538 
F-statistic: 4.065e+04 on 5 and 66380 DF,  p-value: < 2.2e-16


Variable importance:
                                                                   variable importance
336                                                               hzn_depth  2942.4846
83                lcv_surf.refl.b02_mod09a1.pc1_m_500m_s0..0cm_2001_v1.0.tif  1793.2962
31            clm_lst_mod11a2.apr.daynight_m_1km_s0..0cm_2000..2017_v1.0.tif  1236.6638
47            clm_lst_mod11a2.jan.daynight_m_1km_s0..0cm_2000..2017_v1.0.tif   960.4174
183                                                       af_lithology_X19   941.4197
325        dtm_vertical.depth_aw3d30.nasadem.100m_m_30m_s0..0cm_2017_v0.1.tif   896.4531
165               clm_bioclim.var_chelsa.4_m_1km_s0..0cm_1979..2013_v1.0.tif   669.6200
273    clm_cloud.fraction_earthenv.modis.jan_p_1km_s0..0cm_2000..2015_v1.0.tif   626.1853
41               clm_lst_mod11a2.feb.day_m_1km_s0..0cm_2000..2017_v1.0.tif   550.5576
95                lcv_surf.refl.b06_mod09a1.pc1_m_500m_s0..0cm_2001_v1.0.tif   496.8094
33               clm_lst_mod11a2.aug.day_m_1km_s0..0cm_2000..2017_v1.0.tif   470.9722
30              clm_lst_mod11a2.apr.day_sd_1km_s0..0cm_2000..2017_v1.0.tif   466.6186
40             clm_lst_mod11a2.dec.night_m_1km_s0..0cm_2000..2017_v1.0.tif   447.8735
262           clm_wind.speed_terraclimate.jun_m_5km_s0..0cm_1998..2018_v1.tif   443.6185
274    clm_cloud.fraction_earthenv.modis.jul_p_1km_s0..0cm_2000..2015_v1.0.tif   442.9051
```

```
24            clm_precipitation_sm2rain.sep_m_1km_s0..0cm_2007..2018_v0.2.tif   421.2403
271    clm_cloud.fraction_earthenv.modis.dec_p_1km_s0..0cm_2000..2015_v1.0.tif   419.7370
227              dtm_rough.magnitude_merit.dem_m_250m_s0..0cm_2018_v1.0.tif   409.7946
320             lcv_b09_sentinel.s2l2a_d_30m_s0..0cm_2018..2019.s12_v0.1.tif   404.2286
168              clm_bioclim.var_chelsa.7_m_1km_s0..0cm_1979..2013_v1.0.tif   373.1247
20            clm_precipitation_sm2rain.nov_m_1km_s0..0cm_2007..2018_v0.2.tif   361.7942
290                dtm_elevation_aw3d30.nasadem_m_30m_s0..0cm_2017_v0.1.tif   358.2872
141                 veg_fapar_proba.v.may_r_250m_s0..0cm_2014..2019_v1.0.tif   356.5329
53                 clm_lst_mod11a2.jun.day_m_1km_s0..0cm_2000..2017_v1.0.tif   355.9928
221                     dtm_elevation_merit.dem_m_250m_s0..0cm_2017_v1.0.tif   351.8851
50               clm_lst_mod11a2.jul.day_sd_1km_s0..0cm_2000..2017_v1.0.tif   344.7256
12            clm_precipitation_sm2rain.jul_m_1km_s0..0cm_2007..2018_v0.2.tif   344.6858
257           clm_wind.speed_terraclimate.aug_m_5km_s0..0cm_1998..2018_v1.tif   329.6783
56               clm_lst_mod11a2.jun.night_m_1km_s0..0cm_2000..2017_v1.0.tif   328.7805
38               clm_lst_mod11a2.dec.day_sd_1km_s0..0cm_2000..2017_v1.0.tif   327.6904
48               clm_lst_mod11a2.jan.night_m_1km_s0..0cm_2000..2017_v1.0.tif   324.0546
71            clm_lst_mod11a2.oct.daynight_m_1km_s0..0cm_2000..2017_v1.0.tif   313.1764
279    clm_cloud.fraction_earthenv.modis.oct_p_1km_s0..0cm_2000..2015_v1.0.tif   302.3604
298             lcv_b09_sentinel.s2l2a_d_30m_s0..0cm_2018..2019.s22_v0.1.tif   302.2950
1          clm_precipitation_sm2rain.annual_m_1km_s0..0cm_2007..2018_v0.2.tif   301.6377
26              clm_lst_mod11a2.annual.day_m_1km_s0..0cm_2000..2017_v1.0.tif   298.1593
280    clm_cloud.fraction_earthenv.modis.sep_p_1km_s0..0cm_2000..2015_v1.0.tif   297.2237
309               lcv_landsat.nir_wri.forestwatch_m_30m_s0..0cm_2000_v1.0.tif   293.0423
157             clm_bioclim.var_chelsa.11_m_1km_s0..0cm_1979..2013_v1.0.tif   291.2268
8             clm_precipitation_sm2rain.feb_m_1km_s0..0cm_2007..2018_v0.2.tif   287.2366
74               clm_lst_mod11a2.sep.day_sd_1km_s0..0cm_2000..2017_v1.0.tif   286.0513
35            clm_lst_mod11a2.aug.daynight_m_1km_s0..0cm_2000..2017_v1.0.tif   281.2668
62               clm_lst_mod11a2.may.day_sd_1km_s0..0cm_2000..2017_v1.0.tif   281.0670
268 clm_cloud.fraction_earthenv.modis.annual_m_1km_s0..0cm_2000..2015_v1.0.tif   280.9722
190                            dtm_dvm_merit.dem_m_1km_s0..0cm_2017_v1.0.tif   280.0835
256           clm_wind.speed_terraclimate.apr_m_5km_s0..0cm_1998..2018_v1.tif   275.7936
137                 veg_fapar_proba.v.mar_r_250m_s0..0cm_2014..2019_v1.0.tif   274.6347
45                 clm_lst_mod11a2.jan.day_m_1km_s0..0cm_2000..2017_v1.0.tif   273.4807
90               lcv_surf.refl.b05_mod09a1.pc2_m_500m_s0..0cm_2001_v1.0.tif   271.0436
191                            dtm_dvm_merit.dem_m_2km_s0..0cm_2017_v1.0.tif   268.0071

Results of ensemble model fitting 'ranger', 'xgboost', 'glmnet', 'deepnet':

Variable: log.fe_mehlich3
R-square: 0.817
Fitted values sd: 0.497
RMSE: 0.235

Random forest model:
Call:
stats::lm(formula = f, data = d)
```

```
Residuals:
    Min      1Q  Median      3Q     Max 
-4.0165 -0.1312 -0.0082  0.1238  2.5077 

Coefficients:
               Estimate Std. Error t value Pr(>|t|)    
(Intercept)    3.913522   1.869721   2.093 0.036344 *  
regr.ranger    0.856893   0.007912 108.306  < 2e-16 ***
regr.xgboost   0.027856   0.007738   3.600 0.000318 ***
regr.cubist    0.146095   0.007230  20.207  < 2e-16 ***
regr.nnet     -0.879348   0.402810  -2.183 0.029037 *  
regr.cvglmnet  0.005610   0.004470   1.255 0.209415    
---
Signif. codes:  0 ‘***’ 0.001 ‘**’ 0.01 ‘*’ 0.05 ‘.’ 0.1 ‘ ’ 1

Residual standard error: 0.2349 on 57526 degrees of freedom
Multiple R-squared:  0.8173,    Adjusted R-squared:  0.8173 
F-statistic: 5.148e+04 on 5 and 57526 DF,  p-value: < 2.2e-16


Variable importance:
                                                                   variable importance
336                                                               hzn_depth 4211.56365
1           clm_precipitation_sm2rain.annual_m_1km_s0..0cm_2007..2018_v0.2.tif  751.76482
278    clm_cloud.fraction_earthenv.modis.nov_p_1km_s0..0cm_2000..2015_v1.0.tif  504.21235
168                 clm_bioclim.var_chelsa.7_m_1km_s0..0cm_1979..2013_v1.0.tif  376.24932
57                   clm_lst_mod11a2.mar.day_m_1km_s0..0cm_2000..2017_v1.0.tif  358.44514
101                 lcv_surf.refl.b07_mod09a1.pc1_m_500m_s0..0cm_2001_v1.0.tif  310.04292
162                clm_bioclim.var_chelsa.17_m_1km_s0..0cm_1979..2013_v1.0.tif  256.75292
31              clm_lst_mod11a2.apr.daynight_m_1km_s0..0cm_2000..2017_v1.0.tif  247.92123
268 clm_cloud.fraction_earthenv.modis.annual_m_1km_s0..0cm_2000..2015_v1.0.tif  246.86960
20             clm_precipitation_sm2rain.nov_m_1km_s0..0cm_2007..2018_v0.2.tif  202.62456
24             clm_precipitation_sm2rain.sep_m_1km_s0..0cm_2007..2018_v0.2.tif  201.88177
75              clm_lst_mod11a2.sep.daynight_m_1km_s0..0cm_2000..2017_v1.0.tif  189.18492
95                  lcv_surf.refl.b06_mod09a1.pc1_m_500m_s0..0cm_2001_v1.0.tif  188.38100
89                  lcv_surf.refl.b05_mod09a1.pc1_m_500m_s0..0cm_2001_v1.0.tif  180.13546
185     clm_direct.irradiation_solar.atlas.kwhm2.10_m_1km_s0..0cm_2016_v1.tif  174.75047
148               veg_fapar_proba.v.oct_l.025_250m_s0..0cm_2014..2019_v1.0.tif  164.16878
132               veg_fapar_proba.v.jun_l.025_250m_s0..0cm_2014..2019_v1.0.tif  162.79290
263            clm_wind.speed_terraclimate.mar_m_5km_s0..0cm_1998..2018_v1.tif  161.41109
10             clm_precipitation_sm2rain.jan_m_1km_s0..0cm_2007..2018_v0.2.tif  158.69588
325        dtm_vertical.depth_aw3d30.nasadem.100m_m_30m_s0..0cm_2017_v0.1.tif  153.83944
15       clm_precipitation_sm2rain.jun_sd.10_10km_s0..0cm_2007..2018_v1.0.tif  148.38200
290                   dtm_elevation_aw3d30.nasadem_m_30m_s0..0cm_2017_v0.1.tif  132.38091
166                 clm_bioclim.var_chelsa.5_m_1km_s0..0cm_1979..2013_v1.0.tif  129.85375
```

```
322            lcv_landsat.swir2_wri.forestwatch_m_30m_s0..0cm_2018_v1.0.tif  129.46999
65                 clm_lst_mod11a2.nov.day_m_1km_s0..0cm_2000..2017_v1.0.tif  127.10799
271    clm_cloud.fraction_earthenv.modis.dec_p_1km_s0..0cm_2000..2015_v1.0.tif  126.44015
67            clm_lst_mod11a2.nov.daynight_m_1km_s0..0cm_2000..2017_v1.0.tif  125.22611
323                dtm_openn_aw3d30.nasadem.100m_m_30m_s0..0cm_2017_v0.1.tif  124.85134
277    clm_cloud.fraction_earthenv.modis.may_p_1km_s0..0cm_2000..2015_v1.0.tif  123.93251
267          clm_wind.speed_terraclimate.sep_m_5km_s0..0cm_1998..2018_v1.tif  113.74646
83                lcv_surf.refl.b02_mod09a1.pc1_m_500m_s0..0cm_2001_v1.0.tif  111.80516
56              clm_lst_mod11a2.jun.night_m_1km_s0..0cm_2000..2017_v1.0.tif  108.09862
11       clm_precipitation_sm2rain.jan_sd.10_10km_s0..0cm_2007..2018_v1.0.tif  105.14812
14          clm_precipitation_sm2rain.jun_m_1km_s0..0cm_2007..2018_v0.2.tif  104.95026
209                           dtm_vbf_merit.dem_m_2km_s0..0cm_2017_v1.0.tif  100.91802
221                    dtm_elevation_merit.dem_m_250m_s0..0cm_2017_v1.0.tif   97.16541
29                 clm_lst_mod11a2.apr.day_m_1km_s0..0cm_2000..2017_v1.0.tif   94.70764
275    clm_cloud.fraction_earthenv.modis.jun_p_1km_s0..0cm_2000..2015_v1.0.tif   94.19590
12          clm_precipitation_sm2rain.jul_m_1km_s0..0cm_2007..2018_v0.2.tif   92.08210
54              clm_lst_mod11a2.jun.day_sd_1km_s0..0cm_2000..2017_v1.0.tif   91.45380
6           clm_precipitation_sm2rain.dec_m_1km_s0..0cm_2007..2018_v0.2.tif   90.41915
159             clm_bioclim.var_chelsa.13_m_1km_s0..0cm_1979..2013_v1.0.tif   88.14494
269    clm_cloud.fraction_earthenv.modis.apr_p_1km_s0..0cm_2000..2015_v1.0.tif   87.37166
258          clm_wind.speed_terraclimate.dec_m_5km_s0..0cm_1998..2018_v1.tif   84.74383
136            veg_fapar_proba.v.mar_l.025_250m_s0..0cm_2014..2019_v1.0.tif   83.73858
4           clm_precipitation_sm2rain.aug_m_1km_s0..0cm_2007..2018_v0.2.tif   83.16436
265          clm_wind.speed_terraclimate.nov_m_5km_s0..0cm_1998..2018_v1.tif   81.71888
51            clm_lst_mod11a2.jul.daynight_m_1km_s0..0cm_2000..2017_v1.0.tif   80.83370
3        clm_precipitation_sm2rain.apr_sd.10_10km_s0..0cm_2007..2018_v1.0.tif   80.65716
234                         dtm_vbf_merit.dem_m_250m_s0..0cm_2017_v1.0.tif   78.85146

Results of ensemble model fitting 'ranger', 'xgboost', 'glmnet', 'deepnet':

Variable: log.k_mehlich3
R-square: 0.773
Fitted values sd: 0.938
RMSE: 0.509

Random forest model:
Call:
stats::lm(formula = f, data = d)

Residuals:
    Min      1Q  Median      3Q     Max
-4.3088 -0.2648 -0.0037  0.2639  6.8136

Coefficients:
               Estimate Std. Error t value Pr(>|t|)
(Intercept)   10.907726   6.134422   1.778   0.0754 .
```

```
regr.ranger    1.004487   0.003878 259.026   <2e-16 ***
regr.xgboost  -0.004081   0.004739  -0.861   0.3892
regr.cubist    0.084556   0.004346  19.454   <2e-16 ***
regr.nnet     -2.205286   1.228586  -1.795   0.0727 .
regr.cvglmnet -0.064510   0.003933 -16.401   <2e-16 ***
---
Signif. codes:  0 ‘***’ 0.001 ‘**’ 0.01 ‘*’ 0.05 ‘.’ 0.1 ‘ ’ 1

Residual standard error: 0.5092 on 139122 degrees of freedom
Multiple R-squared:  0.7725,    Adjusted R-squared:  0.7725
F-statistic: 9.451e+04 on 5 and 139122 DF,  p-value: < 2.2e-16


Variable importance:
                                                                  variable importance
336                                                              hzn_depth 10227.3215
290                dtm_elevation_aw3d30.nasadem_m_30m_s0..0cm_2017_v0.1.tif 10018.9636
164              clm_bioclim.var_chelsa.3_m_1km_s0..0cm_1979..2013_v1.0.tif  8704.8286
209                           dtm_vbf_merit.dem_m_2km_s0..0cm_2017_v1.0.tif  6659.1114
59           clm_lst_mod11a2.mar.daynight_m_1km_s0..0cm_2000..2017_v1.0.tif  5875.7950
272 clm_cloud.fraction_earthenv.modis.feb_p_1km_s0..0cm_2000..2015_v1.0.tif  2928.5415
51           clm_lst_mod11a2.jul.daynight_m_1km_s0..0cm_2000..2017_v1.0.tif  2737.4136
35           clm_lst_mod11a2.aug.daynight_m_1km_s0..0cm_2000..2017_v1.0.tif  2663.4097
168              clm_bioclim.var_chelsa.7_m_1km_s0..0cm_1979..2013_v1.0.tif  2481.9534
46               clm_lst_mod11a2.jan.day_sd_1km_s0..0cm_2000..2017_v1.0.tif  2453.6357
22          clm_precipitation_sm2rain.oct_m_1km_s0..0cm_2007..2018_v0.2.tif  1983.4801
273 clm_cloud.fraction_earthenv.modis.jan_p_1km_s0..0cm_2000..2015_v1.0.tif  1929.7810
39           clm_lst_mod11a2.dec.daynight_m_1km_s0..0cm_2000..2017_v1.0.tif  1921.4043
64              clm_lst_mod11a2.may.night_m_1km_s0..0cm_2000..2017_v1.0.tif  1814.1560
157             clm_bioclim.var_chelsa.11_m_1km_s0..0cm_1979..2013_v1.0.tif  1768.5479
208                           dtm_vbf_merit.dem_m_1km_s0..0cm_2017_v1.0.tif  1720.0973
20          clm_precipitation_sm2rain.nov_m_1km_s0..0cm_2007..2018_v0.2.tif  1718.0333
31           clm_lst_mod11a2.apr.daynight_m_1km_s0..0cm_2000..2017_v1.0.tif  1709.1744
271 clm_cloud.fraction_earthenv.modis.dec_p_1km_s0..0cm_2000..2015_v1.0.tif  1560.8609
161             clm_bioclim.var_chelsa.16_m_1km_s0..0cm_1979..2013_v1.0.tif  1557.2605
47           clm_lst_mod11a2.jan.daynight_m_1km_s0..0cm_2000..2017_v1.0.tif  1480.3266
266         clm_wind.speed_terraclimate.oct_m_5km_s0..0cm_1998..2018_v1.tif  1402.1554
158             clm_bioclim.var_chelsa.12_m_1km_s0..0cm_1979..2013_v1.0.tif  1389.0582
185   clm_direct.irradiation_solar.atlas.kwhm2.10_m_1km_s0..0cm_2016_v1.tif  1344.7656
57                clm_lst_mod11a2.mar.day_m_1km_s0..0cm_2000..2017_v1.0.tif  1297.0359
227              dtm_rough.magnitude_merit.dem_m_250m_s0..0cm_2018_v1.0.tif  1296.6207
10          clm_precipitation_sm2rain.jan_m_1km_s0..0cm_2007..2018_v0.2.tif  1259.5051
278 clm_cloud.fraction_earthenv.modis.nov_p_1km_s0..0cm_2000..2015_v1.0.tif  1235.1713
325     dtm_vertical.depth_aw3d30.nasadem.100m_m_30m_s0..0cm_2017_v0.1.tif  1222.5705
8           clm_precipitation_sm2rain.feb_m_1km_s0..0cm_2007..2018_v0.2.tif  1192.1016
274 clm_cloud.fraction_earthenv.modis.jul_p_1km_s0..0cm_2000..2015_v1.0.tif  1178.0660
```

```
162            clm_bioclim.var_chelsa.17_m_1km_s0..0cm_1979..2013_v1.0.tif  1139.1934
89              lcv_surf.refl.b05_mod09a1.pc1_m_500m_s0..0cm_2001_v1.0.tif  1101.3717
33               clm_lst_mod11a2.aug.day_m_1km_s0..0cm_2000..2017_v1.0.tif  1097.1118
3      clm_precipitation_sm2rain.apr_sd.10_10km_s0..0cm_2007..2018_v1.0.tif  1054.9025
84              lcv_surf.refl.b02_mod09a1.pc2_m_500m_s0..0cm_2001_v1.0.tif  1049.9744
258        clm_wind.speed_terraclimate.dec_m_5km_s0..0cm_1998..2018_v1.tif  1039.3554
110           veg_fapar_proba.v.apr_l.025_250m_s0..0cm_2014..2019_v1.0.tif  1003.1885
5      clm_precipitation_sm2rain.aug_sd.10_10km_s0..0cm_2007..2018_v1.0.tif   995.1651
257        clm_wind.speed_terraclimate.aug_m_5km_s0..0cm_1998..2018_v1.tif   993.6640
14         clm_precipitation_sm2rain.jun_m_1km_s0..0cm_2007..2018_v0.2.tif   962.4582
136           veg_fapar_proba.v.mar_l.025_250m_s0..0cm_2014..2019_v1.0.tif   948.6746
270 clm_cloud.fraction_earthenv.modis.aug_p_1km_s0..0cm_2000..2015_v1.0.tif   940.9086
113               veg_fapar_proba.v.aug_d_250m_s0..0cm_2014..2019_v1.0.tif   888.3852
269 clm_cloud.fraction_earthenv.modis.apr_p_1km_s0..0cm_2000..2015_v1.0.tif   846.8952
53               clm_lst_mod11a2.jun.day_m_1km_s0..0cm_2000..2017_v1.0.tif   843.2879
74              clm_lst_mod11a2.sep.day_sd_1km_s0..0cm_2000..2017_v1.0.tif   835.6056
149               veg_fapar_proba.v.oct_r_250m_s0..0cm_2014..2019_v1.0.tif   833.4164
18         clm_precipitation_sm2rain.may_m_1km_s0..0cm_2007..2018_v0.2.tif   805.4782
265        clm_wind.speed_terraclimate.nov_m_5km_s0..0cm_1998..2018_v1.tif   792.1192

Results of ensemble model fitting 'ranger', 'xgboost', 'glmnet', 'deepnet':

Variable: log.mg_mehlich3
R-square: 0.815
Fitted values sd: 1.05
RMSE: 0.498

Random forest model:
Call:
stats::lm(formula = f, data = d)

Residuals:
    Min      1Q  Median      3Q     Max
-5.8775 -0.2312  0.0028  0.2465  3.7400

Coefficients:
               Estimate Std. Error t value Pr(>|t|)
(Intercept)   -0.034349   0.051219  -0.671   0.5025
regr.ranger    1.034217   0.003263 316.950   <2e-16 ***
regr.xgboost  -0.008057   0.003854  -2.091   0.0366 *
regr.cubist    0.073223   0.003649  20.067   <2e-16 ***
regr.nnet     -0.017388   0.009528  -1.825   0.0680 .
regr.cvglmnet -0.075566   0.003402 -22.213   <2e-16 ***
---
Signif. codes:  0 ‘***’ 0.001 ‘**’ 0.01 ‘*’ 0.05 ‘.’ 0.1 ‘ ’ 1
```

```
Residual standard error: 0.4979 on 136681 degrees of freedom
Multiple R-squared:  0.8152,    Adjusted R-squared:  0.8152
F-statistic: 1.206e+05 on 5 and 136681 DF,  p-value: < 2.2e-16


Variable importance:
                                                                   variable importance
89                 lcv_surf.refl.b05_mod09a1.pc1_m_500m_s0..0cm_2001_v1.0.tif 11658.0891
164                clm_bioclim.var_chelsa.3_m_1km_s0..0cm_1979..2013_v1.0.tif 10809.6652
272    clm_cloud.fraction_earthenv.modis.feb_p_1km_s0..0cm_2000..2015_v1.0.tif  9517.4497
336                                                                  hzn_depth  9356.2670
43             clm_lst_mod11a2.feb.daynight_m_1km_s0..0cm_2000..2017_v1.0.tif  8421.4743
47             clm_lst_mod11a2.jan.daynight_m_1km_s0..0cm_2000..2017_v1.0.tif  3063.8104
1          clm_precipitation_sm2rain.annual_m_1km_s0..0cm_2007..2018_v0.2.tif  2752.7731
221                  dtm_elevation_merit.dem_m_250m_s0..0cm_2017_v1.0.tif  2646.6504
208                         dtm_vbf_merit.dem_m_1km_s0..0cm_2017_v1.0.tif  2621.2727
325        dtm_vertical.depth_aw3d30.nasadem.100m_m_30m_s0..0cm_2017_v0.1.tif  2569.9765
37                 clm_lst_mod11a2.dec.day_m_1km_s0..0cm_2000..2017_v1.0.tif  2567.4405
275    clm_cloud.fraction_earthenv.modis.jun_p_1km_s0..0cm_2000..2015_v1.0.tif  2286.1967
125             veg_fapar_proba.v.jan_l.025_250m_s0..0cm_2014..2019_v1.0.tif  2132.7690
165               clm_bioclim.var_chelsa.4_m_1km_s0..0cm_1979..2013_v1.0.tif  2001.6765
264          clm_wind.speed_terraclimate.may_m_5km_s0..0cm_1998..2018_v1.tif  1922.4882
158              clm_bioclim.var_chelsa.12_m_1km_s0..0cm_1979..2013_v1.0.tif  1847.0113
227              dtm_rough.magnitude_merit.dem_m_250m_s0..0cm_2018_v1.0.tif  1834.8276
18           clm_precipitation_sm2rain.may_m_1km_s0..0cm_2007..2018_v0.2.tif  1756.2352
210                        dtm_vbf_merit.dem_m_500m_s0..0cm_2017_v1.0.tif  1705.8147
24           clm_precipitation_sm2rain.sep_m_1km_s0..0cm_2007..2018_v0.2.tif  1684.7982
271    clm_cloud.fraction_earthenv.modis.dec_p_1km_s0..0cm_2000..2015_v1.0.tif  1656.0278
256          clm_wind.speed_terraclimate.apr_m_5km_s0..0cm_1998..2018_v1.tif  1545.6106
110             veg_fapar_proba.v.apr_l.025_250m_s0..0cm_2014..2019_v1.0.tif  1539.1636
19       clm_precipitation_sm2rain.may_sd.10_10km_s0..0cm_2007..2018_v1.0.tif  1525.1769
209                         dtm_vbf_merit.dem_m_2km_s0..0cm_2017_v1.0.tif  1444.3785
84                lcv_surf.refl.b02_mod09a1.pc2_m_500m_s0..0cm_2001_v1.0.tif  1415.8759
257          clm_wind.speed_terraclimate.aug_m_5km_s0..0cm_1998..2018_v1.tif  1401.6521
49                 clm_lst_mod11a2.jul.day_m_1km_s0..0cm_2000..2017_v1.0.tif  1401.0751
30                clm_lst_mod11a2.apr.day_sd_1km_s0..0cm_2000..2017_v1.0.tif  1384.8821
35            clm_lst_mod11a2.aug.daynight_m_1km_s0..0cm_2000..2017_v1.0.tif  1379.8090
259          clm_wind.speed_terraclimate.feb_m_5km_s0..0cm_1998..2018_v1.tif  1335.2672
26              clm_lst_mod11a2.annual.day_m_1km_s0..0cm_2000..2017_v1.0.tif  1316.1492
163               clm_bioclim.var_chelsa.2_m_1km_s0..0cm_1979..2013_v1.0.tif  1200.6009
4            clm_precipitation_sm2rain.aug_m_1km_s0..0cm_2007..2018_v0.2.tif  1181.9259
2            clm_precipitation_sm2rain.apr_m_1km_s0..0cm_2007..2018_v0.2.tif  1162.6065
263          clm_wind.speed_terraclimate.mar_m_5km_s0..0cm_1998..2018_v1.tif  1146.3949
268 clm_cloud.fraction_earthenv.modis.annual_m_1km_s0..0cm_2000..2015_v1.0.tif  1145.6780
300             lcv_b11_sentinel.s2l2a_d_30m_s0..0cm_2018..2019.s12_v0.1.tif  1135.2428
39            clm_lst_mod11a2.dec.daynight_m_1km_s0..0cm_2000..2017_v1.0.tif  1122.1417
```

```
286             lcv_landsat.swir1_wri.forestwatch_m_30m_s0..0cm_2000_v1.0.tif  1122.1017
157               clm_bioclim.var_chelsa.11_m_1km_s0..0cm_1979..2013_v1.0.tif  1096.7862
66                 clm_lst_mod11a2.nov.day_sd_1km_s0..0cm_2000..2017_v1.0.tif  1081.7642
8             clm_precipitation_sm2rain.feb_m_1km_s0..0cm_2007..2018_v0.2.tif  1079.2317
266           clm_wind.speed_terraclimate.oct_m_5km_s0..0cm_1998..2018_v1.tif  1040.0766
108              veg_fapar_proba.v.annnual_d_250m_s0..0cm_2014..2019_v1.0.tif  1015.6935
277    clm_cloud.fraction_earthenv.modis.may_p_1km_s0..0cm_2000..2015_v1.0.tif   990.2735
34                 clm_lst_mod11a2.aug.day_sd_1km_s0..0cm_2000..2017_v1.0.tif   969.9446
135                  veg_fapar_proba.v.mar_d_250m_s0..0cm_2014..2019_v1.0.tif   968.3554
229                      dtm_roughness_merit.dem_m_250m_s0..0cm_2018_v1.0.tif   965.1066
111                  veg_fapar_proba.v.apr_r_250m_s0..0cm_2014..2019_v1.0.tif   962.7918

Results of ensemble model fitting 'ranger', 'xgboost', 'glmnet', 'deepnet':

Variable: log.n_tot_ncs
R-square: 0.732
Fitted values sd: 0.326
RMSE: 0.197

Random forest model:
Call:
stats::lm(formula = f, data = d)

Residuals:
     Min       1Q   Median       3Q      Max
-1.87298 -0.09584 -0.00985  0.07613  3.14728

Coefficients:
               Estimate Std. Error t value Pr(>|t|)
(Intercept)    0.267429   0.493235   0.542    0.588
regr.ranger    1.128208   0.005766 195.669  < 2e-16 ***
regr.xgboost  -0.048780   0.006108  -7.987  1.4e-15 ***
regr.cubist    0.143954   0.004424  32.539  < 2e-16 ***
regr.nnet     -0.482261   0.797938  -0.604    0.546
regr.cvglmnet -0.170889   0.004955 -34.489  < 2e-16 ***
---
Signif. codes:  0 '***' 0.001 '**' 0.01 '*' 0.05 '.' 0.1 ' ' 1

Residual standard error: 0.1972 on 99249 degrees of freedom
Multiple R-squared:  0.7319,    Adjusted R-squared:  0.7319
F-statistic: 5.419e+04 on 5 and 99249 DF,  p-value: < 2.2e-16


Variable importance:
                                                                      variable importance
336                                                                 hzn_depth 2148.70938
```

227	dtm_rough.magnitude_merit.dem_m_250m_s0..0cm_2018_v1.0.tif	864.35073
65	clm_lst_mod11a2.nov.day_m_1km_s0..0cm_2000..2017_v1.0.tif	539.88155
1	clm_precipitation_sm2rain.annual_m_1km_s0..0cm_2007..2018_v0.2.tif	237.76542
101	lcv_surf.refl.b07_mod09a1.pc1_m_500m_s0..0cm_2001_v1.0.tif	236.14521
301	lcv_b11_sentinel.s2l2a_d_30m_s0..0cm_2018..2019.s22_v0.1.tif	224.39376
235	dtm_vrm_merit.dem_m_250m_s0..0cm_2018_v1.0.tif	218.06073
95	lcv_surf.refl.b06_mod09a1.pc1_m_500m_s0..0cm_2001_v1.0.tif	187.84097
89	lcv_surf.refl.b05_mod09a1.pc1_m_500m_s0..0cm_2001_v1.0.tif	169.72542
61	clm_lst_mod11a2.may.day_m_1km_s0..0cm_2000..2017_v1.0.tif	164.43068
14	clm_precipitation_sm2rain.jun_m_1km_s0..0cm_2007..2018_v0.2.tif	147.98790
286	lcv_landsat.swir1_wri.forestwatch_m_30m_s0..0cm_2000_v1.0.tif	147.26162
275	clm_cloud.fraction_earthenv.modis.jun_p_1km_s0..0cm_2000..2015_v1.0.tif	139.63193
18	clm_precipitation_sm2rain.may_m_1km_s0..0cm_2007..2018_v0.2.tif	133.40604
68	clm_lst_mod11a2.nov.night_m_1km_s0..0cm_2000..2017_v1.0.tif	121.11814
229	dtm_roughness_merit.dem_m_250m_s0..0cm_2018_v1.0.tif	111.78102
325	dtm_vertical.depth_aw3d30.nasadem.100m_m_30m_s0..0cm_2017_v0.1.tif	109.69198
164	clm_bioclim.var_chelsa.3_m_1km_s0..0cm_1979..2013_v1.0.tif	107.32010
166	clm_bioclim.var_chelsa.5_m_1km_s0..0cm_1979..2013_v1.0.tif	107.14063
162	clm_bioclim.var_chelsa.17_m_1km_s0..0cm_1979..2013_v1.0.tif	105.83956
37	clm_lst_mod11a2.dec.day_m_1km_s0..0cm_2000..2017_v1.0.tif	104.37362
83	lcv_surf.refl.b02_mod09a1.pc1_m_500m_s0..0cm_2001_v1.0.tif	97.85928
268	clm_cloud.fraction_earthenv.modis.annual_m_1km_s0..0cm_2000..2015_v1.0.tif	97.23733
142	veg_fapar_proba.v.may_u.975_250m_s0..0cm_2014..2019_v1.0.tif	92.20478
269	clm_cloud.fraction_earthenv.modis.apr_p_1km_s0..0cm_2000..2015_v1.0.tif	89.18928
209	dtm_vbf_merit.dem_m_2km_s0..0cm_2017_v1.0.tif	87.64995
63	clm_lst_mod11a2.may.daynight_m_1km_s0..0cm_2000..2017_v1.0.tif	86.56225
320	lcv_b09_sentinel.s2l2a_d_30m_s0..0cm_2018..2019.s12_v0.1.tif	85.65804
270	clm_cloud.fraction_earthenv.modis.aug_p_1km_s0..0cm_2000..2015_v1.0.tif	84.07678
29	clm_lst_mod11a2.apr.day_m_1km_s0..0cm_2000..2017_v1.0.tif	83.38562
332	lcv_b12_sentinel.s2l2a_d_30m_s0..0cm_2018..2019.s22_v0.1.tif	82.88817
139	veg_fapar_proba.v.may_d_250m_s0..0cm_2014..2019_v1.0.tif	82.13841
4	clm_precipitation_sm2rain.aug_m_1km_s0..0cm_2007..2018_v0.2.tif	79.65647
44	clm_lst_mod11a2.feb.night_m_1km_s0..0cm_2000..2017_v1.0.tif	79.35033
168	clm_bioclim.var_chelsa.7_m_1km_s0..0cm_1979..2013_v1.0.tif	78.93093
58	clm_lst_mod11a2.mar.day_sd_1km_s0..0cm_2000..2017_v1.0.tif	78.82087
276	clm_cloud.fraction_earthenv.modis.mar_p_1km_s0..0cm_2000..2015_v1.0.tif	78.61886
204	dtm_twi_merit.dem_m_2km_s0..0cm_2017_v1.0.tif	77.71265
66	clm_lst_mod11a2.nov.day_sd_1km_s0..0cm_2000..2017_v1.0.tif	77.57004
272	clm_cloud.fraction_earthenv.modis.feb_p_1km_s0..0cm_2000..2015_v1.0.tif	77.43724
273	clm_cloud.fraction_earthenv.modis.jan_p_1km_s0..0cm_2000..2015_v1.0.tif	77.37877
47	clm_lst_mod11a2.jan.daynight_m_1km_s0..0cm_2000..2017_v1.0.tif	76.43363
67	clm_lst_mod11a2.nov.daynight_m_1km_s0..0cm_2000..2017_v1.0.tif	74.87560
158	clm_bioclim.var_chelsa.12_m_1km_s0..0cm_1979..2013_v1.0.tif	73.29149
210	dtm_vbf_merit.dem_m_500m_s0..0cm_2017_v1.0.tif	73.17131
184	clm_diffuse.irradiation_solar.atlas.kwhm2.100_m_1km_s0..0cm_2016_v1.tif	72.92421
42	clm_lst_mod11a2.feb.day_sd_1km_s0..0cm_2000..2017_v1.0.tif	72.82393

```
12             clm_precipitation_sm2rain.jul_m_1km_s0..0cm_2007..2018_v0.2.tif   70.72952
10             clm_precipitation_sm2rain.jan_m_1km_s0..0cm_2007..2018_v0.2.tif   70.18299
161                clm_bioclim.var_chelsa.16_m_1km_s0..0cm_1979..2013_v1.0.tif   69.01352

Results of ensemble model fitting 'ranger', 'xgboost', 'glmnet', 'deepnet':

Variable: log.oc
R-square: 0.791
Fitted values sd: 0.716
RMSE: 0.369

Random forest model:
Call:
stats::lm(formula = f, data = d)

Residuals:
    Min      1Q  Median      3Q     Max
-3.1517 -0.1900 -0.0060  0.1793  4.2621

Coefficients:
               Estimate Std. Error t value Pr(>|t|)
(Intercept)    1.821657   0.794000   2.294   0.0218 *
regr.ranger    1.047507   0.005146 203.571   <2e-16 ***
regr.xgboost  -0.005943   0.005340  -1.113   0.2657
regr.cubist    0.052084   0.004884  10.664   <2e-16 ***
regr.nnet     -0.867384   0.359213  -2.415   0.0158 *
regr.cvglmnet -0.050157   0.003863 -12.984   <2e-16 ***
---
Signif. codes:  0 ‘***’ 0.001 ‘**’ 0.01 ‘*’ 0.05 ‘.’ 0.1 ‘ ’ 1

Residual standard error: 0.3687 on 122457 degrees of freedom
Multiple R-squared:  0.7906,    Adjusted R-squared:  0.7906
F-statistic: 9.248e+04 on 5 and 122457 DF,  p-value: < 2.2e-16


Variable importance:
                                                                      variable importance
336                                                                  hzn_depth 20255.8035
2              clm_precipitation_sm2rain.apr_m_1km_s0..0cm_2007..2018_v0.2.tif  6102.3811
208                              dtm_vbf_merit.dem_m_1km_s0..0cm_2017_v1.0.tif  2270.1690
72                 clm_lst_mod11a2.oct.night_m_1km_s0..0cm_2000..2017_v1.0.tif  1796.4102
56                 clm_lst_mod11a2.jun.night_m_1km_s0..0cm_2000..2017_v1.0.tif  1766.8975
68                 clm_lst_mod11a2.nov.night_m_1km_s0..0cm_2000..2017_v1.0.tif  1003.6975
89                  lcv_surf.refl.b05_mod09a1.pc1_m_500m_s0..0cm_2001_v1.0.tif   921.8340
301               lcv_b11_sentinel.s2l2a_d_30m_s0..0cm_2018..2019.s22_v0.1.tif   838.3108
290                    dtm_elevation_aw3d30.nasadem_m_30m_s0..0cm_2017_v0.1.tif   773.4460
```

```
97                 lcv_surf.refl.b06_mod09a1.pc3_m_500m_s0..0cm_2001_v1.0.tif   763.6430
48                clm_lst_mod11a2.jan.night_m_1km_s0..0cm_2000..2017_v1.0.tif   747.2118
311             lcv_landsat.swir1_wri.forestwatch_m_30m_s0..0cm_2018_v1.0.tif   741.4477
332              lcv_b12_sentinel.s2l2a_d_30m_s0..0cm_2018..2019.s22_v0.1.tif   719.6563
328              lcv_b04_sentinel.s2l2a_d_30m_s0..0cm_2018..2019.s12_v0.1.tif   704.4746
221                     dtm_elevation_merit.dem_m_250m_s0..0cm_2017_v1.0.tif   672.9509
64                clm_lst_mod11a2.may.night_m_1km_s0..0cm_2000..2017_v1.0.tif   631.9388
65                  clm_lst_mod11a2.nov.day_m_1km_s0..0cm_2000..2017_v1.0.tif   627.1609
134              veg_fapar_proba.v.jun_u.975_250m_s0..0cm_2014..2019_v1.0.tif   566.6067
268 clm_cloud.fraction_earthenv.modis.annual_m_1km_s0..0cm_2000..2015_v1.0.tif   558.9450
204                           dtm_twi_merit.dem_m_2km_s0..0cm_2017_v1.0.tif   552.8697
277    clm_cloud.fraction_earthenv.modis.may_p_1km_s0..0cm_2000..2015_v1.0.tif   552.2587
14            clm_precipitation_sm2rain.jun_m_1km_s0..0cm_2007..2018_v0.2.tif   548.4602
161               clm_bioclim.var_chelsa.16_m_1km_s0..0cm_1979..2013_v1.0.tif   526.1304
16            clm_precipitation_sm2rain.mar_m_1km_s0..0cm_2007..2018_v0.2.tif   520.9192
73                  clm_lst_mod11a2.sep.day_m_1km_s0..0cm_2000..2017_v1.0.tif   508.4019
69                  clm_lst_mod11a2.oct.day_m_1km_s0..0cm_2000..2017_v1.0.tif   489.0852
300              lcv_b11_sentinel.s2l2a_d_30m_s0..0cm_2018..2019.s12_v0.1.tif   467.1103
91                 lcv_surf.refl.b05_mod09a1.pc3_m_500m_s0..0cm_2001_v1.0.tif   464.2171
108              veg_fapar_proba.v.annnual_d_250m_s0..0cm_2014..2019_v1.0.tif   442.6062
86                 lcv_surf.refl.b02_mod09a1.pc4_m_500m_s0..0cm_2001_v1.0.tif   440.9818
275    clm_cloud.fraction_earthenv.modis.jun_p_1km_s0..0cm_2000..2015_v1.0.tif   438.5319
325         dtm_vertical.depth_aw3d30.nasadem.100m_m_30m_s0..0cm_2017_v0.1.tif   436.9871
159               clm_bioclim.var_chelsa.13_m_1km_s0..0cm_1979..2013_v1.0.tif   433.6595
22            clm_precipitation_sm2rain.oct_m_1km_s0..0cm_2007..2018_v0.2.tif   426.0610
43              clm_lst_mod11a2.feb.daynight_m_1km_s0..0cm_2000..2017_v1.0.tif   424.6914
284               lcv_landsat.red_wri.forestwatch_m_30m_s0..0cm_2000_v1.0.tif   417.5280
269    clm_cloud.fraction_earthenv.modis.apr_p_1km_s0..0cm_2000..2015_v1.0.tif   407.3196
19        clm_precipitation_sm2rain.may_sd.10_10km_s0..0cm_2007..2018_v1.0.tif   398.0410
310               lcv_landsat.red_wri.forestwatch_m_30m_s0..0cm_2015_v1.0.tif   385.1587
8             clm_precipitation_sm2rain.feb_m_1km_s0..0cm_2007..2018_v0.2.tif   382.1089
270    clm_cloud.fraction_earthenv.modis.aug_p_1km_s0..0cm_2000..2015_v1.0.tif   374.2499
21        clm_precipitation_sm2rain.nov_sd.10_10km_s0..0cm_2007..2018_v1.0.tif   373.2142
59              clm_lst_mod11a2.mar.daynight_m_1km_s0..0cm_2000..2017_v1.0.tif   363.0467
119              veg_fapar_proba.v.dec_u.975_250m_s0..0cm_2014..2019_v1.0.tif   360.5461
77                 lcv_surf.refl.b01_mod09a1.pc1_m_500m_s0..0cm_2001_v1.0.tif   357.9015
26               clm_lst_mod11a2.annual.day_m_1km_s0..0cm_2000..2017_v1.0.tif   355.8623
274    clm_cloud.fraction_earthenv.modis.jul_p_1km_s0..0cm_2000..2015_v1.0.tif   355.3579
184    clm_diffuse.irradiation_solar.atlas.kwhm2.100_m_1km_s0..0cm_2016_v1.tif   349.3912
47              clm_lst_mod11a2.jan.daynight_m_1km_s0..0cm_2000..2017_v1.0.tif   346.4775
229                     dtm_roughness_merit.dem_m_250m_s0..0cm_2018_v1.0.tif   333.8883

Results of ensemble model fitting 'ranger', 'xgboost', 'glmnet', 'deepnet':

Variable: log.p_mehlich3
R-square: 0.486
```

```
Fitted values sd: 0.687
RMSE: 0.707

Random forest model:
Call:
stats::lm(formula = f, data = d)

Residuals:
    Min      1Q  Median      3Q     Max
-3.2892 -0.3942 -0.0637  0.2614  4.9466

Coefficients:
               Estimate Std. Error t value Pr(>|t|)
(Intercept)    3.378801   3.143200   1.075    0.282
regr.ranger    0.861655   0.011099  77.631  < 2e-16 ***
regr.xgboost   0.066139   0.013091   5.052 4.38e-07 ***
regr.cubist    0.157674   0.008886  17.744  < 2e-16 ***
regr.nnet     -1.649621   1.442240  -1.144    0.253
regr.cvglmnet  0.013628   0.010407   1.310    0.190
---
Signif. codes:  0 ‘***’ 0.001 ‘**’ 0.01 ‘*’ 0.05 ‘.’ 0.1 ‘ ’ 1

Residual standard error: 0.7066 on 53493 degrees of freedom
Multiple R-squared:  0.486, Adjusted R-squared:  0.486
F-statistic: 1.012e+04 on 5 and 53493 DF,  p-value: < 2.2e-16


Variable importance:
                                                                   variable importance
14          clm_precipitation_sm2rain.jun_m_1km_s0..0cm_2007..2018_v0.2.tif  2864.4507
336                                                               hzn_depth  1302.1635
227              dtm_rough.magnitude_merit.dem_m_250m_s0..0cm_2018_v1.0.tif  1050.8439
325      dtm_vertical.depth_aw3d30.nasadem.100m_m_30m_s0..0cm_2017_v0.1.tif   972.4168
160             clm_bioclim.var_chelsa.14_m_1km_s0..0cm_1979..2013_v1.0.tif   892.9976
164              clm_bioclim.var_chelsa.3_m_1km_s0..0cm_1979..2013_v1.0.tif   884.0819
165              clm_bioclim.var_chelsa.4_m_1km_s0..0cm_1979..2013_v1.0.tif   775.3970
1        clm_precipitation_sm2rain.annual_m_1km_s0..0cm_2007..2018_v0.2.tif   718.7164
168              clm_bioclim.var_chelsa.7_m_1km_s0..0cm_1979..2013_v1.0.tif   682.3064
22          clm_precipitation_sm2rain.oct_m_1km_s0..0cm_2007..2018_v0.2.tif   550.2259
85               lcv_surf.refl.b02_mod09a1.pc3_m_500m_s0..0cm_2001_v1.0.tif   550.1456
277 clm_cloud.fraction_earthenv.modis.may_p_1km_s0..0cm_2000..2015_v1.0.tif   542.4723
274 clm_cloud.fraction_earthenv.modis.jul_p_1km_s0..0cm_2000..2015_v1.0.tif   530.8185
30               clm_lst_mod11a2.apr.day_sd_1km_s0..0cm_2000..2017_v1.0.tif   497.0550
283             lcv_landsat.nir_wri.forestwatch_m_30m_s0..0cm_2018_v1.0.tif   487.3369
31           clm_lst_mod11a2.apr.daynight_m_1km_s0..0cm_2000..2017_v1.0.tif   439.1131
329          lcv_b12_sentinel.s2l2a_iqr_30m_s0..0cm_2018..2019.s12_v0.1.tif   436.5929
```

```
184 clm_diffuse.irradiation_solar.atlas.kwhm2.100_m_1km_s0..0cm_2016_v1.tif   431.1487
27           clm_lst_mod11a2.annual.day_sd_1km_s0..0cm_2000..2017_v1.0.tif   415.0181
4          clm_precipitation_sm2rain.aug_m_1km_s0..0cm_2007..2018_v0.2.tif   388.7653
59          clm_lst_mod11a2.mar.daynight_m_1km_s0..0cm_2000..2017_v1.0.tif   388.5768
333                  veg_f02dar.hv_alos.palsar_m_30m_s0..0cm_2017_v1.0.tif   388.0191
321         lcv_b09_sentinel.s2l2a_iqr_30m_s0..0cm_2018..2019.s22_v0.1.tif   387.2811
163             clm_bioclim.var_chelsa.2_m_1km_s0..0cm_1979..2013_v1.0.tif   380.3644
39          clm_lst_mod11a2.dec.daynight_m_1km_s0..0cm_2000..2017_v1.0.tif   376.1103
229                   dtm_roughness_merit.dem_m_250m_s0..0cm_2018_v1.0.tif   375.9394
20         clm_precipitation_sm2rain.nov_m_1km_s0..0cm_2007..2018_v0.2.tif   374.3861
208                          dtm_vbf_merit.dem_m_1km_s0..0cm_2017_v1.0.tif   369.5684
281                  veg_f02dar.hh_alos.palsar_m_30m_s0..0cm_2007_v1.0.tif   368.8913
129               veg_fapar_proba.v.jul_r_250m_s0..0cm_2014..2019_v1.0.tif   364.5444
114           veg_fapar_proba.v.aug_l.025_250m_s0..0cm_2014..2019_v1.0.tif   363.7978
38              clm_lst_mod11a2.dec.day_sd_1km_s0..0cm_2000..2017_v1.0.tif   361.3844
43          clm_lst_mod11a2.feb.daynight_m_1km_s0..0cm_2000..2017_v1.0.tif   360.1045
34              clm_lst_mod11a2.aug.day_sd_1km_s0..0cm_2000..2017_v1.0.tif   357.4737
6          clm_precipitation_sm2rain.dec_m_1km_s0..0cm_2007..2018_v0.2.tif   354.1600
19    clm_precipitation_sm2rain.may_sd.10_10km_s0..0cm_2007..2018_v1.0.tif   351.1124
331            dtm_uplocal_aw3d30.nasadem.100m_m_30m_s0..0cm_2017_v0.1.tif   350.8357
192                         dtm_dvm2_merit.dem_m_1km_s0..0cm_2017_v1.0.tif   350.3820
80              lcv_surf.refl.b01_mod09a1.pc4_m_500m_s0..0cm_2001_v1.0.tif   344.6165
272 clm_cloud.fraction_earthenv.modis.feb_p_1km_s0..0cm_2000..2015_v1.0.tif   341.1547
89              lcv_surf.refl.b05_mod09a1.pc1_m_500m_s0..0cm_2001_v1.0.tif   335.4151
288            dtm_devmean_aw3d30.nasadem.100m_m_30m_s0..0cm_2017_v0.1.tif   334.3574
53               clm_lst_mod11a2.jun.day_m_1km_s0..0cm_2000..2017_v1.0.tif   332.1994
300           lcv_b11_sentinel.s2l2a_d_30m_s0..0cm_2018..2019.s12_v0.1.tif   328.5600
231                       dtm_tcurv_merit.dem_m_250m_s0..0cm_2018_v1.0.tif   327.6651
327           lcv_b8a_sentinel.s2l2a_d_30m_s0..0cm_2018..2019.s22_v0.1.tif   327.3327
313           dtm_devmean2_aw3d30.nasadem.100m_m_30m_s0..0cm_2017_v0.1.tif   326.0274
273 clm_cloud.fraction_earthenv.modis.jan_p_1km_s0..0cm_2000..2015_v1.0.tif   324.3382
191                          dtm_dvm_merit.dem_m_2km_s0..0cm_2017_v1.0.tif   321.2799
54              clm_lst_mod11a2.jun.day_sd_1km_s0..0cm_2000..2017_v1.0.tif   317.4992

Results of ensemble model fitting 'ranger', 'xgboost', 'glmnet', 'deepnet':

Variable: log.s_mehlich3
R-square: 0.548
Fitted values sd: 0.423
RMSE: 0.384

Random forest model:
Call:
stats::lm(formula = f, data = d)

Residuals:
```

```
    Min      1Q  Median      3Q     Max 
-2.5729 -0.2102 -0.0264  0.1694  5.0049 

Coefficients:
               Estimate Std. Error t value Pr(>|t|)    
(Intercept)    1.459208   4.154229   0.351    0.725    
regr.ranger    0.937179   0.016167  57.967  < 2e-16 ***
regr.xgboost   0.002587   0.016252   0.159    0.874    
regr.cubist    0.145396   0.010890  13.351  < 2e-16 ***
regr.nnet     -0.672062   1.796642  -0.374    0.708    
regr.cvglmnet -0.045157   0.011256  -4.012 6.04e-05 ***
---
Signif. codes:  0 ‘***’ 0.001 ‘**’ 0.01 ‘*’ 0.05 ‘.’ 0.1 ‘ ’ 1

Residual standard error: 0.3841 on 37530 degrees of freedom
Multiple R-squared:  0.5481,	Adjusted R-squared:  0.548 
F-statistic:  9103 on 5 and 37530 DF,  p-value: < 2.2e-16


Variable importance:
                                                                  variable importance
164             clm_bioclim.var_chelsa.3_m_1km_s0..0cm_1979..2013_v1.0.tif  690.66815
221                   dtm_elevation_merit.dem_m_250m_s0..0cm_2017_v1.0.tif  474.21199
208                          dtm_vbf_merit.dem_m_1km_s0..0cm_2017_v1.0.tif  353.13511
227             dtm_rough.magnitude_merit.dem_m_250m_s0..0cm_2018_v1.0.tif  352.87391
24         clm_precipitation_sm2rain.sep_m_1km_s0..0cm_2007..2018_v0.2.tif  331.16664
184 clm_diffuse.irradiation_solar.atlas.kwhm2.100_m_1km_s0..0cm_2016_v1.tif  234.17556
14         clm_precipitation_sm2rain.jun_m_1km_s0..0cm_2007..2018_v0.2.tif  185.07246
167             clm_bioclim.var_chelsa.6_m_1km_s0..0cm_1979..2013_v1.0.tif  177.99550
280 clm_cloud.fraction_earthenv.modis.sep_p_1km_s0..0cm_2000..2015_v1.0.tif  166.11337
37               clm_lst_mod11a2.dec.day_m_1km_s0..0cm_2000..2017_v1.0.tif  151.83940
168             clm_bioclim.var_chelsa.7_m_1km_s0..0cm_1979..2013_v1.0.tif  142.39826
158            clm_bioclim.var_chelsa.12_m_1km_s0..0cm_1979..2013_v1.0.tif  123.53111
57               clm_lst_mod11a2.mar.day_m_1km_s0..0cm_2000..2017_v1.0.tif  121.78029
33               clm_lst_mod11a2.aug.day_m_1km_s0..0cm_2000..2017_v1.0.tif  115.49266
234                         dtm_vbf_merit.dem_m_250m_s0..0cm_2017_v1.0.tif  110.30792
279 clm_cloud.fraction_earthenv.modis.oct_p_1km_s0..0cm_2000..2015_v1.0.tif  110.24695
73               clm_lst_mod11a2.sep.day_m_1km_s0..0cm_2000..2017_v1.0.tif  109.55527
270 clm_cloud.fraction_earthenv.modis.aug_p_1km_s0..0cm_2000..2015_v1.0.tif  107.29977
166             clm_bioclim.var_chelsa.5_m_1km_s0..0cm_1979..2013_v1.0.tif  103.19180
120              veg_fapar_proba.v.feb_d_250m_s0..0cm_2014..2019_v1.0.tif  102.56230
41               clm_lst_mod11a2.feb.day_m_1km_s0..0cm_2000..2017_v1.0.tif  101.60913
89              lcv_surf.refl.b05_mod09a1.pc1_m_500m_s0..0cm_2001_v1.0.tif  100.50936
19     clm_precipitation_sm2rain.may_sd.10_10km_s0..0cm_2007..2018_v1.0.tif  100.31714
320           lcv_b09_sentinel.s2l2a_d_30m_s0..0cm_2018..2019.s12_v0.1.tif   98.50520
54              clm_lst_mod11a2.jun.day_sd_1km_s0..0cm_2000..2017_v1.0.tif   98.37292
```

```
16          clm_precipitation_sm2rain.mar_m_1km_s0..0cm_2007..2018_v0.2.tif   98.29067
21     clm_precipitation_sm2rain.nov_sd.10_10km_s0..0cm_2007..2018_v1.0.tif   96.40140
95               lcv_surf.refl.b06_mod09a1.pc1_m_500m_s0..0cm_2001_v1.0.tif   93.77023
1        clm_precipitation_sm2rain.annual_m_1km_s0..0cm_2007..2018_v0.2.tif   92.84808
20          clm_precipitation_sm2rain.nov_m_1km_s0..0cm_2007..2018_v0.2.tif   89.47828
155              clm_bioclim.var_chelsa.1_m_1km_s0..0cm_1979..2013_v1.0.tif   85.37452
62               clm_lst_mod11a2.may.day_sd_1km_s0..0cm_2000..2017_v1.0.tif   85.11217
35           clm_lst_mod11a2.aug.daynight_m_1km_s0..0cm_2000..2017_v1.0.tif   82.96540
162             clm_bioclim.var_chelsa.17_m_1km_s0..0cm_1979..2013_v1.0.tif   81.74435
8           clm_precipitation_sm2rain.feb_m_1km_s0..0cm_2007..2018_v0.2.tif   79.49712
275 clm_cloud.fraction_earthenv.modis.jun_p_1km_s0..0cm_2000..2015_v1.0.tif   79.38138
163              clm_bioclim.var_chelsa.2_m_1km_s0..0cm_1979..2013_v1.0.tif   78.13930
278 clm_cloud.fraction_earthenv.modis.nov_p_1km_s0..0cm_2000..2015_v1.0.tif   77.44151
327            lcv_b8a_sentinel.s2l2a_d_30m_s0..0cm_2018..2019.s22_v0.1.tif   76.81885
58               clm_lst_mod11a2.mar.day_sd_1km_s0..0cm_2000..2017_v1.0.tif   76.76216
330            lcv_b8a_sentinel.s2l2a_d_30m_s0..0cm_2018..2019.s12_v0.1.tif   76.69275
71           clm_lst_mod11a2.oct.daynight_m_1km_s0..0cm_2000..2017_v1.0.tif   76.45822
51           clm_lst_mod11a2.jul.daynight_m_1km_s0..0cm_2000..2017_v1.0.tif   74.97020
258         clm_wind.speed_terraclimate.dec_m_5km_s0..0cm_1998..2018_v1.tif   74.82602
229                    dtm_roughness_merit.dem_m_250m_s0..0cm_2018_v1.0.tif   74.30399
123            veg_fapar_proba.v.feb_u.975_250m_s0..0cm_2014..2019_v1.0.tif   73.50763
9      clm_precipitation_sm2rain.feb_sd.10_10km_s0..0cm_2007..2018_v1.0.tif   72.74588
160             clm_bioclim.var_chelsa.14_m_1km_s0..0cm_1979..2013_v1.0.tif   72.71787
30               clm_lst_mod11a2.apr.day_sd_1km_s0..0cm_2000..2017_v1.0.tif   72.38706
281                   veg_f02dar.hh_alos.palsar_m_30m_s0..0cm_2007_v1.0.tif   71.51745

Results of ensemble model fitting 'ranger', 'xgboost', 'glmnet', 'deepnet':

Variable: log.wpg2
R-square: 0.709
Fitted values sd: 1.25
RMSE: 0.803

Random forest model:
Call:
stats::lm(formula = f, data = d)

Residuals:
    Min      1Q  Median      3Q     Max 
-4.0555 -0.3113 -0.0222  0.2378  4.5794 

Coefficients:
               Estimate Std. Error t value Pr(>|t|)    
(Intercept)   -0.008606   1.361982  -0.006    0.995    
regr.ranger    0.972265   0.004443 218.854  < 2e-16 ***
regr.xgboost   0.034649   0.006404   5.411  6.3e-08 ***
```

```
regr.cubist    0.069589   0.005229  13.308  < 2e-16 ***
regr.nnet     -0.012756   0.796535  -0.016    0.987
regr.cvglmnet -0.056645   0.005509 -10.283  < 2e-16 ***
---
Signif. codes:  0 ‘***’ 0.001 ‘**’ 0.01 ‘*’ 0.05 ‘.’ 0.1 ‘ ’ 1

Residual standard error: 0.8032 on 92785 degrees of freedom
Multiple R-squared:  0.7092,    Adjusted R-squared:  0.7092
F-statistic: 4.525e+04 on 5 and 92785 DF,  p-value: < 2.2e-16


Variable importance:
                                                                  variable importance
124                  veg_fapar_proba.v.jan_d_250m_s0..0cm_2014..2019_v1.0.tif  17444.120
336                                                                 hzn_depth  17211.898
41                  clm_lst_mod11a2.feb.day_m_1km_s0..0cm_2000..2017_v1.0.tif   7541.375
57                  clm_lst_mod11a2.mar.day_m_1km_s0..0cm_2000..2017_v1.0.tif   6102.419
162               clm_bioclim.var_chelsa.17_m_1km_s0..0cm_1979..2013_v1.0.tif   5954.180
8             clm_precipitation_sm2rain.feb_m_1km_s0..0cm_2007..2018_v0.2.tif   5196.589
208                            dtm_vbf_merit.dem_m_1km_s0..0cm_2017_v1.0.tif   2772.433
126              veg_fapar_proba.v.jan_u.975_250m_s0..0cm_2014..2019_v1.0.tif   2462.278
290                  dtm_elevation_aw3d30.nasadem_m_30m_s0..0cm_2017_v0.1.tif   2323.912
73                  clm_lst_mod11a2.sep.day_m_1km_s0..0cm_2000..2017_v1.0.tif   2100.172
184    clm_diffuse.irradiation_solar.atlas.kwhm2.100_m_1km_s0..0cm_2016_v1.tif   1976.042
29                  clm_lst_mod11a2.apr.day_m_1km_s0..0cm_2000..2017_v1.0.tif   1969.533
155                clm_bioclim.var_chelsa.1_m_1km_s0..0cm_1979..2013_v1.0.tif   1936.928
157               clm_bioclim.var_chelsa.11_m_1km_s0..0cm_1979..2013_v1.0.tif   1875.351
10            clm_precipitation_sm2rain.jan_m_1km_s0..0cm_2007..2018_v0.2.tif   1715.364
210                          dtm_vbf_merit.dem_m_500m_s0..0cm_2017_v1.0.tif   1650.927
268 clm_cloud.fraction_earthenv.modis.annual_m_1km_s0..0cm_2000..2015_v1.0.tif   1582.444
288               dtm_devmean_aw3d30.nasadem.100m_m_30m_s0..0cm_2017_v0.1.tif   1573.769
275    clm_cloud.fraction_earthenv.modis.jun_p_1km_s0..0cm_2000..2015_v1.0.tif   1566.203
39              clm_lst_mod11a2.dec.daynight_m_1km_s0..0cm_2000..2017_v1.0.tif   1528.746
274    clm_cloud.fraction_earthenv.modis.jul_p_1km_s0..0cm_2000..2015_v1.0.tif   1499.375
21       clm_precipitation_sm2rain.nov_sd.10_10km_s0..0cm_2007..2018_v1.0.tif   1452.154
191                            dtm_dvm_merit.dem_m_2km_s0..0cm_2017_v1.0.tif   1442.747
6             clm_precipitation_sm2rain.dec_m_1km_s0..0cm_2007..2018_v0.2.tif   1415.957
326             lcv_b11_sentinel.s2l2a_iqr_30m_s0..0cm_2018..2019.s22_v0.1.tif   1415.025
297             lcv_b04_sentinel.s2l2a_iqr_30m_s0..0cm_2018..2019.s22_v0.1.tif   1404.104
325        dtm_vertical.depth_aw3d30.nasadem.100m_m_30m_s0..0cm_2017_v0.1.tif   1403.599
277    clm_cloud.fraction_earthenv.modis.may_p_1km_s0..0cm_2000..2015_v1.0.tif   1367.738
71              clm_lst_mod11a2.oct.daynight_m_1km_s0..0cm_2000..2017_v1.0.tif   1335.743
190                            dtm_dvm_merit.dem_m_1km_s0..0cm_2017_v1.0.tif   1332.501
271    clm_cloud.fraction_earthenv.modis.dec_p_1km_s0..0cm_2000..2015_v1.0.tif   1311.222
74                clm_lst_mod11a2.sep.day_sd_1km_s0..0cm_2000..2017_v1.0.tif   1299.263
70                clm_lst_mod11a2.oct.day_sd_1km_s0..0cm_2000..2017_v1.0.tif   1273.064
```

```
229                        dtm_roughness_merit.dem_m_250m_s0..0cm_2018_v1.0.tif   1258.946
38                   clm_lst_mod11a2.dec.day_sd_1km_s0..0cm_2000..2017_v1.0.tif   1243.709
30                   clm_lst_mod11a2.apr.day_sd_1km_s0..0cm_2000..2017_v1.0.tif   1235.846
218                              dtm_dvm_merit.dem_m_250m_s0..0cm_2017_v1.0.tif   1227.213
156                 clm_bioclim.var_chelsa.10_m_1km_s0..0cm_1979..2013_v1.0.tif   1214.948
15          clm_precipitation_sm2rain.jun_sd.10_10km_s0..0cm_2007..2018_v1.0.tif   1212.291
321               lcv_b09_sentinel.s2l2a_iqr_30m_s0..0cm_2018..2019.s22_v0.1.tif   1201.329
90                   lcv_surf.refl.b05_mod09a1.pc2_m_500m_s0..0cm_2001_v1.0.tif   1176.983
298                 lcv_b09_sentinel.s2l2a_d_30m_s0..0cm_2018..2019.s22_v0.1.tif   1166.598
212                        dtm_aspect.sine_merit.dem_m_250m_s0..0cm_2018_v1.0.tif   1161.763
302               lcv_b11_sentinel.s2l2a_iqr_30m_s0..0cm_2018..2019.s12_v0.1.tif   1161.589
327                 lcv_b8a_sentinel.s2l2a_d_30m_s0..0cm_2018..2019.s22_v0.1.tif   1144.972
161                 clm_bioclim.var_chelsa.16_m_1km_s0..0cm_1979..2013_v1.0.tif   1136.767
323                   dtm_openn_aw3d30.nasadem.100m_m_30m_s0..0cm_2017_v0.1.tif   1136.469
228                        dtm_rough.scale_merit.dem_m_250m_s0..0cm_2018_v1.0.tif   1130.037
282                         veg_f02dar.hh_alos.palsar_m_30m_s0..0cm_2017_v1.0.tif   1113.694
333                         veg_f02dar.hv_alos.palsar_m_30m_s0..0cm_2017_v1.0.tif   1112.093

Results of ensemble model fitting 'ranger', 'xgboost', 'glmnet', 'deepnet':

Variable: log.zn_mehlich3
R-square: 0.711
Fitted values sd: 0.588
RMSE: 0.375

Random forest model:
Call:
stats::lm(formula = f, data = d)

Residuals:
    Min      1Q  Median      3Q     Max
-2.1382 -0.2038 -0.0274  0.1632  3.6353

Coefficients:
               Estimate Std. Error t value Pr(>|t|)
(Intercept)    1.836394   1.555108   1.181  0.23766
regr.ranger    0.823144   0.013982  58.871  < 2e-16 ***
regr.xgboost   0.037861   0.013644   2.775  0.00552 **
regr.cubist    0.169653   0.010091  16.813  < 2e-16 ***
regr.nnet     -1.511745   1.244933  -1.214  0.22463
regr.cvglmnet  0.009770   0.008046   1.214  0.22467
---
Signif. codes:  0 ‘***’ 0.001 ‘**’ 0.01 ‘*’ 0.05 ‘.’ 0.1 ‘ ’ 1

Residual standard error: 0.3747 on 39344 degrees of freedom
Multiple R-squared:  0.7109,    Adjusted R-squared:  0.7109
```

```
F-statistic: 1.935e+04 on 5 and 39344 DF,  p-value: < 2.2e-16


Variable importance:
                                                                  variable importance
168              clm_bioclim.var_chelsa.7_m_1km_s0..0cm_1979..2013_v1.0.tif 1771.70604
8           clm_precipitation_sm2rain.feb_m_1km_s0..0cm_2007..2018_v0.2.tif 1324.59725
165              clm_bioclim.var_chelsa.4_m_1km_s0..0cm_1979..2013_v1.0.tif  901.72362
227              dtm_rough.magnitude_merit.dem_m_250m_s0..0cm_2018_v1.0.tif  812.88909
90               lcv_surf.refl.b05_mod09a1.pc2_m_500m_s0..0cm_2001_v1.0.tif  611.32862
336                                                               hzn_depth  497.79455
108            veg_fapar_proba.v.annnual_d_250m_s0..0cm_2014..2019_v1.0.tif  425.47394
61                clm_lst_mod11a2.may.day_m_1km_s0..0cm_2000..2017_v1.0.tif  422.05961
128            veg_fapar_proba.v.jul_l.025_250m_s0..0cm_2014..2019_v1.0.tif  404.78892
208                           dtm_vbf_merit.dem_m_1km_s0..0cm_2017_v1.0.tif  375.14352
12          clm_precipitation_sm2rain.jul_m_1km_s0..0cm_2007..2018_v0.2.tif  288.80837
14          clm_precipitation_sm2rain.jun_m_1km_s0..0cm_2007..2018_v0.2.tif  282.79413
163              clm_bioclim.var_chelsa.2_m_1km_s0..0cm_1979..2013_v1.0.tif  268.26853
136            veg_fapar_proba.v.mar_l.025_250m_s0..0cm_2014..2019_v1.0.tif  253.28066
19     clm_precipitation_sm2rain.may_sd.10_10km_s0..0cm_2007..2018_v1.0.tif  243.96324
156             clm_bioclim.var_chelsa.10_m_1km_s0..0cm_1979..2013_v1.0.tif  239.29262
22          clm_precipitation_sm2rain.oct_m_1km_s0..0cm_2007..2018_v0.2.tif  220.35555
164              clm_bioclim.var_chelsa.3_m_1km_s0..0cm_1979..2013_v1.0.tif  209.76486
24          clm_precipitation_sm2rain.sep_m_1km_s0..0cm_2007..2018_v0.2.tif  207.31070
70               clm_lst_mod11a2.oct.day_sd_1km_s0..0cm_2000..2017_v1.0.tif  172.40199
210                         dtm_vbf_merit.dem_m_500m_s0..0cm_2017_v1.0.tif  171.10056
125            veg_fapar_proba.v.jan_l.025_250m_s0..0cm_2014..2019_v1.0.tif  164.79150
118            veg_fapar_proba.v.dec_l.025_250m_s0..0cm_2014..2019_v1.0.tif  160.65066
25     clm_precipitation_sm2rain.sep_sd.10_10km_s0..0cm_2007..2018_v1.0.tif  147.10143
147                veg_fapar_proba.v.oct_d_250m_s0..0cm_2014..2019_v1.0.tif  141.45931
151                veg_fapar_proba.v.sep_d_250m_s0..0cm_2014..2019_v1.0.tif  133.64483
148            veg_fapar_proba.v.oct_l.025_250m_s0..0cm_2014..2019_v1.0.tif  133.28296
229                  dtm_roughness_merit.dem_m_250m_s0..0cm_2018_v1.0.tif  129.91862
315                  dtm_slope_aw3d30.nasadem_m_30m_s0..0cm_2017_v0.1.tif  128.96582
33                clm_lst_mod11a2.aug.day_m_1km_s0..0cm_2000..2017_v1.0.tif  124.47058
6           clm_precipitation_sm2rain.dec_m_1km_s0..0cm_2007..2018_v0.2.tif  119.47122
283             lcv_landsat.nir_wri.forestwatch_m_30m_s0..0cm_2018_v1.0.tif  116.98678
9      clm_precipitation_sm2rain.feb_sd.10_10km_s0..0cm_2007..2018_v1.0.tif  116.80245
59           clm_lst_mod11a2.mar.daynight_m_1km_s0..0cm_2000..2017_v1.0.tif  110.28752
130            veg_fapar_proba.v.jul_u.975_250m_s0..0cm_2014..2019_v1.0.tif   98.38020
131                veg_fapar_proba.v.jun_d_250m_s0..0cm_2014..2019_v1.0.tif   97.81026
55           clm_lst_mod11a2.jun.daynight_m_1km_s0..0cm_2000..2017_v1.0.tif   96.57759
15     clm_precipitation_sm2rain.jun_sd.10_10km_s0..0cm_2007..2018_v1.0.tif   94.32784
277 clm_cloud.fraction_earthenv.modis.may_p_1km_s0..0cm_2000..2015_v1.0.tif   93.99688
276 clm_cloud.fraction_earthenv.modis.mar_p_1km_s0..0cm_2000..2015_v1.0.tif   93.33088
279 clm_cloud.fraction_earthenv.modis.oct_p_1km_s0..0cm_2000..2015_v1.0.tif   93.15406
```

```
46              clm_lst_mod11a2.jan.day_sd_1km_s0..0cm_2000..2017_v1.0.tif   91.74179
17     clm_precipitation_sm2rain.mar_sd.10_10km_s0..0cm_2007..2018_v1.0.tif   89.32569
159             clm_bioclim.var_chelsa.13_m_1km_s0..0cm_1979..2013_v1.0.tif   89.21204
329         lcv_b12_sentinel.s2l2a_iqr_30m_s0..0cm_2018..2019.s12_v0.1.tif   88.49155
74              clm_lst_mod11a2.sep.day_sd_1km_s0..0cm_2000..2017_v1.0.tif   87.35524
306         lcv_b8a_sentinel.s2l2a_iqr_30m_s0..0cm_2018..2019.s22_v0.1.tif   87.07720
11     clm_precipitation_sm2rain.jan_sd.10_10km_s0..0cm_2007..2018_v1.0.tif   84.71854
333                   veg_f02dar.hv_alos.palsar_m_30m_s0..0cm_2017_v1.0.tif   84.42120
56             clm_lst_mod11a2.jun.night_m_1km_s0..0cm_2000..2017_v1.0.tif   82.64769

Results of ensemble model fitting 'ranger', 'xgboost', 'glmnet', 'deepnet':

Variable: ph_h2o
R-square: 0.818
Fitted values sd: 0.972
RMSE: 0.459

Random forest model:
Call:
stats::lm(formula = f, data = d)

Residuals:
    Min      1Q  Median      3Q     Max
-5.5939 -0.2328 -0.0066  0.2222  4.7477

Coefficients:
               Estimate Std. Error t value Pr(>|t|)
(Intercept)    1.113440   1.164473   0.956 0.338986
regr.ranger    1.032918   0.003138 329.116  < 2e-16 ***
regr.xgboost  -0.014201   0.004185  -3.393 0.000691 ***
regr.cubist    0.049667   0.003709  13.392  < 2e-16 ***
regr.nnet     -0.188570   0.188214  -1.002 0.316398
regr.cvglmnet -0.059763   0.003636 -16.438  < 2e-16 ***
---
Signif. codes:  0 ‘***’ 0.001 ‘**’ 0.01 ‘*’ 0.05 ‘.’ 0.1 ‘ ’ 1

Residual standard error: 0.4591 on 133378 degrees of freedom
Multiple R-squared:  0.8176,    Adjusted R-squared:  0.8176
F-statistic: 1.195e+05 on 5 and 133378 DF,  p-value: < 2.2e-16


Variable importance:
                                                                      variable importance
1           clm_precipitation_sm2rain.annual_m_1km_s0..0cm_2007..2018_v0.2.tif 19293.9099
27              clm_lst_mod11a2.annual.day_sd_1km_s0..0cm_2000..2017_v1.0.tif 10304.5321
26               clm_lst_mod11a2.annual.day_m_1km_s0..0cm_2000..2017_v1.0.tif  8024.7428
```

336	hzn_depth	7534.0473
45	clm_lst_mod11a2.jan.day_m_1km_s0..0cm_2000..2017_v1.0.tif	7269.1869
161	clm_bioclim.var_chelsa.16_m_1km_s0..0cm_1979..2013_v1.0.tif	3521.1232
57	clm_lst_mod11a2.mar.day_m_1km_s0..0cm_2000..2017_v1.0.tif	2959.6055
41	clm_lst_mod11a2.feb.day_m_1km_s0..0cm_2000..2017_v1.0.tif	2388.6211
185	clm_direct.irradiation_solar.atlas.kwhm2.10_m_1km_s0..0cm_2016_v1.tif	2312.8365
159	clm_bioclim.var_chelsa.13_m_1km_s0..0cm_1979..2013_v1.0.tif	1843.7390
208	dtm_vbf_merit.dem_m_1km_s0..0cm_2017_v1.0.tif	1760.6048
13	clm_precipitation_sm2rain.jul_sd.10_10km_s0..0cm_2007..2018_v1.0.tif	1729.2683
268	clm_cloud.fraction_earthenv.modis.annual_m_1km_s0..0cm_2000..2015_v1.0.tif	1515.3343
165	clm_bioclim.var_chelsa.4_m_1km_s0..0cm_1979..2013_v1.0.tif	1505.6802
148	veg_fapar_proba.v.oct_l.025_250m_s0..0cm_2014..2019_v1.0.tif	1303.4942
270	clm_cloud.fraction_earthenv.modis.aug_p_1km_s0..0cm_2000..2015_v1.0.tif	1293.9230
43	clm_lst_mod11a2.feb.daynight_m_1km_s0..0cm_2000..2017_v1.0.tif	1287.9794
272	clm_cloud.fraction_earthenv.modis.feb_p_1km_s0..0cm_2000..2015_v1.0.tif	1278.4984
89	lcv_surf.refl.b05_mod09a1.pc1_m_500m_s0..0cm_2001_v1.0.tif	1277.7427
271	clm_cloud.fraction_earthenv.modis.dec_p_1km_s0..0cm_2000..2015_v1.0.tif	1198.5653
63	clm_lst_mod11a2.may.daynight_m_1km_s0..0cm_2000..2017_v1.0.tif	1156.0320
29	clm_lst_mod11a2.apr.day_m_1km_s0..0cm_2000..2017_v1.0.tif	1149.6378
2	clm_precipitation_sm2rain.apr_m_1km_s0..0cm_2007..2018_v0.2.tif	1122.3751
75	clm_lst_mod11a2.sep.daynight_m_1km_s0..0cm_2000..2017_v1.0.tif	1095.5760
276	clm_cloud.fraction_earthenv.modis.mar_p_1km_s0..0cm_2000..2015_v1.0.tif	1047.5214
278	clm_cloud.fraction_earthenv.modis.nov_p_1km_s0..0cm_2000..2015_v1.0.tif	1029.2173
39	clm_lst_mod11a2.dec.daynight_m_1km_s0..0cm_2000..2017_v1.0.tif	1026.8175
274	clm_cloud.fraction_earthenv.modis.jul_p_1km_s0..0cm_2000..2015_v1.0.tif	1004.9678
53	clm_lst_mod11a2.jun.day_m_1km_s0..0cm_2000..2017_v1.0.tif	987.4694
84	lcv_surf.refl.b02_mod09a1.pc2_m_500m_s0..0cm_2001_v1.0.tif	935.8682
277	clm_cloud.fraction_earthenv.modis.may_p_1km_s0..0cm_2000..2015_v1.0.tif	857.5225
192	dtm_dvm2_merit.dem_m_1km_s0..0cm_2017_v1.0.tif	840.9972
221	dtm_elevation_merit.dem_m_250m_s0..0cm_2017_v1.0.tif	836.2909
73	clm_lst_mod11a2.sep.day_m_1km_s0..0cm_2000..2017_v1.0.tif	834.8211
35	clm_lst_mod11a2.aug.daynight_m_1km_s0..0cm_2000..2017_v1.0.tif	825.5137
71	clm_lst_mod11a2.oct.daynight_m_1km_s0..0cm_2000..2017_v1.0.tif	815.3191
24	clm_precipitation_sm2rain.sep_m_1km_s0..0cm_2007..2018_v0.2.tif	800.2364
269	clm_cloud.fraction_earthenv.modis.apr_p_1km_s0..0cm_2000..2015_v1.0.tif	773.9541
290	dtm_elevation_aw3d30.nasadem_m_30m_s0..0cm_2017_v0.1.tif	766.6710
108	veg_fapar_proba.v.annnual_d_250m_s0..0cm_2014..2019_v1.0.tif	766.0450
191	dtm_dvm_merit.dem_m_2km_s0..0cm_2017_v1.0.tif	762.4431
31	clm_lst_mod11a2.apr.daynight_m_1km_s0..0cm_2000..2017_v1.0.tif	755.9078
163	clm_bioclim.var_chelsa.2_m_1km_s0..0cm_1979..2013_v1.0.tif	737.9077
61	clm_lst_mod11a2.may.day_m_1km_s0..0cm_2000..2017_v1.0.tif	723.3509
203	dtm_twi_merit.dem_m_1km_s0..0cm_2017_v1.0.tif	720.0467
94	lcv_surf.refl.b05_mod09a1.pc6_m_500m_s0..0cm_2001_v1.0.tif	710.1720
308	veg_f02dar.hv_alos.palsar_m_30m_s0..0cm_2007_v1.0.tif	673.5901
263	clm_wind.speed_terraclimate.mar_m_5km_s0..0cm_1998..2018_v1.tif	671.0222
288	dtm_devmean_aw3d30.nasadem.100m_m_30m_s0..0cm_2017_v0.1.tif	665.8133

```
70                 clm_lst_mod11a2.oct.day_sd_1km_s0..0cm_2000..2017_v1.0.tif   665.1251

Results of ensemble model fitting 'ranger', 'xgboost', 'glmnet', 'deepnet':

Variable: sand_tot_psa
R-square: 0.736
Fitted values sd: 22.8
RMSE: 13.7

Random forest model:
Call:
stats::lm(formula = f, data = d)

Residuals:
    Min      1Q  Median      3Q     Max
-80.626  -5.321   0.221   6.071  88.686

Coefficients:
               Estimate Std. Error t value Pr(>|t|)
(Intercept)    6.687471  24.022001   0.278 0.780714
regr.ranger    1.060521   0.003503 302.742  < 2e-16 ***
regr.xgboost  -0.018718   0.004910  -3.812 0.000138 ***
regr.cubist    0.031749   0.003922   8.096 5.73e-16 ***
regr.nnet     -0.161127   0.422746  -0.381 0.703098
regr.cvglmnet -0.028217   0.004462  -6.323 2.57e-10 ***
---
Signif. codes:  0 ‘***’ 0.001 ‘**’ 0.01 ‘*’ 0.05 ‘.’ 0.1 ‘ ’ 1

Residual standard error: 13.65 on 122261 degrees of freedom
Multiple R-squared:  0.736, Adjusted R-squared:  0.736
F-statistic: 6.818e+04 on 5 and 122261 DF,  p-value: < 2.2e-16


Variable importance:
                                                                  variable importance
336                                                              hzn_depth  6407706.1
95                 lcv_surf.refl.b06_mod09a1.pc1_m_500m_s0..0cm_2001_v1.0.tif  2452003.6
301               lcv_b11_sentinel.s2l2a_d_30m_s0..0cm_2018..2019.s22_v0.1.tif  1801324.0
68                 clm_lst_mod11a2.nov.night_m_1km_s0..0cm_2000..2017_v1.0.tif  1739631.6
165                  clm_bioclim.var_chelsa.4_m_1km_s0..0cm_1979..2013_v1.0.tif  1523461.8
19       clm_precipitation_sm2rain.may_sd.10_10km_s0..0cm_2007..2018_v1.0.tif  1389146.1
31               clm_lst_mod11a2.apr.daynight_m_1km_s0..0cm_2000..2017_v1.0.tif  1219948.1
276    clm_cloud.fraction_earthenv.modis.mar_p_1km_s0..0cm_2000..2015_v1.0.tif  1203760.6
13       clm_precipitation_sm2rain.jul_sd.10_10km_s0..0cm_2007..2018_v1.0.tif  1186606.8
208                            dtm_vbf_merit.dem_m_1km_s0..0cm_2017_v1.0.tif  1153595.5
303               lcv_b12_sentinel.s2l2a_d_30m_s0..0cm_2018..2019.s12_v0.1.tif  1126239.4
```

272	clm_cloud.fraction_earthenv.modis.feb_p_1km_s0..0cm_2000..2015_v1.0.tif	1045192.0
332	lcv_b12_sentinel.s2l2a_d_30m_s0..0cm_2018..2019.s22_v0.1.tif	990174.3
168	clm_bioclim.var_chelsa.7_m_1km_s0..0cm_1979..2013_v1.0.tif	981311.5
156	clm_bioclim.var_chelsa.10_m_1km_s0..0cm_1979..2013_v1.0.tif	843897.5
166	clm_bioclim.var_chelsa.5_m_1km_s0..0cm_1979..2013_v1.0.tif	820636.7
39	clm_lst_mod11a2.dec.daynight_m_1km_s0..0cm_2000..2017_v1.0.tif	759930.7
268	clm_cloud.fraction_earthenv.modis.annual_m_1km_s0..0cm_2000..2015_v1.0.tif	717446.0
14	clm_precipitation_sm2rain.jun_m_1km_s0..0cm_2007..2018_v0.2.tif	698575.9
279	clm_cloud.fraction_earthenv.modis.oct_p_1km_s0..0cm_2000..2015_v1.0.tif	688402.1
12	clm_precipitation_sm2rain.jul_m_1km_s0..0cm_2007..2018_v0.2.tif	671624.1
273	clm_cloud.fraction_earthenv.modis.jan_p_1km_s0..0cm_2000..2015_v1.0.tif	670523.5
83	lcv_surf.refl.b02_mod09a1.pc1_m_500m_s0..0cm_2001_v1.0.tif	654835.0
234	dtm_vbf_merit.dem_m_250m_s0..0cm_2017_v1.0.tif	647023.1
46	clm_lst_mod11a2.jan.day_sd_1km_s0..0cm_2000..2017_v1.0.tif	641065.2
1	clm_precipitation_sm2rain.annual_m_1km_s0..0cm_2007..2018_v0.2.tif	640350.0
269	clm_cloud.fraction_earthenv.modis.apr_p_1km_s0..0cm_2000..2015_v1.0.tif	638630.1
271	clm_cloud.fraction_earthenv.modis.dec_p_1km_s0..0cm_2000..2015_v1.0.tif	629429.0
167	clm_bioclim.var_chelsa.6_m_1km_s0..0cm_1979..2013_v1.0.tif	626345.3
161	clm_bioclim.var_chelsa.16_m_1km_s0..0cm_1979..2013_v1.0.tif	611934.9
111	veg_fapar_proba.v.apr_r_250m_s0..0cm_2014..2019_v1.0.tif	607170.9
229	dtm_roughness_merit.dem_m_250m_s0..0cm_2018_v1.0.tif	605217.5
308	veg_f02dar.hv_alos.palsar_m_30m_s0..0cm_2007_v1.0.tif	595912.8
42	clm_lst_mod11a2.feb.day_sd_1km_s0..0cm_2000..2017_v1.0.tif	593624.5
321	lcv_b09_sentinel.s2l2a_iqr_30m_s0..0cm_2018..2019.s22_v0.1.tif	587246.8
54	clm_lst_mod11a2.jun.day_sd_1km_s0..0cm_2000..2017_v1.0.tif	582629.6
101	lcv_surf.refl.b07_mod09a1.pc1_m_500m_s0..0cm_2001_v1.0.tif	580752.9
162	clm_bioclim.var_chelsa.17_m_1km_s0..0cm_1979..2013_v1.0.tif	570026.0
304	lcv_b12_sentinel.s2l2a_iqr_30m_s0..0cm_2018..2019.s22_v0.1.tif	565524.8
34	clm_lst_mod11a2.aug.day_sd_1km_s0..0cm_2000..2017_v1.0.tif	561365.5
281	veg_f02dar.hh_alos.palsar_m_30m_s0..0cm_2007_v1.0.tif	554162.5
51	clm_lst_mod11a2.jul.daynight_m_1km_s0..0cm_2000..2017_v1.0.tif	553083.1
35	clm_lst_mod11a2.aug.daynight_m_1km_s0..0cm_2000..2017_v1.0.tif	548436.9
184	clm_diffuse.irradiation_solar.atlas.kwhm2.100_m_1km_s0..0cm_2016_v1.tif	546090.2
23	clm_precipitation_sm2rain.oct_sd.10_10km_s0..0cm_2007..2018_v1.0.tif	542121.6
62	clm_lst_mod11a2.may.day_sd_1km_s0..0cm_2000..2017_v1.0.tif	541941.6
70	clm_lst_mod11a2.oct.day_sd_1km_s0..0cm_2000..2017_v1.0.tif	538653.4
75	clm_lst_mod11a2.sep.daynight_m_1km_s0..0cm_2000..2017_v1.0.tif	537783.6
50	clm_lst_mod11a2.jul.day_sd_1km_s0..0cm_2000..2017_v1.0.tif	528742.9
38	clm_lst_mod11a2.dec.day_sd_1km_s0..0cm_2000..2017_v1.0.tif	516653.4

Results of ensemble model fitting 'ranger', 'xgboost', 'glmnet', 'deepnet':

Variable: silt_tot_psa
R-square: 0.64
Fitted values sd: 11.9
RMSE: 8.92

```
Random forest model:
Call:
stats::lm(formula = f, data = d)

Residuals:
    Min      1Q  Median      3Q     Max
-63.746  -3.631  -0.526   2.630  72.486

Coefficients:
                Estimate Std. Error t value Pr(>|t|)
(Intercept)   -35.876865  36.887592  -0.973    0.331
regr.ranger     0.948111   0.003874 244.733  < 2e-16 ***
regr.xgboost    0.062717   0.005506  11.391  < 2e-16 ***
regr.cubist     0.025705   0.004747   5.415 6.14e-08 ***
regr.nnet       1.902142   1.968248   0.966    0.334
regr.cvglmnet  -0.028579   0.005799  -4.928 8.32e-07 ***
---
Signif. codes:  0 ‘***’ 0.001 ‘**’ 0.01 ‘*’ 0.05 ‘.’ 0.1 ‘ ’ 1

Residual standard error: 8.915 on 122223 degrees of freedom
Multiple R-squared:  0.6399,    Adjusted R-squared:  0.6399
F-statistic: 4.344e+04 on 5 and 122223 DF,  p-value: < 2.2e-16


Variable importance:
                                                                  variable importance
336                                                              hzn_depth  2342544.8
43             clm_lst_mod11a2.feb.daynight_m_1km_s0..0cm_2000..2017_v1.0.tif   820982.0
59             clm_lst_mod11a2.mar.daynight_m_1km_s0..0cm_2000..2017_v1.0.tif   655784.3
165                clm_bioclim.var_chelsa.4_m_1km_s0..0cm_1979..2013_v1.0.tif   620131.8
13       clm_precipitation_sm2rain.jul_sd.10_10km_s0..0cm_2007..2018_v1.0.tif   509345.4
272    clm_cloud.fraction_earthenv.modis.feb_p_1km_s0..0cm_2000..2015_v1.0.tif   413336.9
275    clm_cloud.fraction_earthenv.modis.jun_p_1km_s0..0cm_2000..2015_v1.0.tif   411241.5
210                        dtm_vbf_merit.dem_m_500m_s0..0cm_2017_v1.0.tif   276745.3
10            clm_precipitation_sm2rain.jan_m_1km_s0..0cm_2007..2018_v0.2.tif   257836.1
164                clm_bioclim.var_chelsa.3_m_1km_s0..0cm_1979..2013_v1.0.tif   251798.7
288               dtm_devmean_aw3d30.nasadem.100m_m_30m_s0..0cm_2017_v0.1.tif   249054.8
300              lcv_b11_sentinel.s2l2a_d_30m_s0..0cm_2018..2019.s12_v0.1.tif   240499.0
191                         dtm_dvm_merit.dem_m_2km_s0..0cm_2017_v1.0.tif   228981.3
278    clm_cloud.fraction_earthenv.modis.nov_p_1km_s0..0cm_2000..2015_v1.0.tif   227571.1
157               clm_bioclim.var_chelsa.11_m_1km_s0..0cm_1979..2013_v1.0.tif   221997.4
268 clm_cloud.fraction_earthenv.modis.annual_m_1km_s0..0cm_2000..2015_v1.0.tif   211767.8
65                  clm_lst_mod11a2.nov.day_m_1km_s0..0cm_2000..2017_v1.0.tif   210742.7
282                      veg_f02dar.hh_alos.palsar_m_30m_s0..0cm_2017_v1.0.tif   210502.5
276    clm_cloud.fraction_earthenv.modis.mar_p_1km_s0..0cm_2000..2015_v1.0.tif   202306.2
```

290	dtm_elevation_aw3d30.nasadem_m_30m_s0..0cm_2017_v0.1.tif	200230.6
101	lcv_surf.refl.b07_mod09a1.pc1_m_500m_s0..0cm_2001_v1.0.tif	195400.5
280	clm_cloud.fraction_earthenv.modis.sep_p_1km_s0..0cm_2000..2015_v1.0.tif	195161.4
2	clm_precipitation_sm2rain.apr_m_1km_s0..0cm_2007..2018_v0.2.tif	194027.2
279	clm_cloud.fraction_earthenv.modis.oct_p_1km_s0..0cm_2000..2015_v1.0.tif	193326.5
326	lcv_b11_sentinel.s2l2a_iqr_30m_s0..0cm_2018..2019.s22_v0.1.tif	192737.0
15	clm_precipitation_sm2rain.jun_sd.10_10km_s0..0cm_2007..2018_v1.0.tif	186500.0
297	lcv_b04_sentinel.s2l2a_iqr_30m_s0..0cm_2018..2019.s22_v0.1.tif	185644.1
163	clm_bioclim.var_chelsa.2_m_1km_s0..0cm_1979..2013_v1.0.tif	182643.1
329	lcv_b12_sentinel.s2l2a_iqr_30m_s0..0cm_2018..2019.s12_v0.1.tif	180803.5
271	clm_cloud.fraction_earthenv.modis.dec_p_1km_s0..0cm_2000..2015_v1.0.tif	179439.2
54	clm_lst_mod11a2.jun.day_sd_1km_s0..0cm_2000..2017_v1.0.tif	178766.8
221	dtm_elevation_merit.dem_m_250m_s0..0cm_2017_v1.0.tif	176816.7
63	clm_lst_mod11a2.may.daynight_m_1km_s0..0cm_2000..2017_v1.0.tif	173548.9
1	clm_precipitation_sm2rain.annual_m_1km_s0..0cm_2007..2018_v0.2.tif	173546.6
299	lcv_b09_sentinel.s2l2a_iqr_30m_s0..0cm_2018..2019.s12_v0.1.tif	170634.3
229	dtm_roughness_merit.dem_m_250m_s0..0cm_2018_v1.0.tif	170129.3
34	clm_lst_mod11a2.aug.day_sd_1km_s0..0cm_2000..2017_v1.0.tif	169657.3
159	clm_bioclim.var_chelsa.13_m_1km_s0..0cm_1979..2013_v1.0.tif	169453.2
38	clm_lst_mod11a2.dec.day_sd_1km_s0..0cm_2000..2017_v1.0.tif	168707.5
160	clm_bioclim.var_chelsa.14_m_1km_s0..0cm_1979..2013_v1.0.tif	167783.0
219	dtm_dvm2_merit.dem_m_250m_s0..0cm_2017_v1.0.tif	167168.9
70	clm_lst_mod11a2.oct.day_sd_1km_s0..0cm_2000..2017_v1.0.tif	166110.9
234	dtm_vbf_merit.dem_m_250m_s0..0cm_2017_v1.0.tif	165551.1
50	clm_lst_mod11a2.jul.day_sd_1km_s0..0cm_2000..2017_v1.0.tif	163821.2
58	clm_lst_mod11a2.mar.day_sd_1km_s0..0cm_2000..2017_v1.0.tif	163745.8
39	clm_lst_mod11a2.dec.daynight_m_1km_s0..0cm_2000..2017_v1.0.tif	161704.0
213	dtm_convergence_merit.dem_m_250m_s0..0cm_2018_v1.0.tif	160570.2
318	lcv_b02_sentinel.s2l2a_iqr_30m_s0..0cm_2018..2019.s12_v0.1.tif	158744.4
283	lcv_landsat.nir_wri.forestwatch_m_30m_s0..0cm_2018_v1.0.tif	158553.2
158	clm_bioclim.var_chelsa.12_m_1km_s0..0cm_1979..2013_v1.0.tif	158014.4
